# Greater molecular potential for glucose metabolism in adipose tissue and skeletal muscle of women compared with men

**DOI:** 10.1096/fj.202302377R

**Published:** 2024-07-31

**Authors:** Trine S. Nicolaisen, Kim A. Sjøberg, Christian S. Carl, Erik A. Richter, Bente Kiens, Andreas M. Fritzen, Anne‐Marie Lundsgaard

**Affiliations:** ^1^ The August Krogh Section for Molecular Physiology, Department of Nutrition, Exercise and Sports, Faculty of Science University of Copenhagen Copenhagen Denmark; ^2^ Faculty of Health and Medical Sciences, Novo Nordisk Foundation Center for Basic Metabolic Research University of Copenhagen Copenhagen Denmark; ^3^ Department of Biomedical Sciences, Faculty of Health and Medical Sciences University of Copenhagen Copenhagen Denmark; ^4^ Novo Nordisk A/S Søborg Denmark

**Keywords:** adipocytes, adiponectin, gender, glucose metabolism, insulin action, obesity, skeletal muscle

## Abstract

Women typically have less muscle mass and more fat mass than men, while at the same time possessing similar or even greater whole‐body insulin sensitivity. Our study aimed to investigate the molecular factors in primarily adipose tissue, but also in skeletal muscle, contributing to this sex difference. In healthy, moderately active premenopausal women and men with normal weight (28 ± 5 and 23 ± 3 years old; BMI 22.2 ± 1.9 and 23.7 ± 1.7) and in healthy, recreationally active women and men with overweight (32.2 ± 6 and 31.0 ± 5 years old; BMI 29.8 ± 4.3 & 30.9 ± 3.7) matched at age, BMI, and fitness level, we assessed insulin sensitivity and glucose tolerance with a hyperinsulinemic–euglycemic clamp or oral glucose tolerance test and studied subcutaneous adipose tissue and skeletal muscle samples with western blotting. Additionally, we traced glucose‐stimulated glucose disposal in adipose tissues of female and male C57BL/6J littermate mice aged 16 weeks and measured glucose metabolic proteins. Our findings revealed greater protein expression related to glucose disposal in the subcutaneous adipose tissue (AKT2, insulin receptor, glucose transport 4) and skeletal muscle (hexokinase II, pyruvate dehydrogenase) in women compared to matched men with normal weight and with overweight. This increased protein capacity for glucose uptake extended to white adipose tissues of mice accompanied with ~2‐fold greater glucose uptake compared to male mice. Furthermore, even in the obese state, women displayed better glucose tolerance than matched men, despite having 46% body fat and 20 kg less lean mass. In conclusion, our findings suggest that the superior potential for glucose disposal in female subcutaneous adipose tissue and skeletal muscle, driven by greater expression of various glucose metabolic proteins, compensates for their lower muscle mass. This likely explains women's superior glucose tolerance and tissue insulin sensitivity compared to men.

## Graphical abstract

The graphical abstract shows how we find greater expression of various glucose metabolic proteins in subcutaneous adipose tissue and skeletal muscle of both lean women and women with obesity compared to matched men. In addition, we observe higher glucose uptake in adipose tissues and skeletal muscle in female compared with male mice.

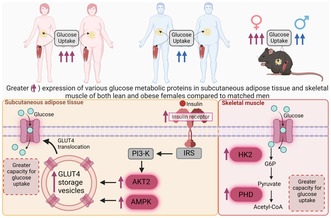

**Abbreviations**
3H‐2DG: 3H‐2‐deoxyglucose
3H‐2DG‐6‐P: 3H‐2‐dexoyglucose‐6‐phosphate
AMPK: AMP‐activated protein kinase
ATGL: adipose triglyceride lipase
BMI: body mass index
CAV1: caveolin 1
CAV3: caveolin 3
CD36: cluster of differentiation 36/SR‐B3 (previously SR‐B2)
DXA: dual‐energy X‐ray absorptiometry
EchoMIR: magnetic resonance imaging
eWAT: epididymal white adipose tissue
FA: fatty acid
HKII: hexokinase II
HOMA‐IR index: homeostatic model assessment for insulin resistance index
HSL: hormone‐sensitive lipase
iAUC: incremental area under the curve
iBAT: intrascapular brown adipose tissue
IR: insulin receptor
iWAT: inguinal white adipose tissue
LBM: lean body mass
OGTT: oral glucose tolerance test
PDH‐E1α: pyruvate dehydrogenase E1‐alpha
RER: respiratory exchange ratio
SCAT: subcutaneous adipose tissue
VCO2: CO2 production
VO2: oxygen consumption
VO2peak: peak oxygen uptake

## 1 INTRODUCTION

Women typically have less lean mass and more body fat than men when compared at similar body mass index (BMI). At the same time, whole‐body insulin sensitivity, determined in hyperinsulinemic–euglycemic clamp studies as glucose infusion rate expressed per kg body mass, is found to be similar^1^, ^2^, ^3^, ^4^, ^5^, ^6^ or 38%–41% greater^7^, ^8^ in lean, premenopausal women when compared with BMI‐matched men. Skeletal muscle is known to account for 60%–70% of total insulin‐stimulated glucose disposal,^9^ and when investigated in a sex‐comparative manner, insulin‐stimulated glucose uptake into the femoral muscle was 47% greater in women than men when expressed per kg of muscle, measured by use of ^18^F‐FDG‐PET.^7^ We have measured insulin‐stimulated glucose uptake by the arteriovenous balance technique across the leg, and obtained 29%–35% greater leg glucose disposal in lean women compared with men, when related to the amount of lean mass in the leg.^10^, ^11^ These observations suggest that insulin effectiveness to stimulate glucose uptake is greater per unit mass of female compared with male skeletal muscle. Notably, in women the mass of adipose tissue is typically equal to or even greater than the mass of skeletal muscle,^12^ and it is intriguing whether a sexual dimorphism in glucose uptake is also present in the adipose tissue compartment. Adipocytes are, as myocytes, insulin‐sensitive cells. During insulin stimulation, glucose uptake rate per mass of subcutaneous adipose tissue (SCAT) amounted a substantial extent of that of skeletal muscle (27%–40%) assessed by ^18^F‐FDG‐PET in groups of mixed women and men.^9^, ^13^ While not evaluated in a sex‐specific manner in these studies, this underscores a significant contribution of adipose tissue to whole‐body glucose disposal during insulin‐stimulated conditions. In vitro, basal and insulin‐stimulated glucose uptake in primary adipocytes, obtained from SCAT of both lean and individuals with obesity, were higher in adipocytes from females than males when expressed per cell number.^14^, ^15^ Moreover, when individuals were divided based on sex in an in vivo ^18^F‐FDG‐PET study of adipose tissue glucose uptake, lean premenopausal women showed 121% greater insulin‐stimulated glucose uptake per mg abdominal SCAT when compared with lean, age‐matched men.^16^ These findings indicate that adipose tissue plays a greater role in glucose disposal in women than in men, not only due to a greater total fat mass, but also due to a greater uptake capacity per adipocyte or tissue mass. Interestingly, the molecular mechanisms underpinning this increased capacity for glucose uptake into adipose tissue in women compared with men are not fully elucidated.

The primary aim of this study was to elucidate the molecular signature responsible for glucose uptake and handling in abdominal SCAT and skeletal muscle of women compared with men. This was investigated in lean premenopausal women and men matched at age, BMI, and fitness level. As cross‐sectional studies report that women better maintain glucose homeostasis during conditions of increased fat deposition than men,^17^, ^18^ we also evaluated whether a sex‐specific signature of glucose metabolic proteins was evident in abdominal SCAT and skeletal muscle of healthy premenopausal women and men with obesity, matched at the same criteria as the lean cohort. Subcutaneous fat was chosen for investigation as it by mass represents the greatest adipose depot of both women and men. Peripheral and hepatic insulin actions were assessed in the lean individuals, to distinguish between their relative roles in whole‐body insulin action in carefully matched women and men. In the women and men with overweight, glycemic control was evaluated by an oral glucose tolerance test (OGTT). Finally, we took advantage of a mouse model to trace glucose‐stimulated glucose uptake into different adipose compartments of female and male mice.

## 2 MATERIALS AND METHODS

The human studies in protocol 1 (KF01261127) and protocol 2 (H‐3‐2010‐058) were approved by the Copenhagen Ethics Committee. The animal study in protocol 3 complied with the EU convention for the protection of vertebra used for scientific purposes and was approved by the Danish Animal Experiments Inspectorate.

### 2.1 Protocol 1. Premenopausal lean women and matched men

**FIGURE 1:**
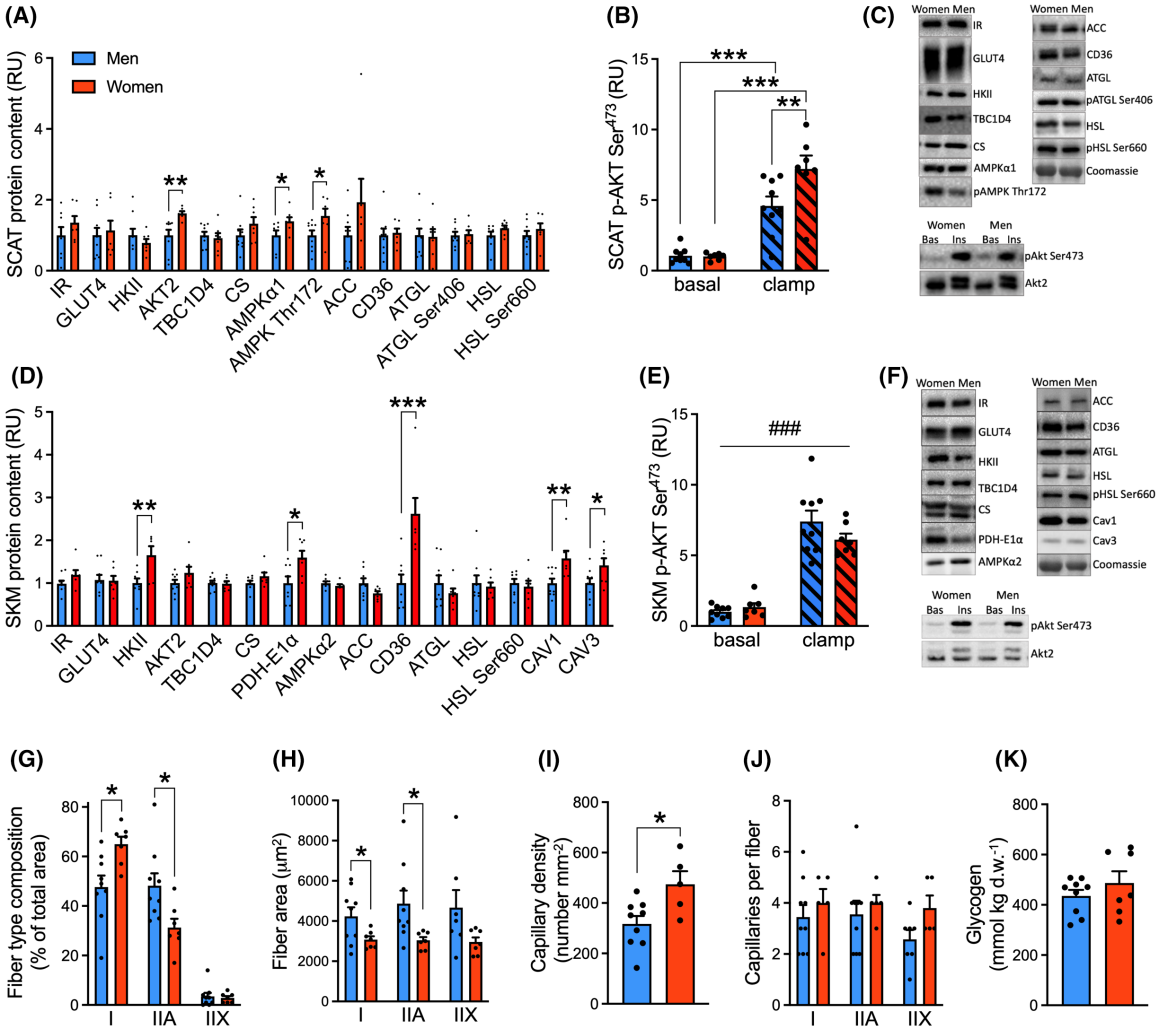
Greater capacity for glucose disposal in female adipose tissue and skeletal muscle. Data from biopsies of abdominal subcutaneous adipose tissue (SCAT) and vastus lateralis muscle (SKM) of lean premenopausal women (red) and matched men (blue), obtained at the postabsorptive state (non‐shaded bars) or at the end of the clamp (shaded bars in B and E). (A, B) content and phosphorylation levels of SCAT metabolic proteins. (C) representative western blots. (D, E), protein content or phosphorylation levels of SKM metabolic proteins. (F) Representative western blots, data on beta‐actin, and total protein are shown in Figure S2. Western blot data are net intensities expressed as relative units (RU) normalized to men (basal). (G) fiber type composition expressed as percent of total analyzed area. (H) individual fiber area. (I) capillary number per given area and (J) capillary number per fiber, all determined by histochemistry. (K) skeletal muscle homogenate glycogen content. Unpaired *t*‐tests were applied in A–D, and G–K. A two‐way RM ANOVA was applied in B and E with Bonferroni multiple comparisons test. *p < .05, **p < .01, ***p < .001, women vs. men. ^###^ *p* < .001 main effect of insulin. Data are mean ± SEM. *n* = 7 in women and *n* = 9 in men. For the men, GLUT4, HKII, AKT2, TBC1D4, PDH‐E1α, CD36, HSL, HSL Ser660, p‐AKT Ser^473^ of the SKM protein data in D and E have previously been published.^19^, ^20^

**FIGURE 2:**
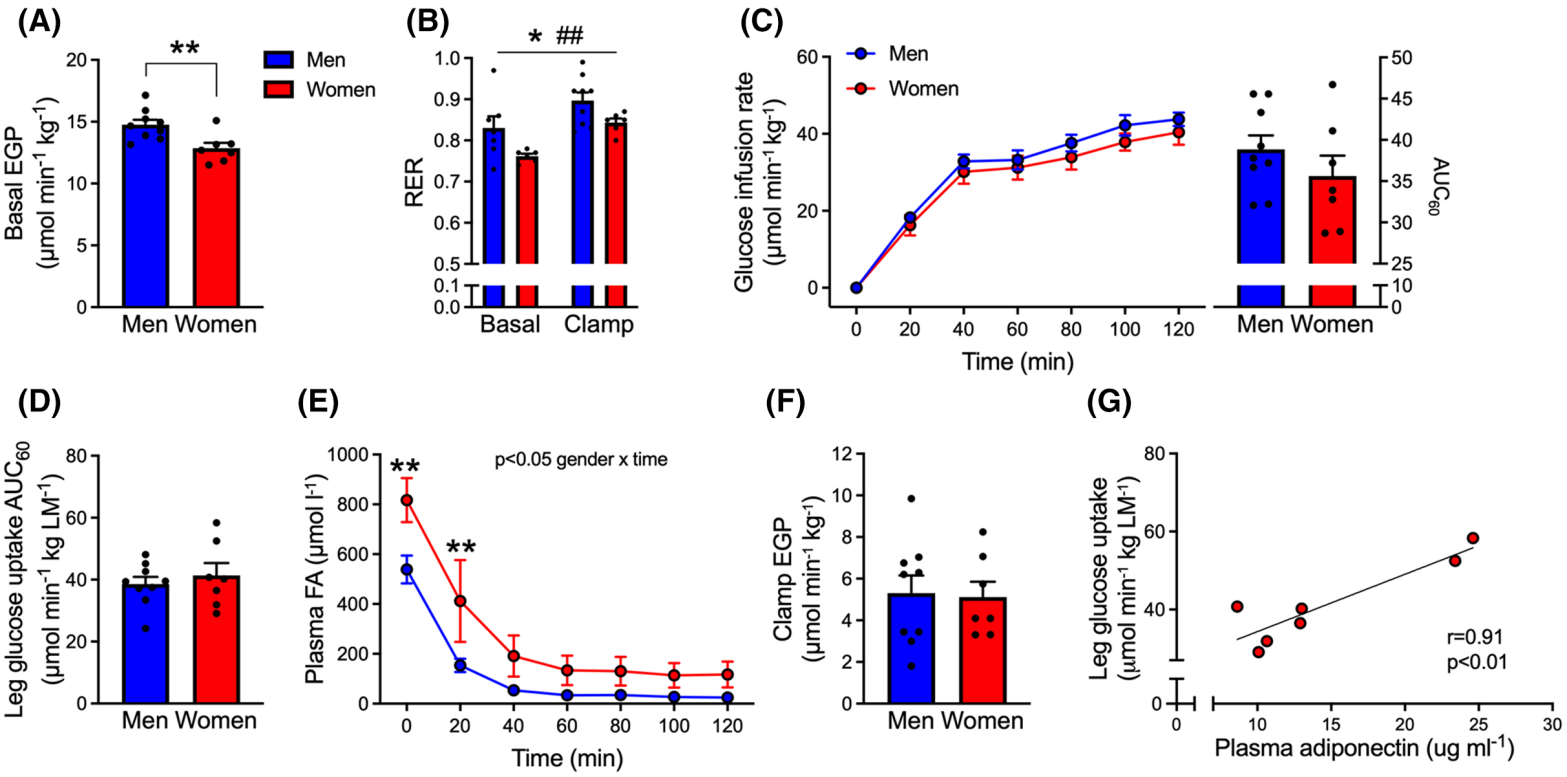
Similar whole‐body and peripheral insulin action in lean premenopausal women and matched men, despite lower lean mass in women. (A) Endogenous glucose production (EGP) in the basal (postabsorptive) state. (B) whole‐body respiratory exchange ratio (RER) obtained with indirect calorimetry before and at the end of the clamp. (C) glucose infusion rate expressed per kg body mass. (D) insulin‐stimulated leg glucose uptake expressed per kg leg mass (LM). Data in C (right bar graph) and D represent data obtained during the last 60 min of the clamp. (E) arterial plasma FA concentration before and during the clamp. (F) EGP during insulin‐stimulation (G) scatter plot illustrating the association between postabsorptive plasma adiponectin concentration and insulin‐stimulated leg glucose uptake in women. Unpaired *t*‐tests were applied in A, C (right bar graph), D and F. A two‐way RM ANOVA was applied in B, C (left curve), and E with Bonferroni multiple comparisons test. In G, Pearson's correlation analysis was applied. *p < .05, **p < .01, effect or main effect (B) of sex, ^##^ *p* < .01 main effect of insulin. Data are mean ± SEM. *n* = 7 in women and *n* = 9 in men (in G, *n* = 6 in women due to measurement error). For the men, data in A–D have been previously published.^19^, ^20^

Healthy, young women (*n* = 7) and men (*n* = 9) were recruited. Participants were moderately physically active, and women and men were matched for age, BMI, and peak oxygen uptake (VO_2peak_) expressed relative to lean body mass (LBM) (Table 1). Women were eumenorrheic and did not use oral contraception. Experiments were conducted during the midfollicular phase of the menstrual cycle. For the male participants, a subset of data has been previously published,^19^, ^20^ as specified in the legend of Table 1, Figures 1, and 2.

**TABLE 1:** Characteristics of the lean premenopausal women and men in protocol 1 and premenopausal women and men with overweight in protocol 2.

	Lean individuals		Individuals with overweight	
	Women (*n* = 7)	Men (*n* = 9)	Women (*n* = 16)	Men (*n* = 10)
Age, years	28 ± 5	23 ± 3	32.2 ± 6	31.0 ± 5
Body mass (BM), kg	62.9 ± 9.9**	79.1 ± 7.9	85.3 ± 15.5**	101.6 ± 9.1
BMI, kg m^−2^	22.2 ± 1.9	23.7 ± 1.7	29.8 ± 4.3	30.9 ± 3.7
Body fat, %	27.3 ± 5.3***	16.2 ± 4.1	46.1 ± 5.7***	36.4 ± 5.6
Lean body mass (LBM), kg	45.3 ± 6.0***	66.1 ± 4.8	45.3 ± 4.4***	65.3 ± 7.3
LBM/BM, %	72.4 ± 5.6***	83.8 ± 4.1	53.9 ± 5.7***	63.6 ± 5.6
Fat mass, kg	17.1 ± 5.0	13.0 ± 4.2	40.0 ± 12.0	36.3 ± 8.0
Leg mass, kg	11.6 ± 2.2*	14.0 ± 1.8	–	–
Lean leg mass, kg	8.0 ± 1.4***	11.5 ± 1.2	–	–
Lean leg mass/leg mass, %	69.1 ± 3.7***	82.4 ± 4.8	–	–
Leg fat mass, kg	3.6 ± 0.9	2.5 ± 0.9	–	–
Leg fat mass/leg mass, %	30.9 ± 3.7***	17.6 ± 4.8	–	–
VO_2peak_, mL kg BM^−1^ min^−1^	45.8 ± 3.9**	52.3 ± 4.6	25.6 ± 5.6(*)	29.7 ± 6.1
VO_2peak_, mL kg LBM^−1^ min^−1^	63.3 ± 4.9	62.4 ± 3.3	47.0 ± 8.5	47.1 ± 10.7
HOMA‐IR index	1.3 ± 0.2	1.7 ± 0.3	2.6 ± 0.6	3.0 ± 0.5

*Note*: Body composition was determined by dual‐energy X‐ray absorptiometry. An unpaired *t*‐test was applied. (*) *p* = .08, *p < .05, **p < .01, ***p < .001 women versus men. Data are mean ± SD. VO_2peak_, peak oxygen uptake.

Before the assessment of insulin sensitivity and tissue biopsies, participants underwent eight days intake of a weight‐maintenance control diet (62 energy% (E%) carbohydrate, 14E% protein, and 24E% fat), resembling their habitual diet. All food items were weighed to 1 gram of accuracy, pre‐packed by meal, and delivered to the participants. On the experimental day, participants ingested a small standard mixed meal (10% of daily energy requirement) at 5 a.m.^19^ Participants arrived at the institute at 8 a.m. by passive transport, having abstained from strenuous physical activity 72 h before. After 30 min of supine rest, catheters were inserted into an antecubital vein, as well as into the femoral artery and vein under local anesthesia, and blood was obtained for ^2^H background enrichment. Then, a bolus injection of [6,6‐^2^H_2_] glucose tracer (3.203 mg kg^−1^) was given, followed by a constant intravenous infusion (0.055 mg kg^−1^ min^−1^) over 120 min, to determine endogenous glucose production. Then, postabsorptive arterial blood samples (basal samples) were drawn (6 h following the small early morning meal). Thereafter, a 120 min hyperinsulinemic–euglycemic clamp was initiated by a bolus of insulin (9.0 mU kg^−1^) (Actrapid, Novo Nordisk, DK) followed by a constant intravenous insulin infusion rate (1.4 mU insulin kg^−1^ min^−1^). During the clamp, 20% glucose solution enriched with 1.9% glucose tracer was infused to maintain plasma glucose at the target concentration, determined by two basal arterial blood samples. Arteriovenous blood samples were obtained before and every 20 min during the clamp, together with femoral arterial blood flow by ultrasound (Philips Ultrasound, US) to calculate leg glucose uptake. Whole‐body non‐protein respiratory exchange ratio (RER) was calculated as CO_2_ production (VCO_2_) divided by oxygen consumption (VO_2_), assessed by indirect calorimetry at basal conditions and during the clamp. RER of 1.0 reflects 100% glucose oxidation, and 0.7 reflects 100% fat oxidation. To investigate protein expression, biopsies were obtained from the abdominal periumbilical SCAT and the vastus lateralis muscle in the basal and insulin‐stimulated state (before and at the end of the clamp).

### 2.2 Protocol 2. Premenopausal women and age‐matched men with overweight

Premenopausal women (*n* = 16) and men (*n* = 10) with overweight matched at age, BMI (30 ± 4 and 31 ± 4 kg m^−2^, respectively), and peak oxygen uptake (VO_2peak_) expressed relative to lean body mass (LBM) (Table 1) were studied. Oral glucose tolerance and tissue biopsies from the abdominal periumbilical SCAT and the vastus lateralis muscle were obtained in the overnight‐fasted state, on two separate days. The 26 individuals formed part of a cohort of 181 participants described in a previous study.^21^ The present individuals were selected from the cohort with criteria of age ≤42 years (to include only premenopausal women), and completion of tissue biopsies as well as an oral glucose tolerance test (OGTT). For the OGTT, 75 g glucose in 300 mL water was provided, and antecubital venous blood was drawn every 30 min for 120 min for the determination of plasma glucose and insulin concentrations.

### 2.3 Methods applied in protocol 1 and 2

The composition of lean and fat mass at the whole‐body level was determined by dual‐energy X‐ray absorptiometry (DXA; Lunar Corporation, US) in both protocol 1 and 2, while leg lean and fat masses were also determined by DXA in protocol 1. VO_2peak_ was in protocol 1 measured during an incremental bicycle test to exhaustion, and accepted if RER was >1.15 and/or a plateau in VO_2_ was achieved. In protocol 2, VO_2peak_ was predicted by heart rate and VO_2_ extrapolations from a submaximal two stage bicycle test.^22^ Biopsies were obtained from the abdominal periumbilical SCAT and the vastus lateralis muscle by a modified Bergström needle with suction, under local anesthesia (Xylocaine 2%; AstraZeneca, Copenhagen, DK). SCAT and skeletal muscle biopsies were flushed through the needle with ice‐cold saline and immediately frozen in liquid nitrogen and stored at −80°C until further analysis. In protocol 1, one part of the muscle biopsy was mounted in embedding medium (Tissue‐tek) and frozen in isopentane cooled by liquid nitrogen and stored at −80°C until histochemical analysis.

#### 2.3.1 Human histochemical muscle fiber type analysis

Serial cross sections (10 μm) of the embedded muscle were cut and stained for myofibrillar ATPase to identify the different fiber types: MHCI, MHCIIA, and MHCIIX,^23^ and capillaries were visualized using a staining method against caveolin‐1.^24^ Quantification was made by TEMA software (CheckVision, DK).

#### 2.3.2 Human muscle biochemical analyses

The skeletal muscle tissue was freeze‐dried and dissected free for connective tissue, blood, and fat under microscope before the biochemical analyses.

*Human muscle glycogen content* was determined fluorometrically in homogenates as glycosyl units after acid hydrolysis.^25^

#### 2.3.3 Western blotting (human and mouse tissue samples)

Samples of SCAT and skeletal muscle, as well as mouse iWAT and eWAT, were homogenized in ice‐cold modified GSK3 buffer as described previously.^26^ After 20‐min centrifugation at 16 000 *g* at 4°C, supernatant was collected. For adipose tissue samples, samples were re‐centrifuged after removal of the upper fatty layer. Protein content was determined in triplicates with the bicinchoninic acid method (BCA no. 23225, Pierce, US). Samples were heated to 96°C in Laemmli buffer and total protein and phosphorylation levels measured by semidry immunoblotting. Inter gel variation was controlled by two internal controls on each gel. For skeletal muscle samples, beta‐actin was used as a loading control, and no sex difference was obtained in the two groups (Figure S2). For the SCAT samples, Pierce Reversible Stain (Thermo‐Fisher Scientific, US) was applied on the membrane, and equal total protein amounts were shown for each group (Figure S2). The primary antibodies are shown in Supplemental Table S1. The full unedited blots are included as Supplemental Figure S1. Band intensities were quantified by Image Lab software (Bio‐Rad, US). Data are shown as net intensities, expressed in relative units compared to the average of the male group.

#### 2.3.4 Human plasma variables

Glucose concentration was measured on ABL800 (Radiometer Medical, DK). Enzymatic colorimetric analyses were used to measure plasma fatty acid (FA) (Wako Chemicals, GE) concentrations on a Hitachi 912 automatic analyzer (Boehringer, GE). Plasma insulin and c‐peptide concentrations were measured by ELISA (ALPCO, US). Plasma catecholamines were analyzed by a RIA kit (Immuno‐Biological Laboratories, DE). Estradiol, leptin, and adiponectin concentrations were measured on an AutoDELFIA (Perkin Elmer, US) automated analyzer. Plasma enrichment of ^2^H glucose was measured using liquid chromatography mass spectrometry (ThermoQuest Finnegan AQA, US).^27^

### 2.4 Protocol 3. Female and male mouse study

Female and male C57BL/6J littermate mice aged 16 weeks (Janvier, FR) were housed in groups of four, with 12 h dark–light cycle at 23.5°C, and had free access to chow diet (65E% carbohydrates, 24E% protein, and 11E% fat) (Altromin 1324, Brogaarden, DK). One week prior the experiment, lean and fat mass were measured by magnetic resonance imaging (EchoMIR 4‐in‐1; EchoMRI, US). On the experimental day, mice were fasted for 4 h and glucose uptake into different adipose tissue depots was investigated following glucose administration, by use of ^3^H‐2‐deoxyglucose (^3^H‐2DG) tracer. Blood glucose was assessed in mixed tail blood with a glucometer (Contour XT; Bayer, DE), and blood sampled for ^3^H‐enrichment from the tail before (0 min) and at 15, 30, and 45 min after an intraperitoneal injection of 2 g/kg BW glucose plus trace amounts of ^3^H‐2DG (0.6 μCi/g BW), dissolved in saline (10 μL/g BW). An aliquot of blood obtained at 15 min was used to determine plasma insulin concentration (mouse insulin ELISA, ALPCO, US). At 45 min following glucose injection, mice were sacrificed by cervical decapitation, and inguinal white adipose tissue (iWAT), epididymal white adipose tissue (eWAT), and intrascapular brown adipose tissue (iBAT) were excised and snap frozen in liquid nitrogen.

The accumulation of ^3^H‐2‐dexoyglucose‐6‐phosphate (^3^H‐2DG‐6‐P) was determined in 17–30 mg of each adipose tissue by the precipitation method.^28^ ^3^H‐2DG radioactivity was measured in 3 μL blood and in tissue samples by scintillation counting. Tissue glucose clearance was calculated by dividing tissue‐specific ^3^H‐2‐DG‐6‐P DPM accumulation over 45 min by the average blood ^3^H‐2DG DPM during this period,^24^ and glucose clearance multiplied by the average blood glucose concentration during the 45 min to calculate glucose uptake. Of the remaining iWAT and eWAT samples, ~60 mg was used for western blotting analysis.

### 2.5 Statistics

All data are presented as individual values and mean ± SEM, except subject characteristics in Table 1 (mean ± SD). The applied statistics are noted in each figure legend. A significance level of *p* < .05 was chosen. Analyses were performed in Prism version 9 (GraphPad, US).

### 2.6 Calculations

Insulin‐stimulated leg glucose uptake was calculated as the arteriovenous blood glucose difference multiplied by blood flow and related to leg mass (total, lean, and fat mass of the leg). Glucose infusion rate and leg glucose uptake were expressed as average of the last 60 min of the clamp. Endogenous glucose production was calculated from plasma enrichment at triple measurements during the last 20 min of the basal and clamp period using Steele's equation.^29^ Insulin clearance was calculated as insulin infusion rate/([insulin]_clamp_‐([c‐peptide]_clamp_ [insulin]_basal_/[c‐peptide]_basal_)).

## 3 RESULTS

### 3.1 Greater capacity at the protein level for glucose disposal in adipose tissue and skeletal muscle of lean women

To study whether women express higher protein levels of the machinery implicated in glucose uptake and handling in adipose tissue and skeletal muscle, biopsies from the basal (postabsorptive) state and during insulin stimulation were studied. In the adipose tissue, basal protein content of AKT2 was 62% higher in women than in men (Figure 1A), and during insulin stimulation, 101% greater phosphorylation of AKT at Ser^473^ was obtained in women compared with men (Figure 1B), indicating a greater potential for the activation of insulin‐induced signaling toward glucose uptake. The phosphorylation level per AKT2 protein was not different between sexes (data not shown). Interestingly, another protein involved in insulin action in adipose tissue is AMP‐activated protein kinase α1 (AMPKα1). AMPKα1 protein was 39% higher and AMPK Thr^172^ phosphorylation 54% higher in women than men (Figure 1A). No sex difference was obtained when phosphorylation was expressed per AMPKα1 protein (data not shown). In a study by Wu and colleagues,^30^ they suggested an involvement of AMPK in the stimulatory effect of adiponectin on basal and insulin‐stimulated adipose glucose uptake. In the present study, we found that the postabsorptive plasma adiponectin level was 110% higher in women than men (Table 2), and it could be hypothesized that greater adiponectin‐induced AMPK activation could contribute to increased glucose uptake in female adipose tissue.

**TABLE 2:** Arterial plasma variables of young premenopausal women and men.

Arterial plasma/serum variables	Women	Men
Plasma estradiol, nmol L^−1^	0.27 ± 0.09	0.11 ± 0.01
Serum adiponectin, μg mL^−1^	14.8 ± 2.5*	7.1 ± 1.5
Plasma leptin, pg mL^−1^	10.4 ± 1.4***	3.1 ± 0.6
Plasma epinephrine, mmol L^−1^	0.44 ± 0.09	0.27 ± 0.08
Plasma norepinephrine, mmol L^−1^	1.05 ± 0.5	1.31 ± 0.48
Plasma glucose, mmol L^−1^	5.3 ± 0.1*	5.8 ± 0.1
Plasma insulin, μU mL^−1^		
*Basal*	5.3 ± 1.0	6.4 ± 1.0
*Clamp*	95.5 ± 5.5	93 ± 3.1
Plasma c‐peptide, pmol L^−1^		
*Basal*	323 ± 64	299 ± 31
*Clamp*	217 ± 35	230 ± 20
Insulin clearance, mL^−1^ kg^−1^ min^−1^		
*Clamp*	15.7 ± 1.1	16.0 ± 0.1

*Note*: Plasma and serum samples were obtained in the postabsorptive state following eight days of control diet. Unpaired *t*‐tests were applied. *p < .05, ***p < .001 women versus men. Data are mean ± SEM. *n* = 7 women and *n* = 9 men. Gastric inhibitory polypeptide (GIP), peptide YY (PYY), and glucagon‐like peptide‐1 (GLP‐1). For the men, plasma glucose, insulin, c‐peptide, FA, triacylglycerol, epinephrine, norepinephrine, and adiponectin have been previously published.^19^, ^20^

Postabsorptive lipolytic capacity in the adipose tissue appeared similar between sexes, as no differences were obtained in protein content or basal phosphorylation of adipose triglyceride lipase (ATGL) and hormone‐sensitive lipase (HSL) (Figure 1A).

When protein contents were evaluated in basal skeletal muscle biopsies (Figure 1D), hexokinase II (HKII) protein content was 65% higher in women than in men, which was not observed in adipose tissue. This suggests a greater capacity for intramyocellular phosphorylation of glucose and in turn a greater gradient for trans‐sarcolemmal glucose uptake in skeletal muscle. Furthermore, muscle pyruvate dehydrogenase E1‐alpha (PDH‐E1α) protein content was 60% higher in women than in men, indicating a greater capacity for glycolytic flux via pyruvate conversion to acetyl‐CoA, and hence oxidative utilization of glucose. In contrast to adipose tissue, Akt2 protein expression and insulin‐stimulated skeletal muscle AKT Ser^473^ phosphorylation were similar between sexes (Figure 1E), implying that the proximal insulin signaling cascade was similarly activated in muscle tissue of women and men. Interestingly, we found a higher expression of caveolin 3 (CAV3), a caveolae marker, which colocalizes with GLUT4 and impacts on glucose uptake,^31^ was 42% higher in women than in men (Figure 1D).

In skeletal muscle, a greater capacity for uptake of FA substrates was evident in women compared with men together with the greater capacity for glucose oxidation. Enabling greater transmembrane FA uptake in women, skeletal muscle cluster of differentiation 36/SR‐B3 (previously SR‐B2) (CD36) protein content was 162% higher in women than in men (Figure 1D), supporting our previous findings.^10^

The sex‐specific metabolic protein expression (greater HKII, PDH‐E1α, CAV3, and CD36) was associated with an (already well‐known) oxidative fiber type expression in female muscle. Thus, the percentage of type I fibers covering the muscle area analyzed by histochemistry was 65 ± 3% in women compared to 48 ± 5% in men, and the area percentage of type IIA fibers was hence lower in women (Figure 1G). For the individual fibers, women displayed 27% and 37% smaller muscle fiber cross‐sectional area of type I and type IIA fibers, respectively (Figure 1H). The capillary density was 49% greater in women than in men, explained by the smaller individual fiber area in women (Figure 1I,J), and the protein content of the endothelial marker caveolin 1 (CAV1) was 58% higher in women than men (Figure 1D). Despite the lower glycolytic fiber type expression in female muscle, no sex difference in basal glycogen concentration of muscle homogenates was found, indicating similar glucose storage (Figure 1K). Overall, women seem to have a higher expression of several proteins involved in glucose metabolism in both the subcutaneous adipose tissue and skeletal muscle.

### 3.2 Similar whole‐body glucose disposal despite lower lean mass in women

Evaluation of postabsorptive glucose homeostasis revealed that postabsorptive blood glucose concentration was lower in lean women than men (5.3 ± 0.1 vs. 5.8 ± 0.1 mmol L^−1^) (Table 2), as was the basal endogenous glucose production (Figure 2A). The HOMA‐IR index was 1.3 ± 0.2 in women and 1.7 ± 0.3 in men (Table 1, NS). The lower blood glucose was found concomitantly with 52% higher postabsorptive plasma FA concentration in women than in men (Table 2), and a lower RER in women (0.76 ± 0.01) than in men (0.83 ± 0.03) (Figure 2B), indicating higher basal FA oxidation in women.

When insulin sensitivity was assessed during the hyperinsulinemic–euglycemic clamp, the glucose infusion rate expressed per kg body mass was similar in women and men (Figure 2C), despite women having lower total lean mass relative to body mass than men (72 ± 6% vs. 84 ± 4%) (Table 1). Accordingly, insulin‐stimulated glucose uptake in the leg expressed per kg leg mass was similar between sexes (Figure 2D), despite that lean leg mass expressed relative to leg mass comprised 69 ± 4% in female versus 82 ± 5% in male individuals (Table 1). A greater capacity for glucose uptake in both female adipose tissue and skeletal muscle may thus enable similar whole‐body insulin sensitivity despite lower lean mass. A positive correlation was obtained between plasma adiponectin concentration and the insulin‐stimulated leg glucose uptake in women (*r* = .91, *p* < .01) (Figure 1G), but not in men (*r* = .44, *p* = .23).

In response to insulin, there was a similar increase in whole‐body glucose oxidation (Figure 2B), and plasma FA levels were suppressed to the same extent in both sexes (Figure 2E). The endogenous glucose production during insulin stimulation, plasma insulin, and insulin clearance rate during the clamp was not different between sexes (Figure 2F and Table 2).

### 3.3 Greater capacity at the protein level for glucose disposal in adipose tissue of overweight women

**FIGURE 3:**
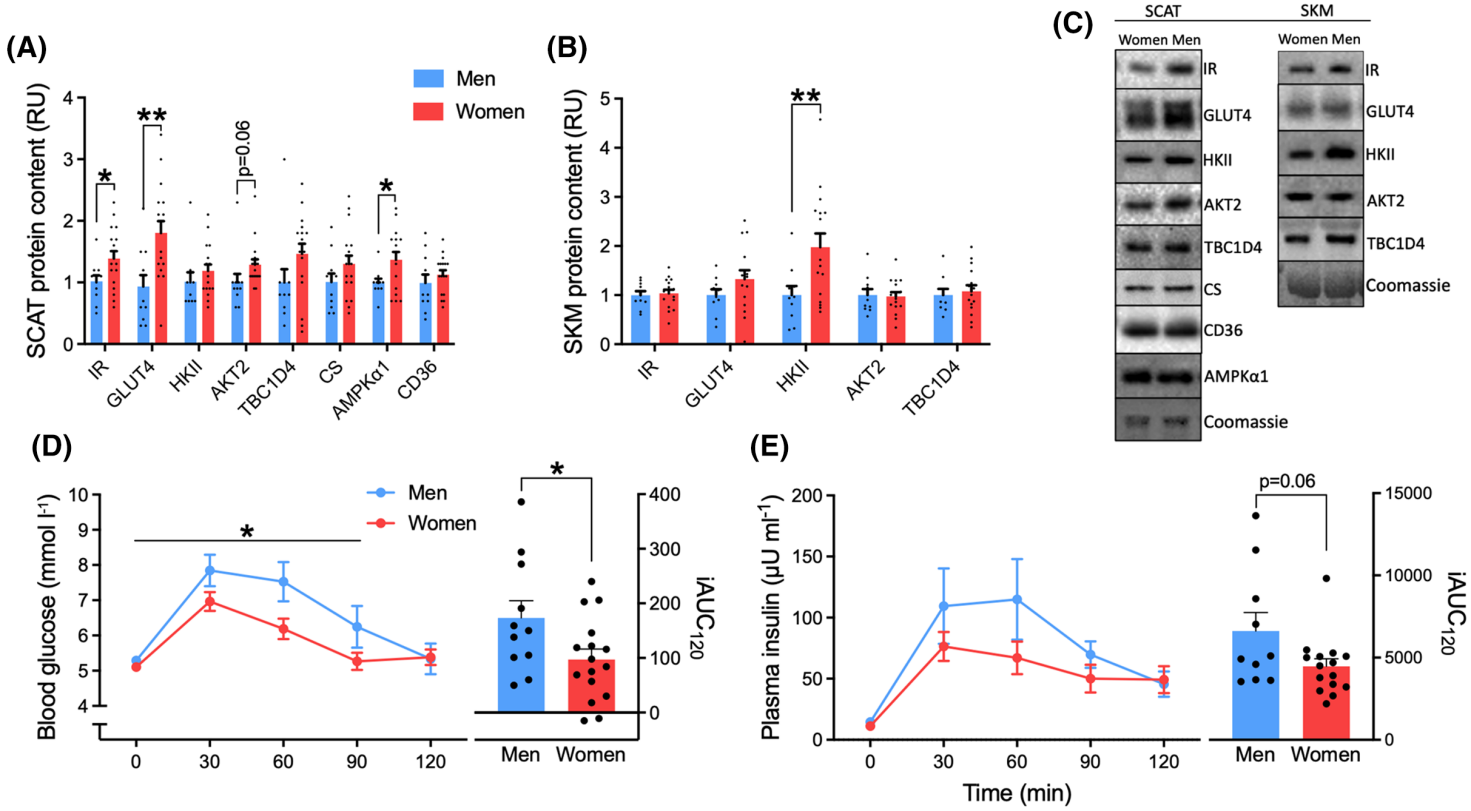
Overweight women have greater capacity for glucose disposal in adipose tissue and greater glucose tolerance than men despite greater adiposity. (A) subcutaneous adipose tissue (SCAT) and (B) skeletal muscle (SKM) glucose metabolic protein contents obtained following an overnight‐fast in premenopausal women and men. (C) representative western blots, data on beta‐actin and total protein are shown in Figure S2. Western blot data are net intensities expressed as relative units (RU) normalized to the men. (D) blood glucose and (E) plasma insulin concentrations and respective incremental areas under the curve (iAUC) during an oral glucose tolerance test. Unpaired *t*‐tests were applied in A and B, and for iAUC in D and E. A two‐way RM ANOVA was applied in D and E with Bonferroni multiple comparisons test. *p < .05, **p < .01 effect or main effect (D) of sex. Data are mean ± SEM. *n* = 16 women, *n* = 10 men.

The women and carefully matched men from the overweight cohort were characterized by a high body fat % in women (46%) and on average 20 kg less lean mass in women than men. The HOMA‐IR index was 2.6 ± 0.6 and 3.0 ± 0.5 in the women and men with overweight (Table 1), and thus higher than in the lean group. Analysis of the glucose metabolic proteins also revealed a greater peripheral capacity for insulin signaling and glucose uptake in women. Hence, in adipose tissue a greater protein content of the insulin receptor (IR) (39%) and GLUT4 (95%) was obtained in women compared with men, and adipose tissue AKT2 protein content was 30% greater (*p* = .06) in female SCAT (Figure 3A), suggesting a higher potential for insulin signaling and translocation of GLUT4 in adipocytes. The greater adipose AMPKα1 protein expression obtained in the lean women was also reproduced in the women with overweight compared with men (Figure 3A). In skeletal muscle, the protein content of HKII was 98% greater in women compared with men (Figure 3B), as also obtained in the lean group, and this could contribute to a greater capacity for glucose uptake. However, in contrast to adipose tissue, skeletal muscle IR, GLUT4, and AKT2 protein expressions were similar between women and men.

The OGTT revealed normal glucose tolerance in the overweight group, with 2 h glucose values of 5.4 ± 0.2 and 5.5 ± 0.5 mmol/L in women and men (Figure 3D). Despite the higher intake of glucose relative to body mass in women (75 g in each sex), lower blood glucose excursion was evident in women during the OGTT, with glucose tolerance being 44% better in women than men, expressed as the incremental area under the curve (iAUC) (Figure 3D). Plasma insulin iAUC tended to be lower in women than in men (*p* = .06) (Figure 3E).

### 3.4 Female mice display greater adipose tissue glucose disposal than male mice

**FIGURE 4:**
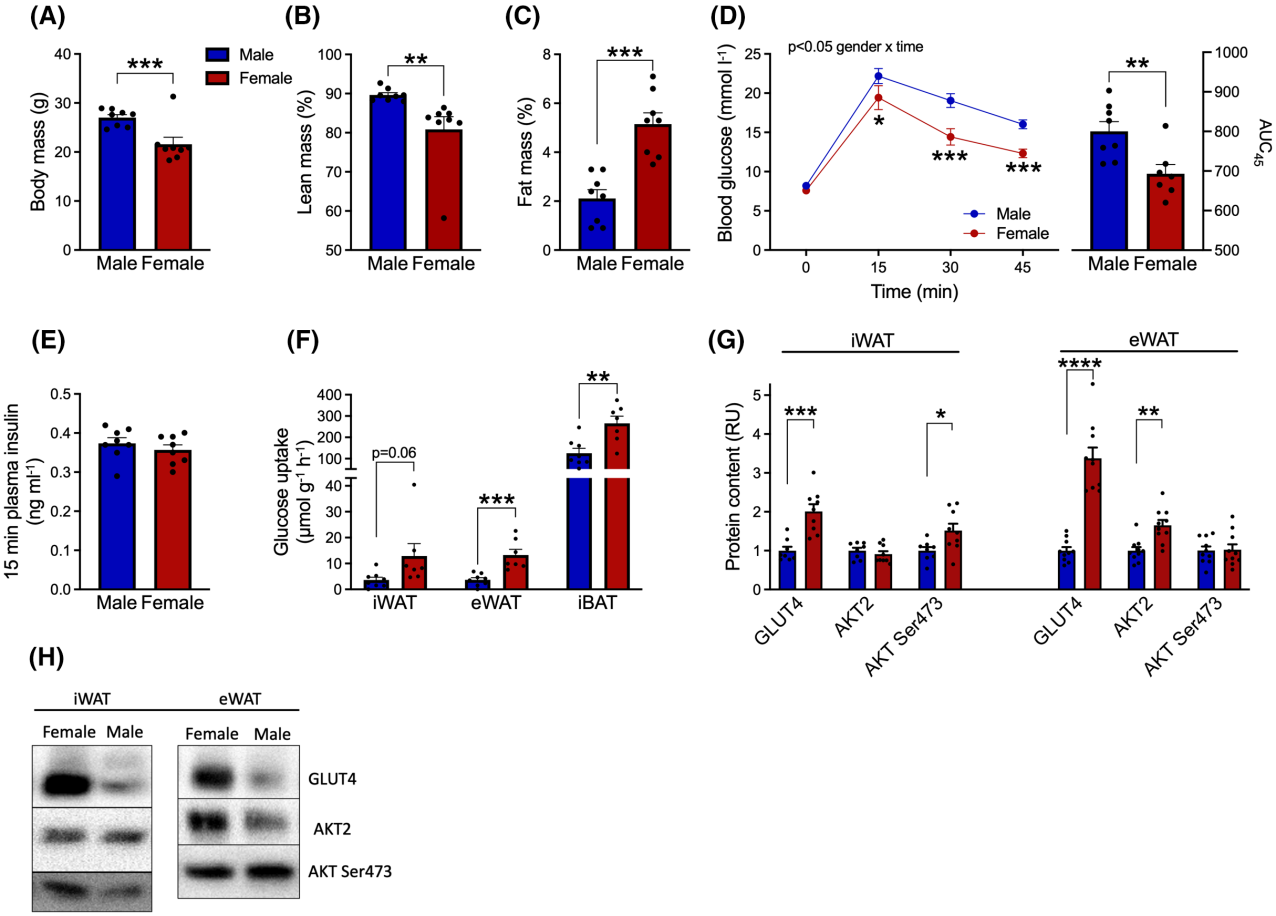
Female mice have greater glucose tolerance and glucose disposal in adipose depots than male mice. (A) body mass, (B) lean mass as % of body mass, (C) fat mass as % of body mass in 14‐ to 16‐week‐old C57BL/6J female and male mice previously fed ad libitum chow diet. Fasting mice were intraperitoneally injected with ^3^H‐2‐deoxyglucose tracer (0.6 μCi/g body mass) plus glucose (2 g/kg body mass), with tissues obtained after 45 min. (D) blood glucose concentrations and area under curve. (E) plasma insulin concentration obtained at 15 min. (F) glucose‐stimulated glucose uptake in adipose tissue depots. (G) glucose metabolic protein contents and phosphorylation in white adipose tissues, obtained 45 min post‐glucose injection. (H) representative western blots. Western blot data are net intensities expressed as relative units (RU) normalized to male mice. An unpaired *t*‐test was applied in A–D (in D, right bar diagram) and E–F. A two‐way RM ANOVA was applied in D (left curve) with Bonferroni multiple comparisons test. *p < .05, **p < .01, ***p < .001, ****p < .0001 female vs. male mice. Data are mean ± SEM. *n* = 8 female mice and *n* = 8 male mice. glucose uptake was assessed in 7 mice, due to scintillation analysis error. Inguinal white adipose tissue (iWAT), epididymal white adipose tissue (eWAT), and intrascapular brown adipose tissue (iBAT).

To directly study sex differences of glucose uptake in different adipose compartments, we traced the glucose uptake in female and male littermate mice. In the chow‐fed mice, body fat percentage was low, but greater in female (5.2 ± 0.4%) than in male mice (2.2 ± 0.3%) (Figure 4A–C). Administration of 2 g glucose (plus trace amounts of ^3^H‐2DG) gave rise to a lower increase in blood glucose in female than in male mice, despite a similar increase in plasma insulin (Figure 4D,E). Tracing of glucose‐induced glucose uptake into adipose tissues revealed that female mice exhibited a markedly greater glucose uptake per gram tissue in both iWAT (254%, *p* = .06), eWAT (260%) and iBAT (111%) compared with male mice (Figure 4F). In iWAT, the protein content of GLUT4 was found to be higher in female mice (201%) as was AKT Ser^473^ phosphorylation (151%) in response to glucose, while in female eWAT, higher GLUT4 (338%) as well as AKT2 (165%) protein was obtained compared with eWAT from male mice (Figure 4G,H).

## 4 DISCUSSION

Here, we show that the subcutaneous adipose tissue in both women with normal weight and women with overweight has a greater potential for glucose uptake in part due to a higher protein expression of IR, AKT2, and GLUT4, and also higher AMPKα1 protein content, when compared to men. In skeletal muscle, the greater capacity was in women evident by a higher content of HKII, PDH‐E1α, and CAV3 protein, and enhanced capillarization and oxidative fiber contribution. The observations in human adipose tissue were consolidated by the ~2‐fold greater glucose uptake across three different adipose tissue depots in female compared with male mice, coinciding with markedly higher GLUT4 and AKT2 protein levels, supporting a significant role of female adipose tissue in whole‐body glucose disposal. Emphasizing the physiological consequence of this superiority in peripheral glucose metabolic protein content and signaling, healthy women with overweight showed markedly greater glucose tolerance than men in response to a similar (75 g) oral glucose load despite the fact that the women with overweight had on average 17 kg less body mass and also less lean mass for distribution of glucose.

### 4.1 Greater potential for glucose uptake in female adipose tissue

In female adipose tissue (abdominal SCAT), we made the novel observation of greater insulin signaling toward glucose transport, as AKT2 protein content and insulin‐induced phosphorylation were greater in lean women compared with men. Higher AKT2 protein was obtained in eWAT of the female mice, while glucose‐induced AKT phosphorylation was higher in female iWAT, which is also a subcutaneous adipose depot. In adipose tissue of women with overweight, we confirmed a greater basal AKT2 expression (*p* = .06) compared with men and did also show greater protein expression of IR and GLUT4, proving a higher capacity for glucose uptake in female adipose tissue independently of increased adiposity. Remarkably, GLUT4 protein content was in the inguinal as well as the epididymal white adipose tissue of the female mice found to be 2‐ to 3‐fold higher than in male mice. Protein content was analyzed in whole adipose tissue homogenates. As adipocytes are known to comprise only ~30%–40% of adipose tissue, we cannot evaluate on any sex‐specific regulation of cells in the stromal vascular fraction. Nevertheless, our observations relate well to previous in vitro findings of greater insulin‐stimulated glucose uptake in primary adipocytes from gluteo‐femoral as well as abdominal SCAT of both premenopausal women with normal weight^14^ and with obesity^15^ compared with men and also provides a molecular explanation for ^18^F‐FDG‐PET findings, showing that premenopausal lean women had markedly greater glucose uptake per unit of abdominal SCAT compared with lean men during moderate insulin infusion.^16^ Our findings also add a mechanistic explanation to a study in 216 men and 110 women using ^18^F‐FDG‐PET showing that women had 20% greater glucose uptake of abdominal SCAT than men at high insulin infusion (40 mU m^−2^ min^−1^), and interestingly that SCAT glucose uptake correlated well with whole‐body glucose uptake (*r* = .621 in women and less so in men, *r* = .360).^32^ Protein expression was only evaluated in abdominal SCAT in the present study, but the in vitro observations of the presence of greater glucose disposal in female adipocytes obtained from gluteo‐femoral biopsies^14^ suggest that the greater glucose metabolic protein apparatus in women could also extend to other subcutaneous adipose depots. This suggest that in the obese state, characterized by high adipose tissue mass, a more pronounced sex‐dependent phenotype in glucose tolerance may be evident. The marked sex difference in glucose tolerance between women and men with obesity was evident even though the women had a body fat percentage of 46% and the men had 44% more lean mass, pointing to the contribution of adipose tissue to glucose disposal. Sex differences in the incretin hormone response, splanchnic glucose disposal, or peripheral glucose effectiveness cannot be excluded, aspects which await to be further investigated.

### 4.2 Greater glucose disposal per mass of skeletal muscle in lean women

We^10^, ^11^ and others^7^ have previously shown that insulin‐stimulated leg glucose disposal is greater in premenopausal women than in men when expressed relative to the lean mass of the leg, as a way of expressing the glucose disposal per mass of skeletal muscle. The greater protein expression for glucose uptake in female skeletal muscle could relate to the greater area contribution of the more insulin‐sensitive type I fibers,^10^ and the percentage of type I fibers has been shown to highly correlate with glucose uptake in human muscle strips.^33^ The greater HKII, PDH‐E1α, and CAV3 protein expression in female skeletal muscle indicates superior capacity for both glucose uptake (via increased glucose gradient) and glucose oxidation, respectively, and the pronounced sex difference in HKII protein expression was maintained in the group of individuals with overweight. Concomitantly, a greater capacity for FA uptake in female skeletal muscle was evident from the greater level of the FA transport protein CD36. Female skeletal muscle thus seems to possess a greater capacity for disposal of both circulating glucose and fatty acid substrates.

While the present findings at the protein level provide an explanation for the sexual dimorphism in substrate metabolism shown in the present study, the underlying sex‐specific regulation was not investigated. Sex‐specific regulation plays an important role in the tissue transcriptome, and adipose tissue and skeletal muscle are among the 5 tissues with the largest number of differentially expressed genes by sex in humans.^34^ Gonadal hormones, including estrogen and androgens, and hormone‐related transcription factors may be the major regulators of sex‐dependent gene expression, and several studies have shown the importance of estrogen, estrogen receptors, and estrogen‐responsive transcription factors in regulation of glucose metabolic genes and insulin action in adipose tissue and skeletal muscle.^35^ For example, estrogen receptor activation in adipocytes increases insulin action at the level of AKT and GLUT4.^36^

### 4.3 Basal glucose and fatty acid availability

A lower postabsorptive blood glucose was obtained in the lean women compared with men, as supported by some,^5^, ^37^ but not all studies.^11^, ^38^, ^39^ The lower blood glucose was in the basal state associated with a lower basal endogenous glucose production. The lower blood glucose in the lean women was found concomitant with a 52% greater postabsorptive plasma FA concentration compared with men, in agreement with previous studies.^10^, ^40^, ^41^, ^42^ Tracer studies have confirmed a greater fasting plasma FA rate of appearance in women than in men,^43^, ^44^, ^45^ which related to a greater amount of adipose tissue in women rather than a greater lipolytic activity per mass unit of adipose tissue,^44^ supported by the similar protein content and basal phosphorylation levels of the lipolytic enzymes ATGL and HSL in SCAT of the lean women and men. The greater circulating FA levels in women were, in concert with the greater protein level of CD36, associated with a greater reliance on whole‐body FA oxidation compared with men, both during basal and insulin‐stimulated conditions. Still, the relative shift to increased glucose oxidation when insulin was infused was similar between sexes, as was glucose infusion rate, proving the impact of sex on the interaction between circulating FAs and insulin action.^11^

## 5 LIMITATIONS

The present study only contained participants that would be classified as white with European ancestry, and it cannot be excluded that sex differences could be different in populations of another race. Also, while we in the study only investigated SCAT, it would be important to also study sex differences of other adipose tissue depots, particularly as it was recently shown by deep proteomic profiling how much various white adipose depots differed in protein expression of metabolic enzymes likely pointing to different functions of each depot.^46^ Lastly, it is worth noticing that the present study only investigated age‐matched young premenopausal women and men of 20–35 years of age and either normal weight or with overweight, and it hence remains to be investigated whether elderly women and men and/or with more severe obesity would exhibit similar sex differences. Particularly, it seems plausible that many physiological differences might disappear in postmenopausal women compared with men.

## 6 CONCLUSION

The superior molecular imprint for glucose disposal in both female adipose tissue and skeletal muscle, evident by a higher expression and activation of several proteins handling glucose uptake, gives rise to a similar insulin‐stimulated glucose disposal and better whole‐body glucose tolerance in women than men. The lower ratio of lean mass to fat mass in women thus seems to be compensated for by a greater potential for glucose uptake in female adipose tissue, underscoring that skeletal muscle is not the only tissue displaying better insulin sensitivity in women compared with men. The present molecular observations indicate an overall greater capacity of women to dispose circulating glucose as well as FA substrates in peripheral tissues, which may have had evolutionary benefits for women in terms of substrate storage. Future studies should investigate which specific exercise training interventions and dietary strategies that most efficiently can affect glucose and lipid metabolic machinery in women and men separately, and also try to elucidate the molecular mechanisms underlying differences in metabolic protein machinery between women and men.

## AUTHOR CONTRIBUTIONS

Trine S. Nicolaisen, Anne‐Marie Lundsgaard, Bente Kiens, Erik A. Richter, Kim A. Sjøberg, Andreas M. Fritzen, and Christian S. Carl designed the studies and/or carried out the experiments. Anne‐Marie Lundsgaard, Trine S. Nicolaisen, Andreas M. Fritzen, Christian S. Carl, and Kim A. Sjøberg contributed to the results. Anne‐Marie Lundsgaard, Trine S. Nicolaisen, Andreas M. Fritzen, and Bente Kiens wrote the manuscript. All authors contributed to the manuscript and approved the final version.

## FUNDING INFORMATION

B.K. and E.A.R were funded by The University of Copenhagen Excellence Program for Interdisciplinary Research (2016): “Physical activity and Nutrition for Improvement of Health” and the Independent Research Fund Denmark – Medical Sciences (grant: 4183‐00249). A‐M.L. and A.M.F. were supported by a postdoctoral research grant from the Danish Diabetes Academy, funded by the Novo Nordisk Foundation, grant number NNF17SA0031406. Furthermore, A.M.F. was supported by the Novo Nordisk Foundation, grant number NNF22OC0074110. K.A.S was supported by a postdoctoral research grant from the Independent Research Fund Denmark – Medical Sciences, grant number 4092‐00309.

## DISCLOSURES

A.M.L. is employed at Novo Nordisk A/S. All other authors declare that they have no competing interests.

## Supporting information


Figure S1.

Figure S2.

Table S1.

Table S2.

## Data Availability

All raw data that support the findings of this study are available in the supplementary material of this article.
