# Supplementary material for: Greater molecular potential for glucose metabolism in adipose tissue and skeletal muscle of women compared with men
Source: FASEB J. 2024 Jul 31;38(15):e23845. doi: 10.1096/fj.202302377R (PMC11607633; doi:10.1096/fj.202302377R)

Full unedited blots – overweight group

IR

Subcutaneous adipose tissue (fig. 3)

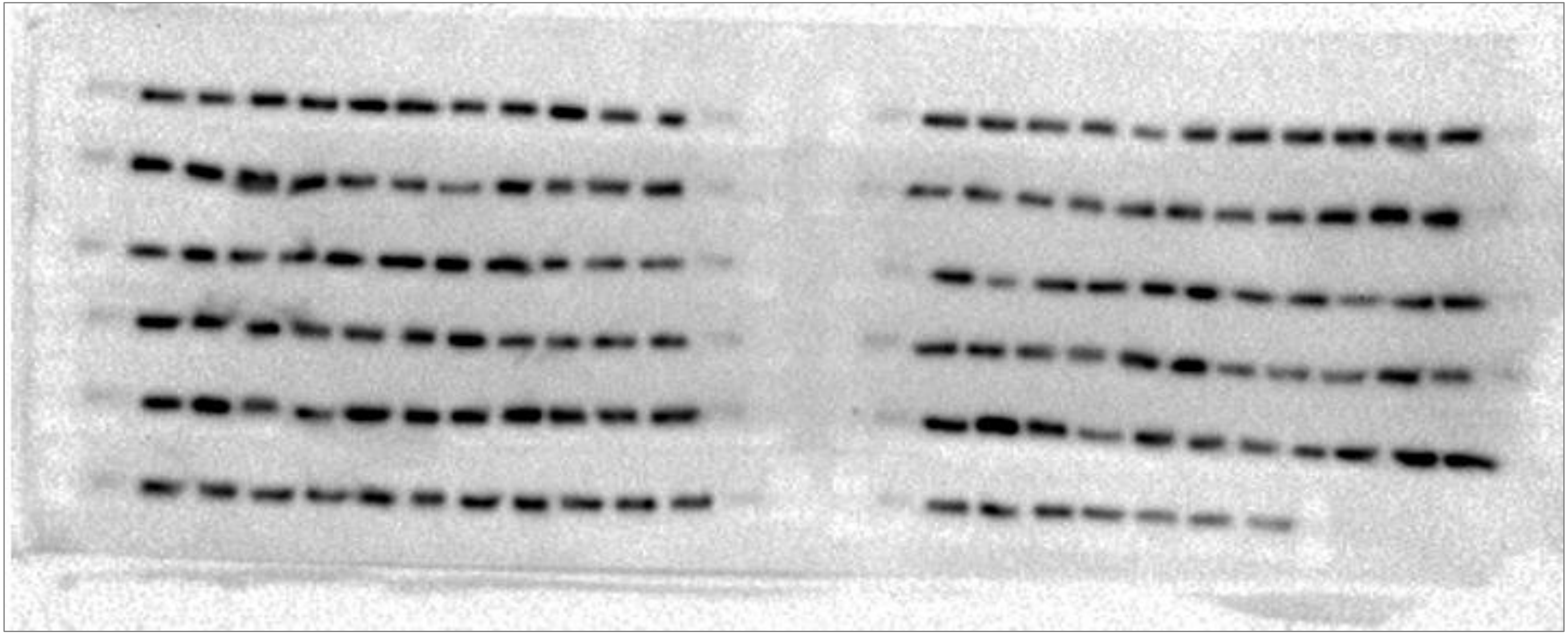

Full unedited blots – overweight group

GLUT4

Subcutaneous adipose tissue (fig. 3)

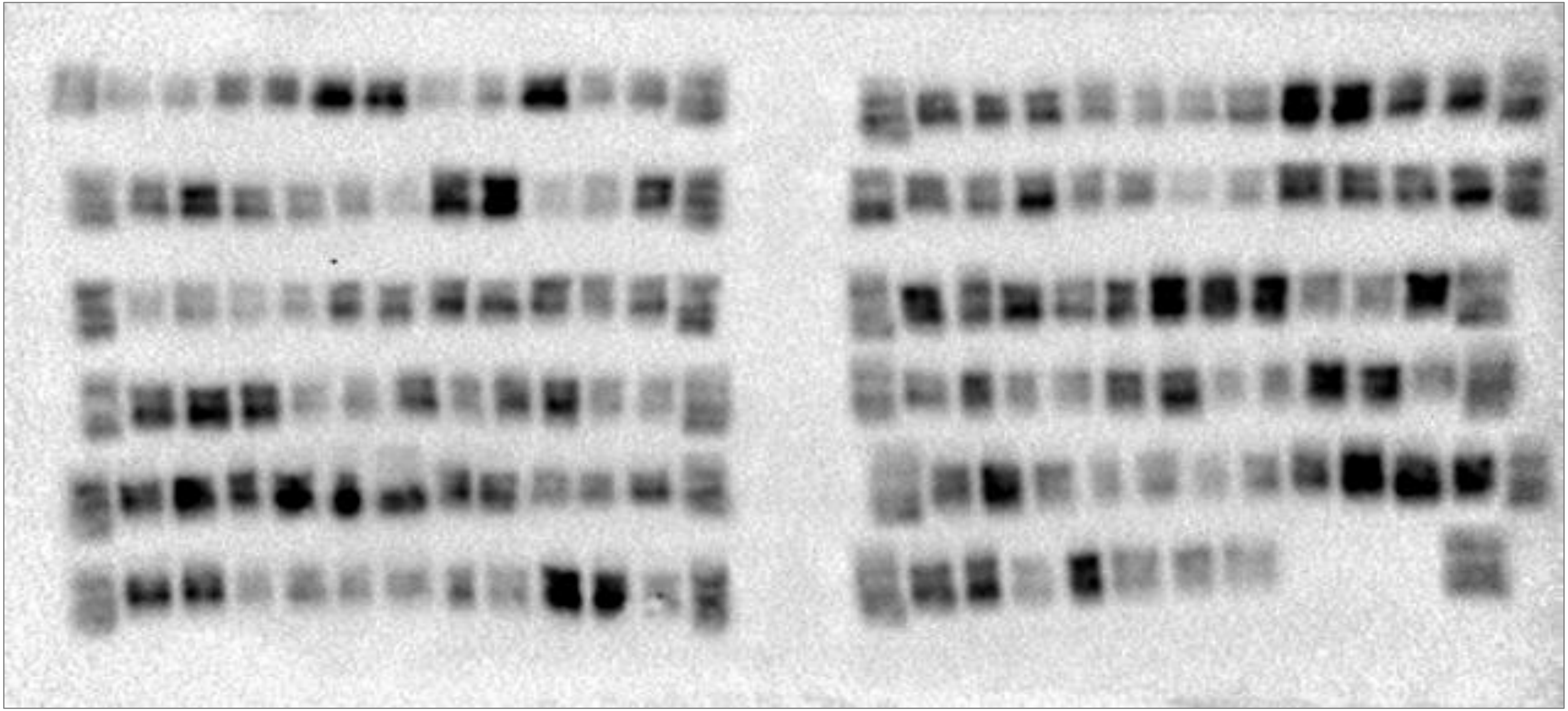

HKII

Full unedited blots – overweight group

Subcutaneous adipose tissue (fig. 3)

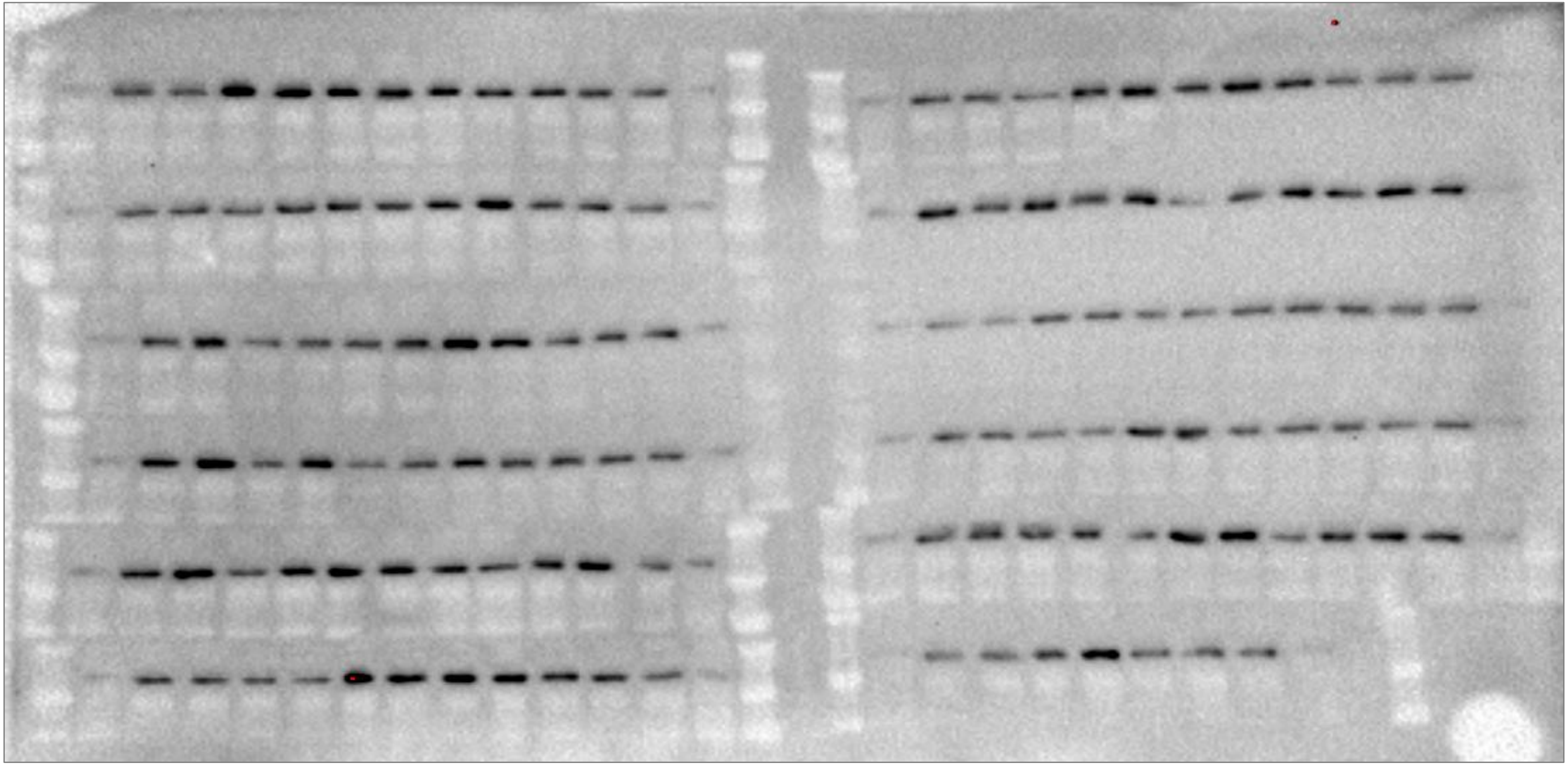

Full unedited blots – overweight group

AKT2

Subcutaneous adipose tissue (fig. 3)

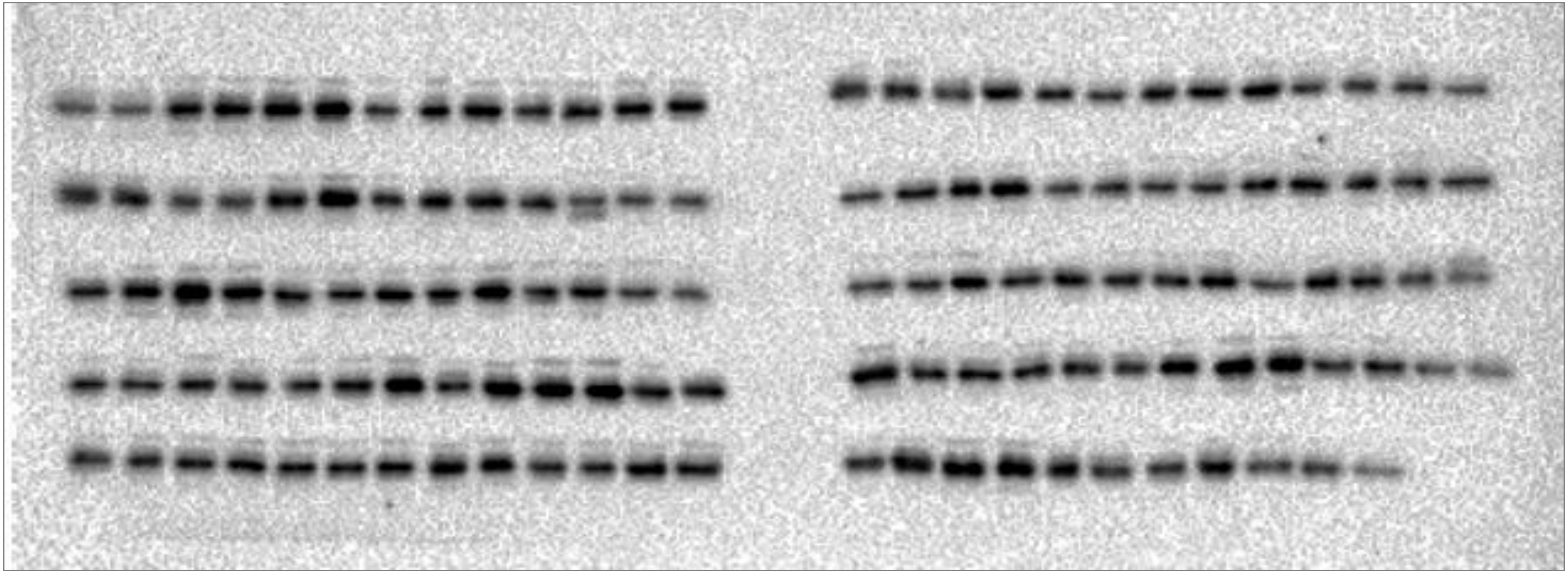

Full unedited blots – overweight group

TBC1D4

Subcutaneous adipose tissue (fig. 3)

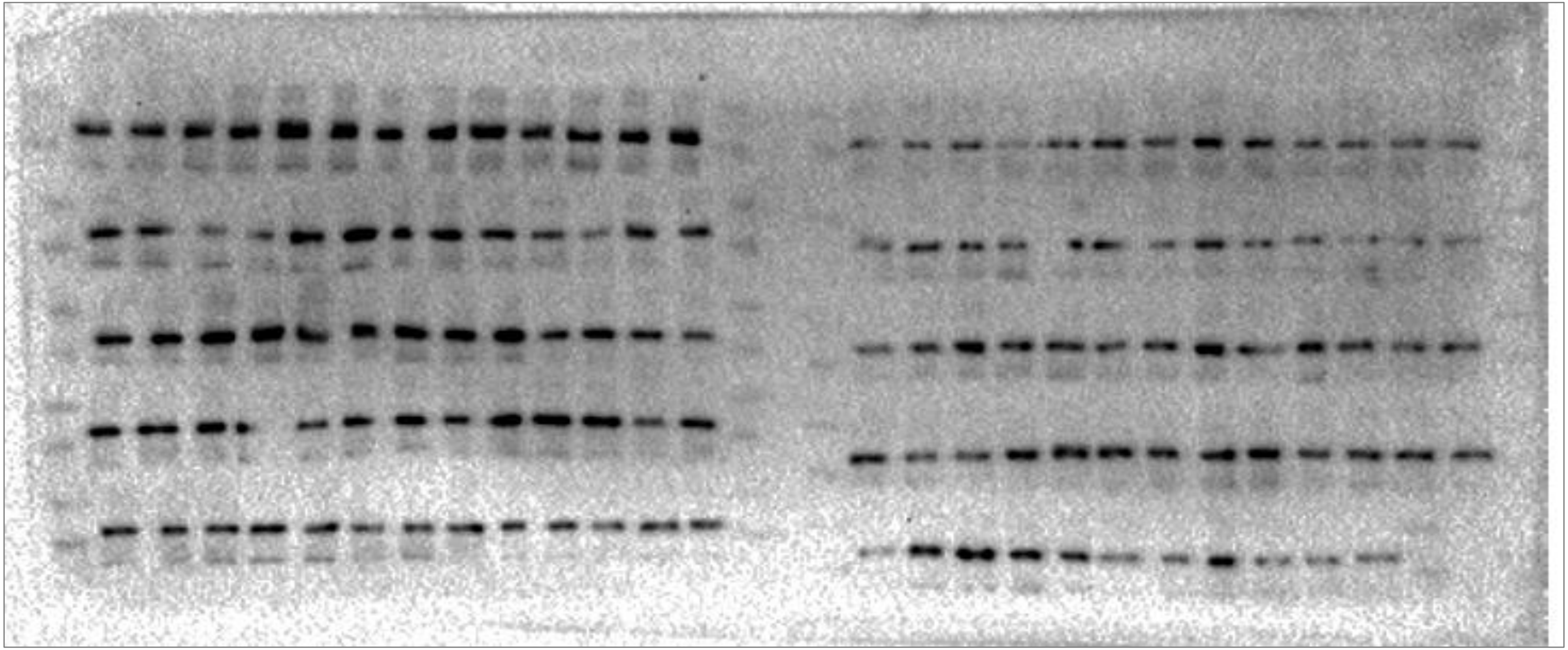

CS

Full unedited blots – overweight group

Subcutaneous adipose tissue (fig. 3)

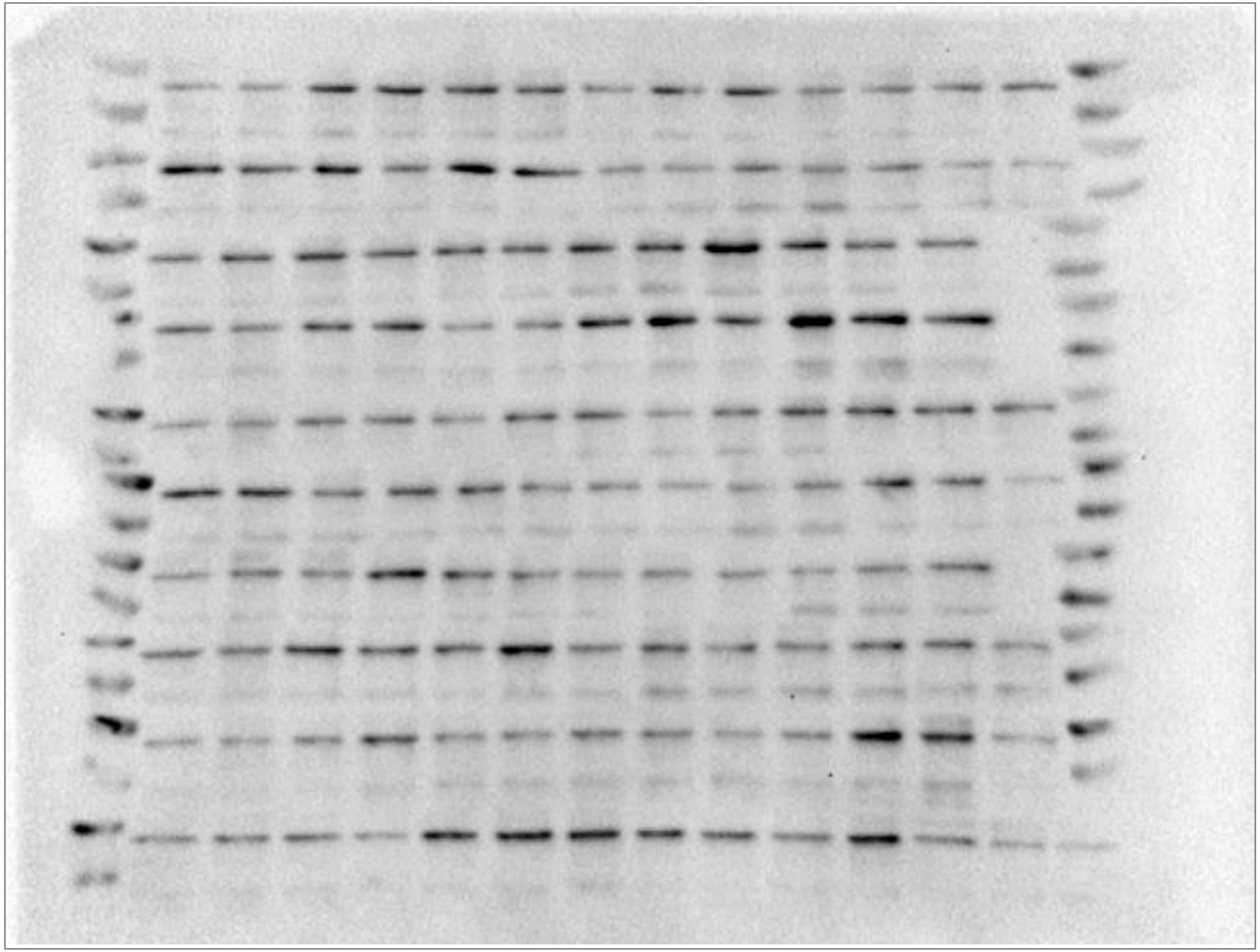

Full unedited blots – overweight group

CD36

Subcutaneous adipose tissue (fig. 3)

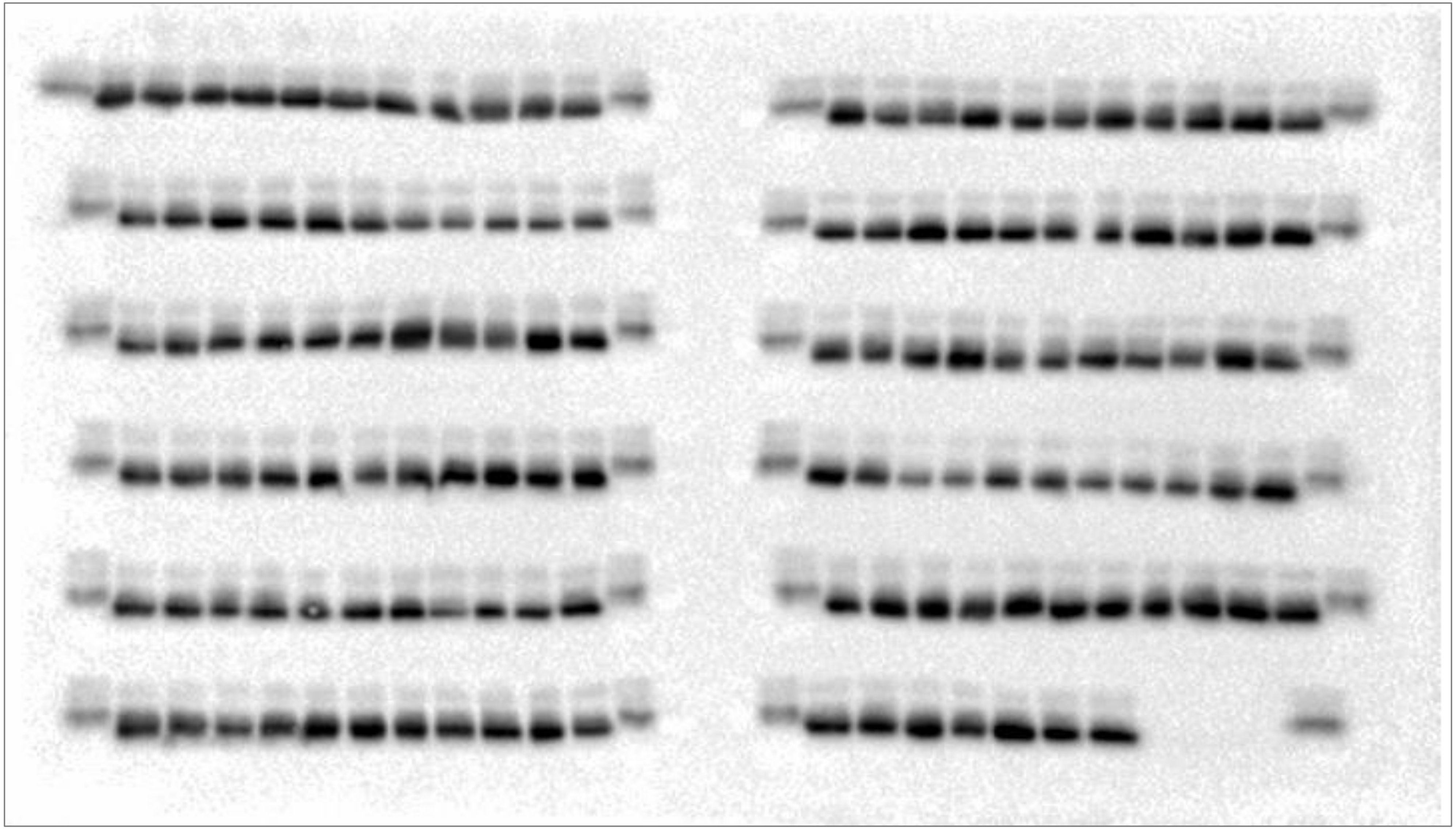

Full unedited blots – overweight group

AMPKa1

Subcutaneous adipose tissue (fig. 3)

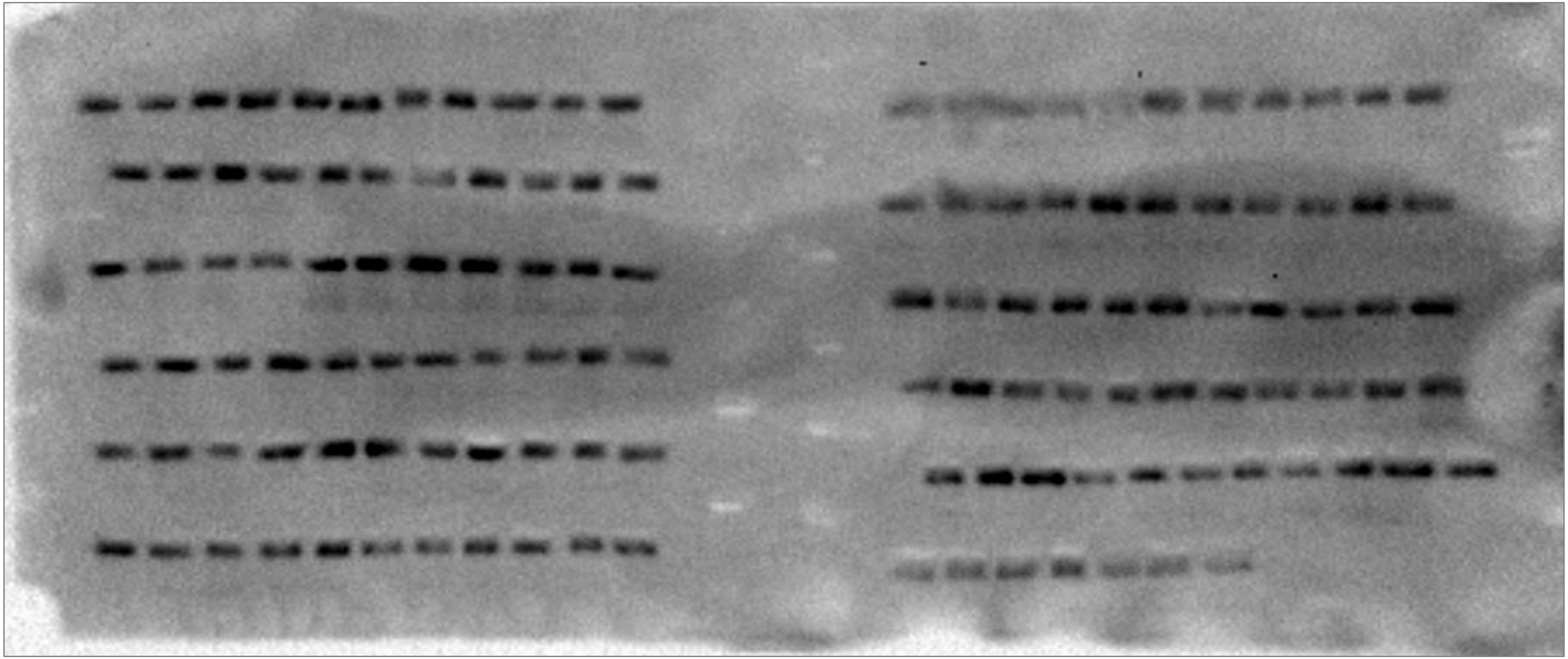

Full unedited blots – overweight group

IR

Skeletal muscle (fig. 3)

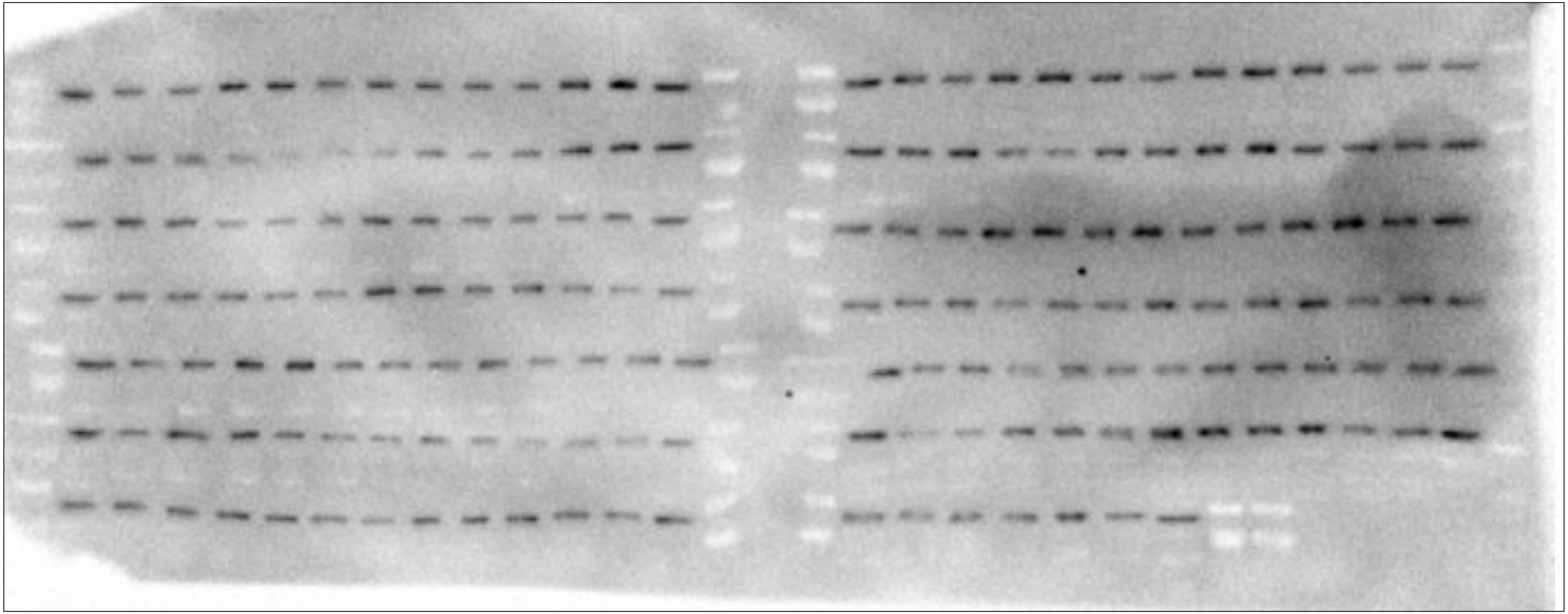

GLUT4

Full unedited blots – overweight group

Skeletal muscle (fig. 3)

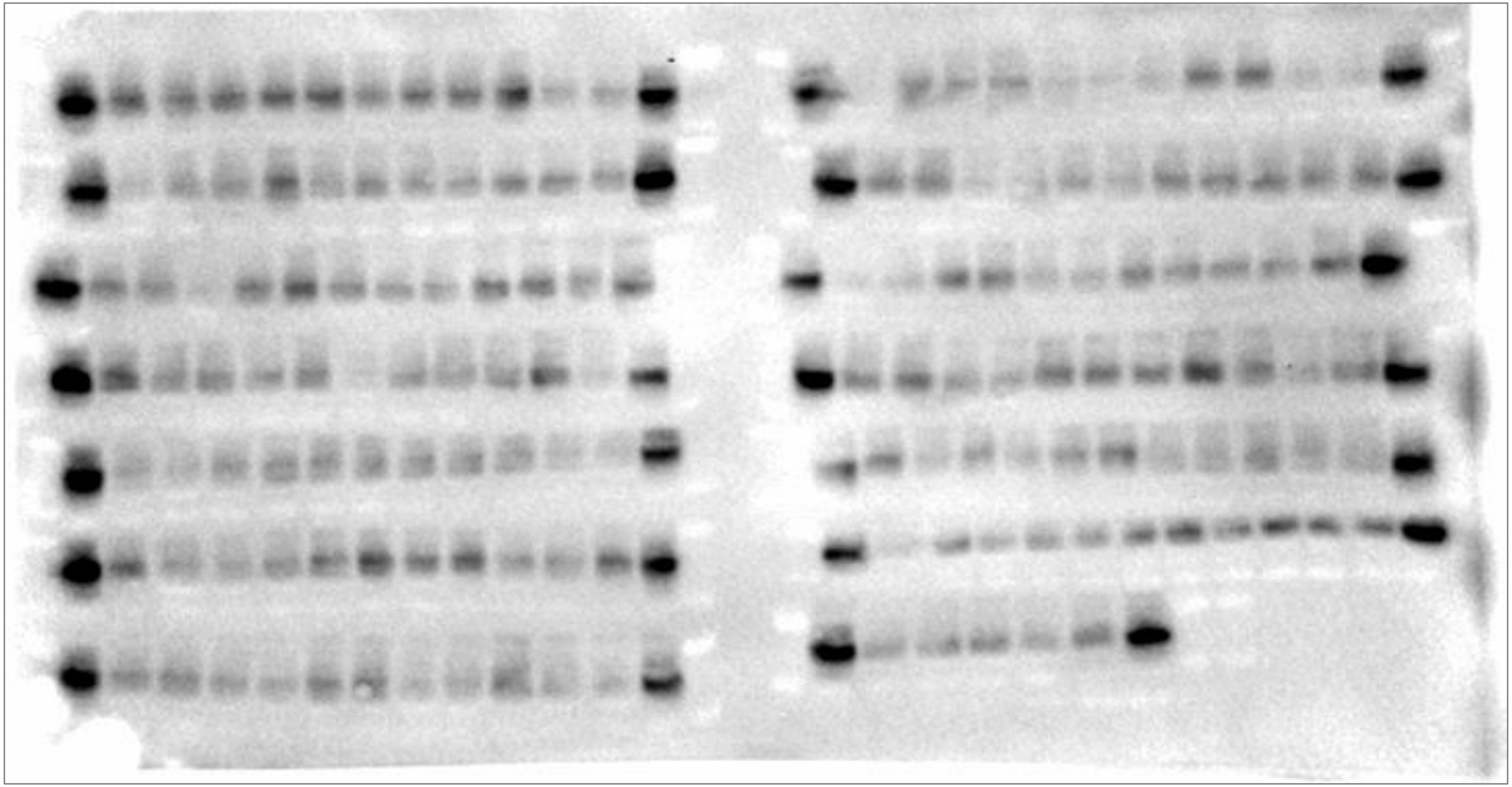

Full unedited blots – overweight group

HK2

Skeletal muscle (fig. 3)

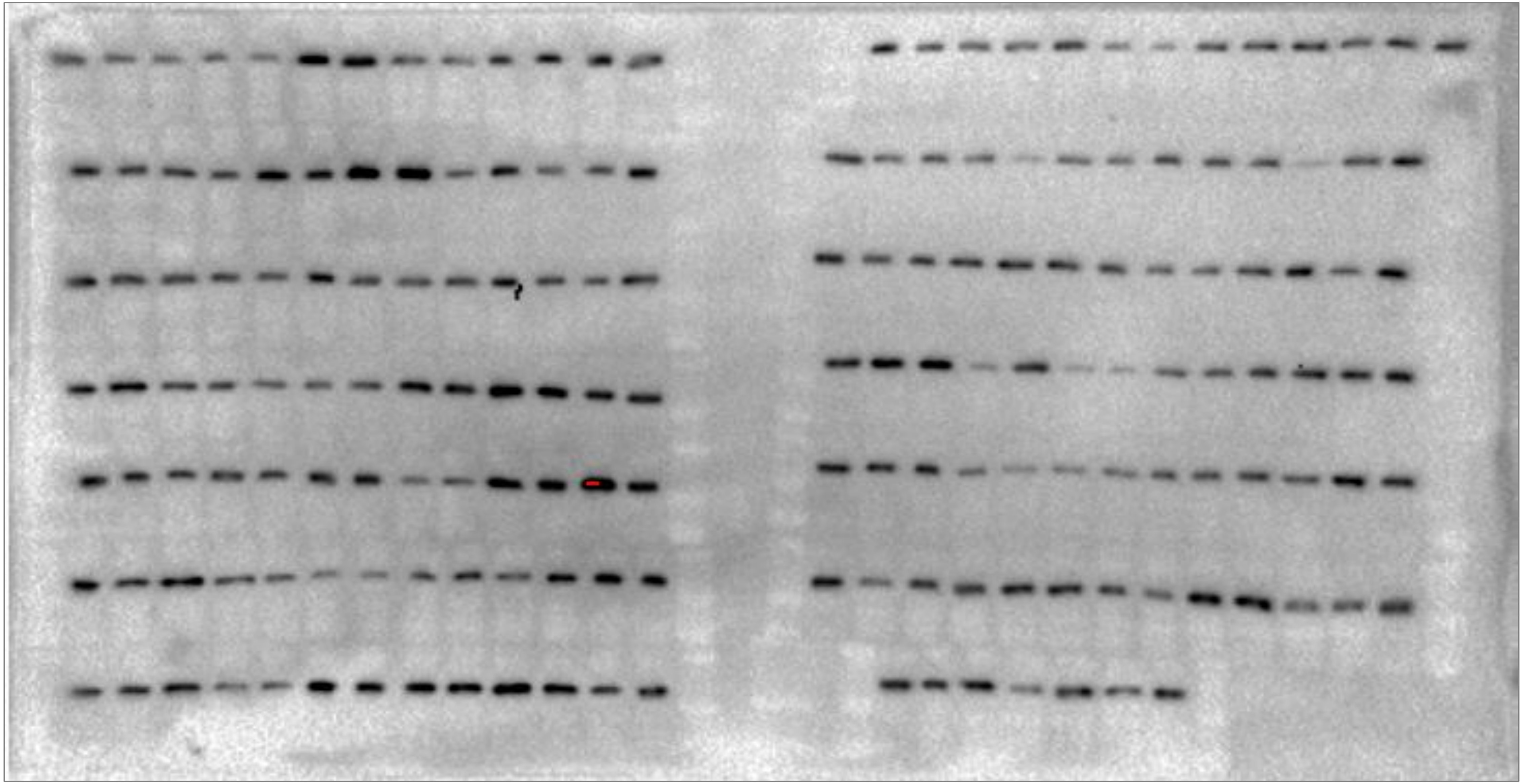

Full unedited blots – overweight group

AKT2

Skeletal muscle (fig. 3)

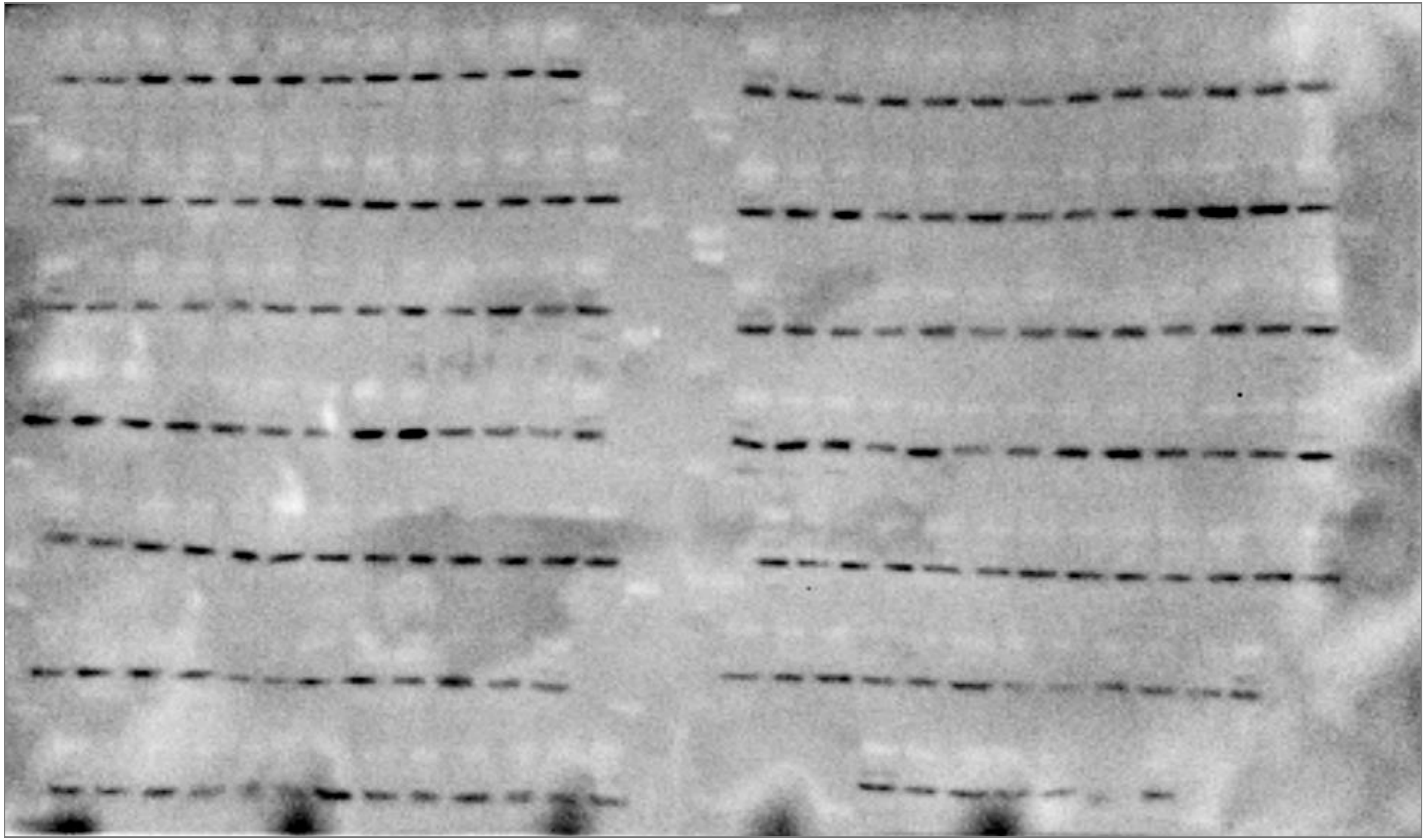

Full unedited blots – overweight group

TBC1D4

Skeletal muscle (fig. 3)

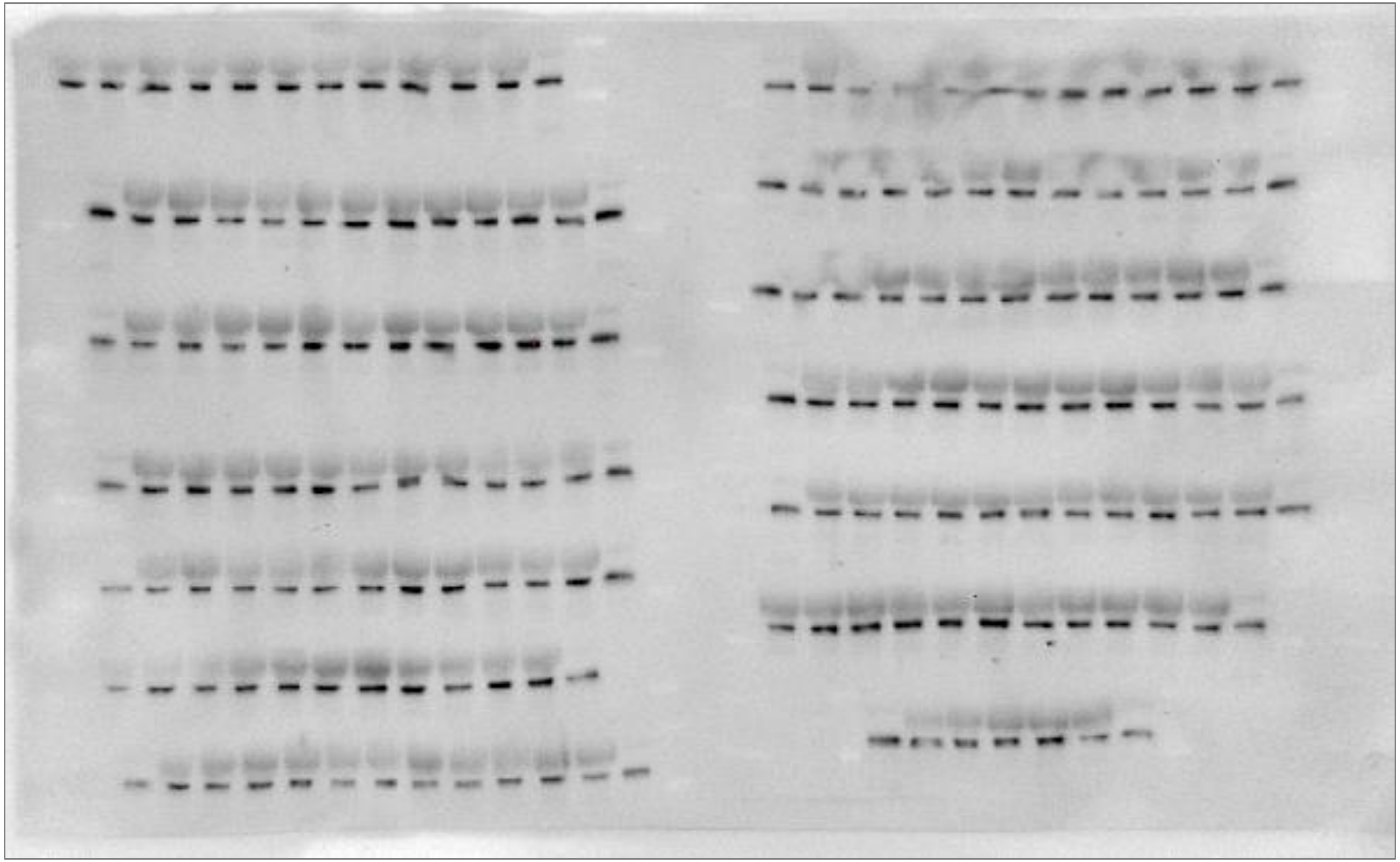

Full unedited blots – lean group

IR

Subcutaneous adipose tissue (fig. 1)

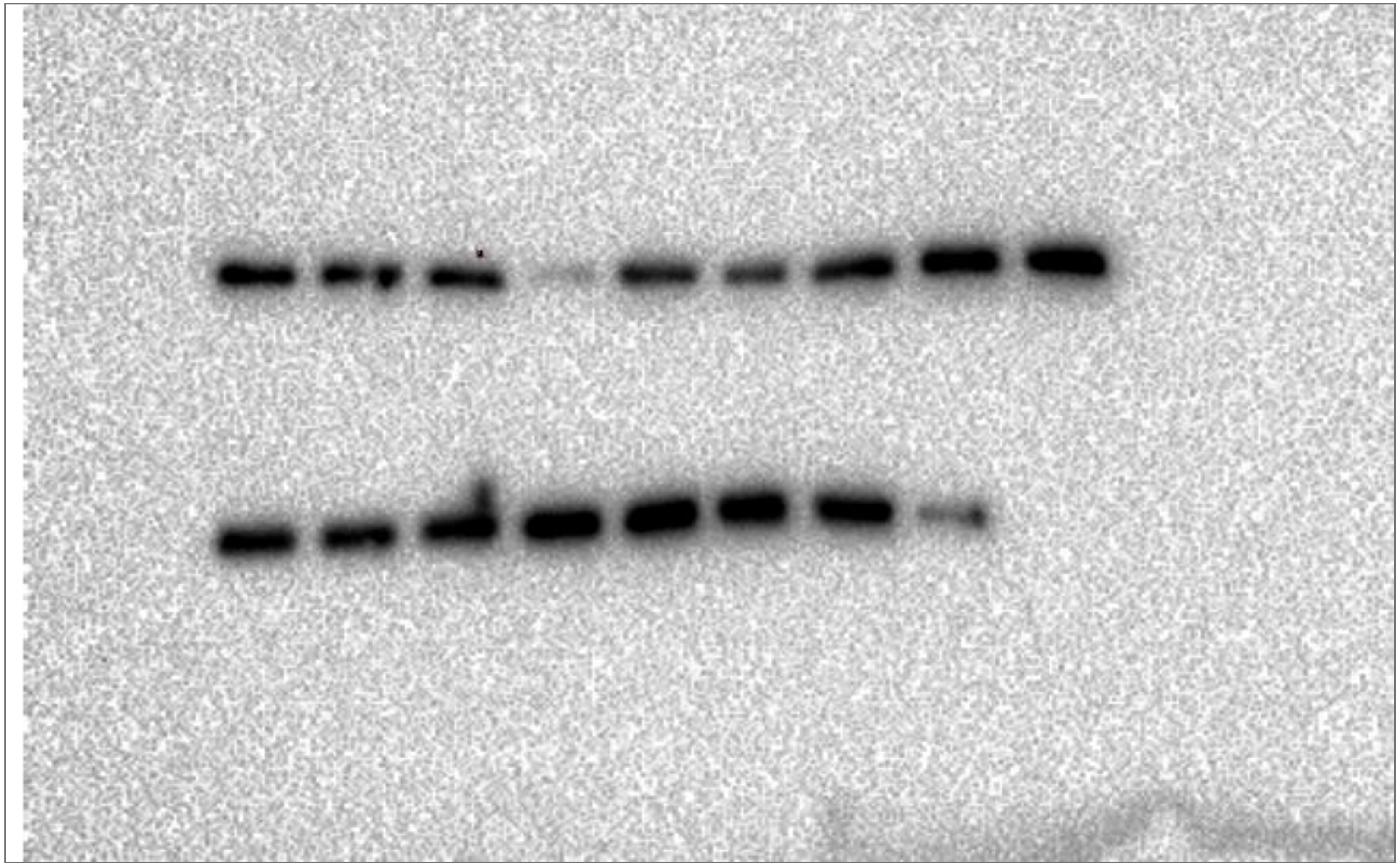

GLUT4

Full unedited blots – lean group

Subcutaneous adipose tissue (fig. 1)

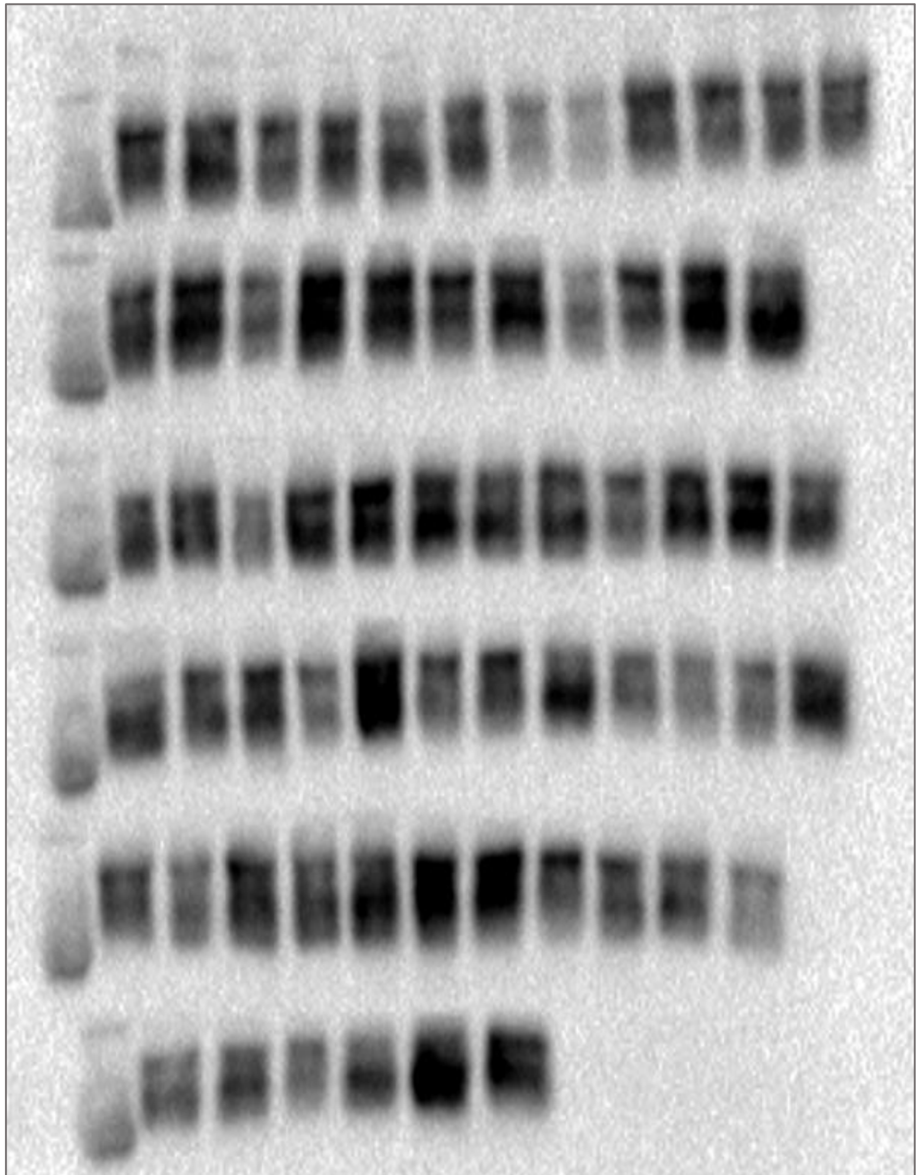

HKII

Full unedited blots – lean group

Subcutaneous adipose tissue (fig. 1)

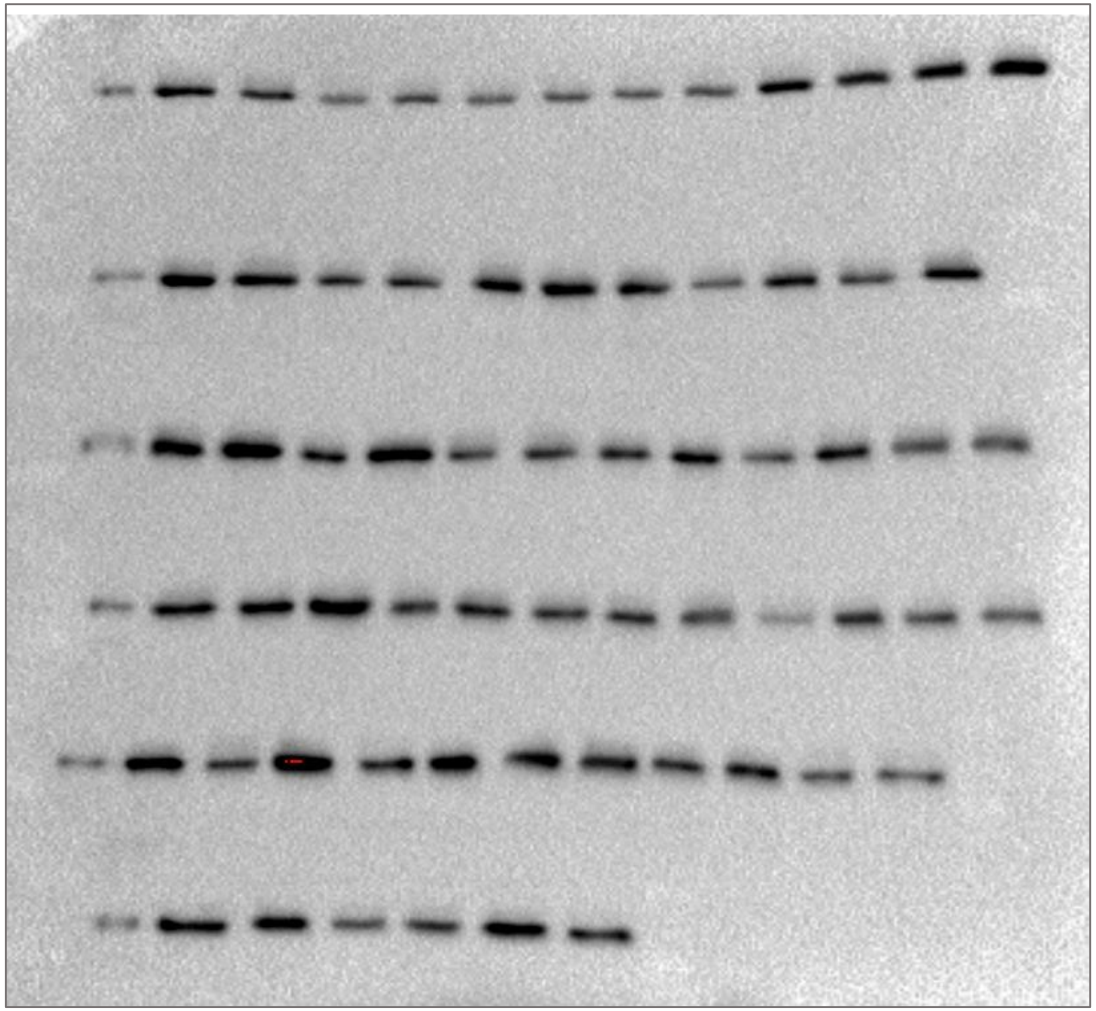

Full unedited blots – lean group

TBC1D4

Subcutaneous adipose tissue (fig. 1)

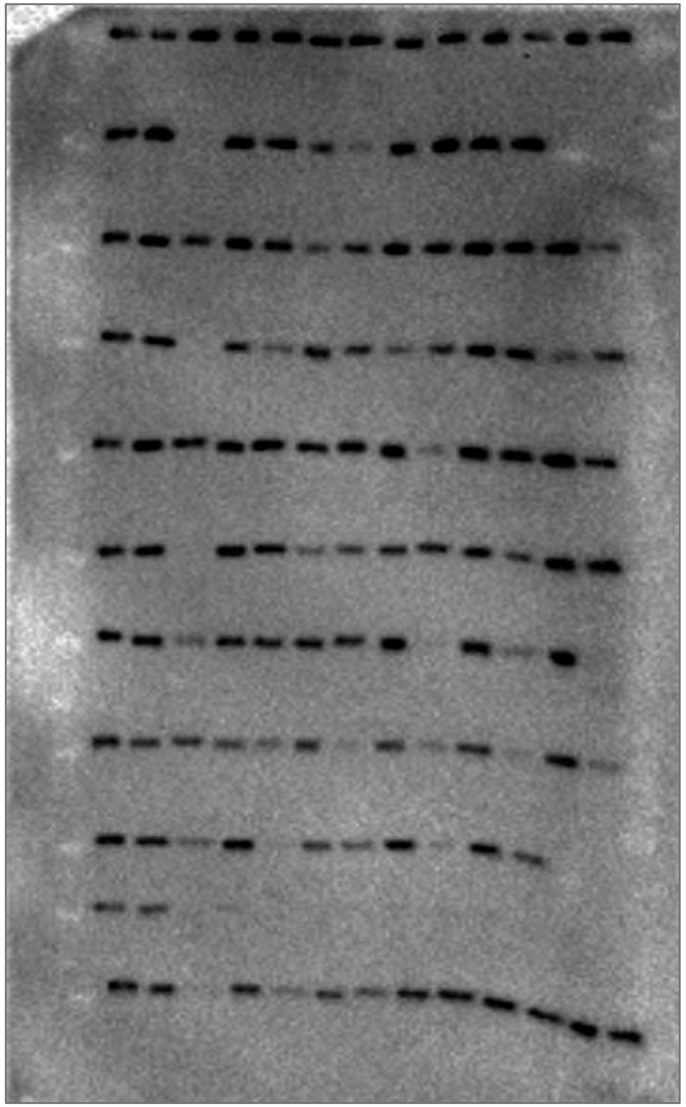

CS

Full unedited blots – lean group

Subcutaneous adipose tissue (fig. 1)

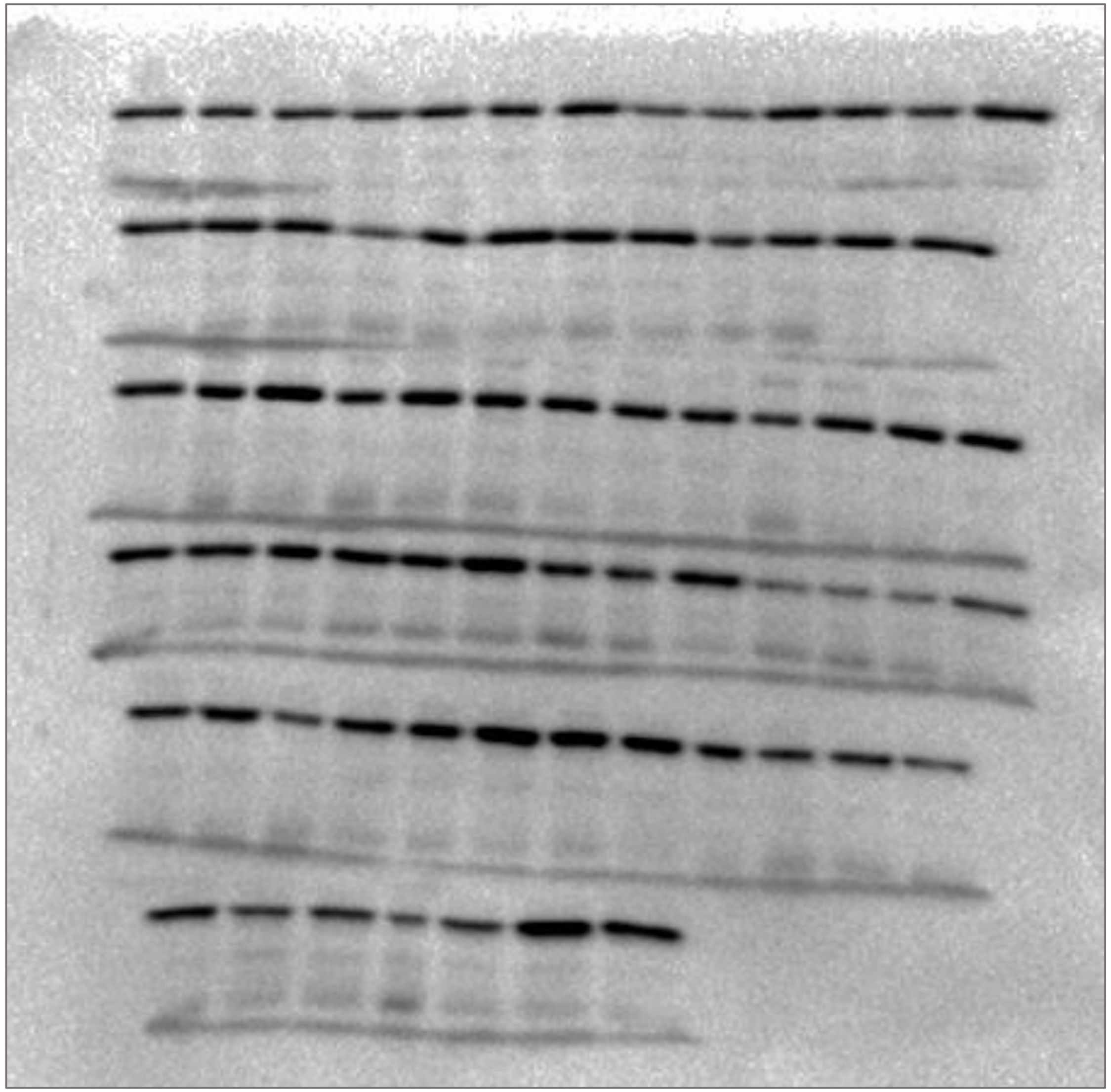

pAMPK Thr172

Full unedited blots – lean group

Subcutaneous adipose tissue (fig. 1)

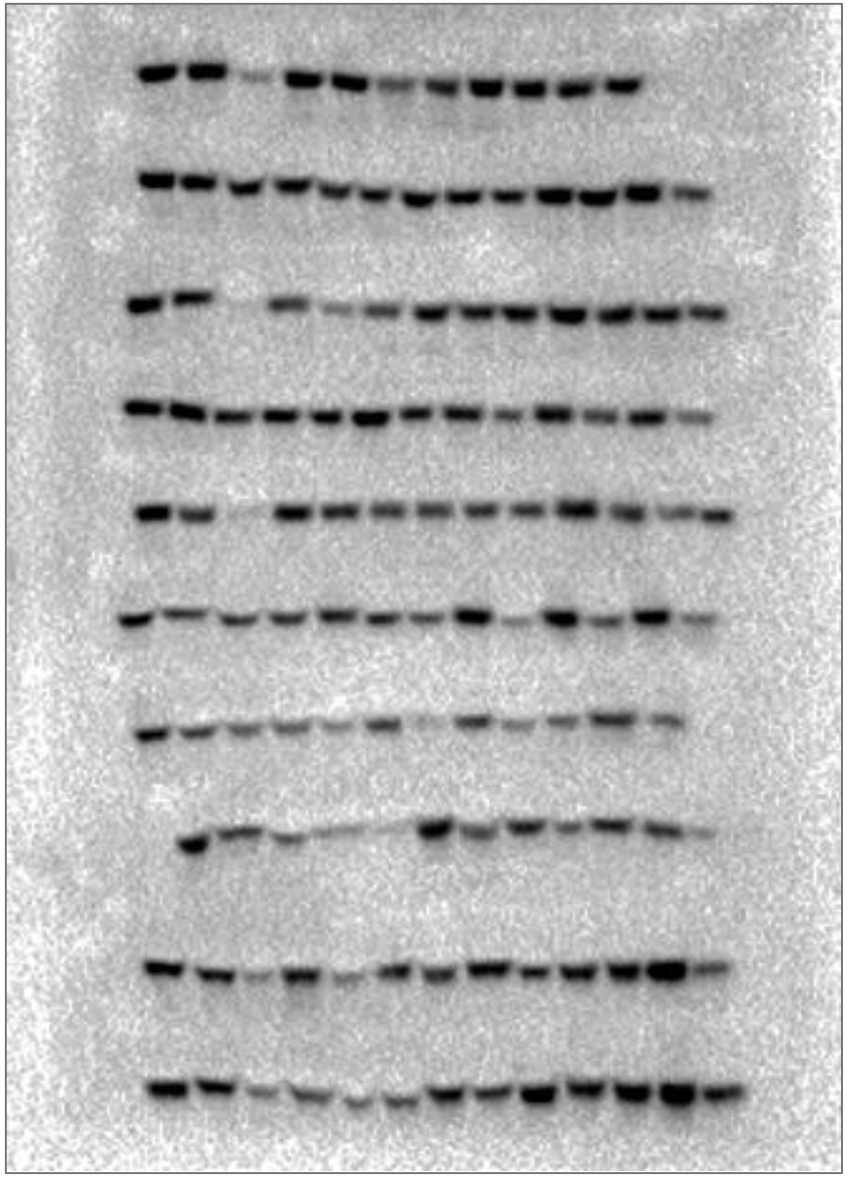

AMPKα1

Full unedited blots – lean group

Subcutaneous adipose tissue (fig. 1)

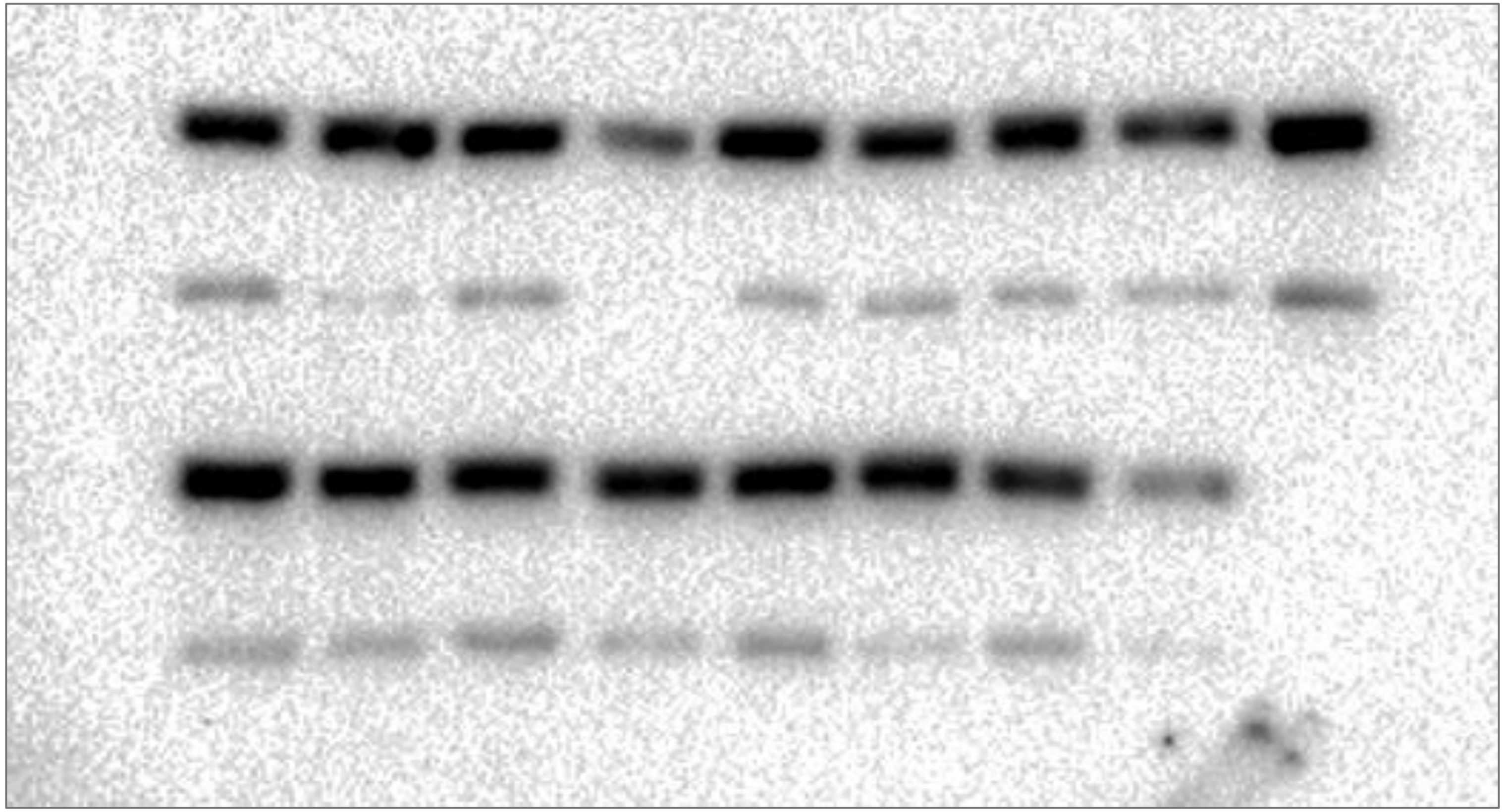

Full unedited blots – overweight group

ACC

Subcutaneous adipose tissue (fig. 1)

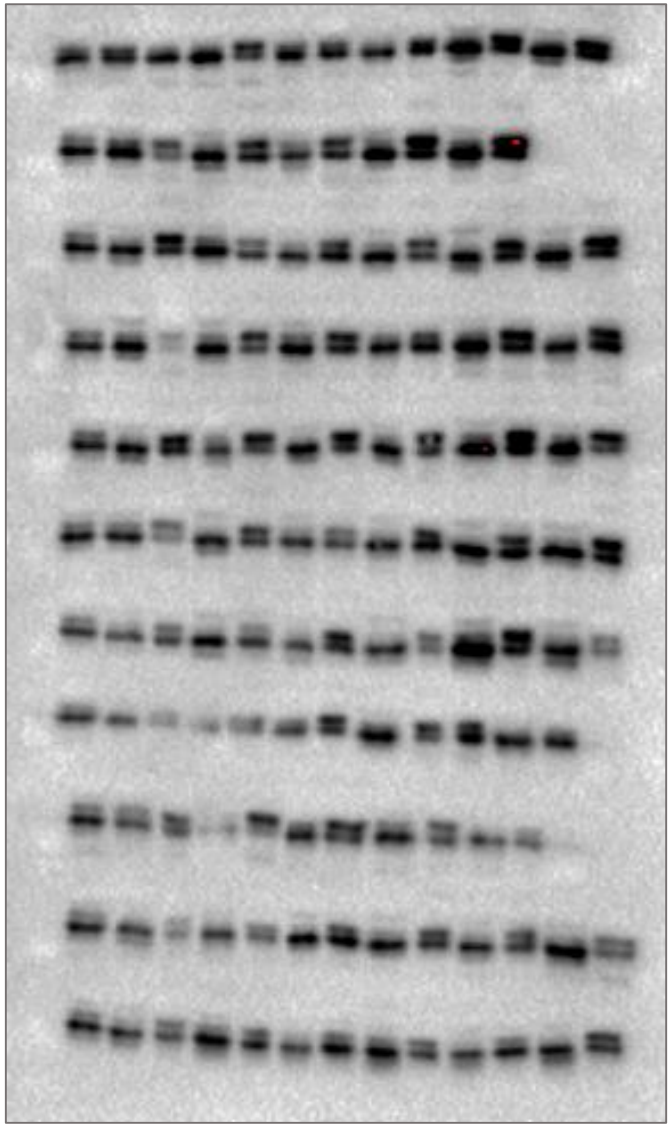

CD36

Full unedited blots – overweight group

Subcutaneous adipose tissue (fig. 1)

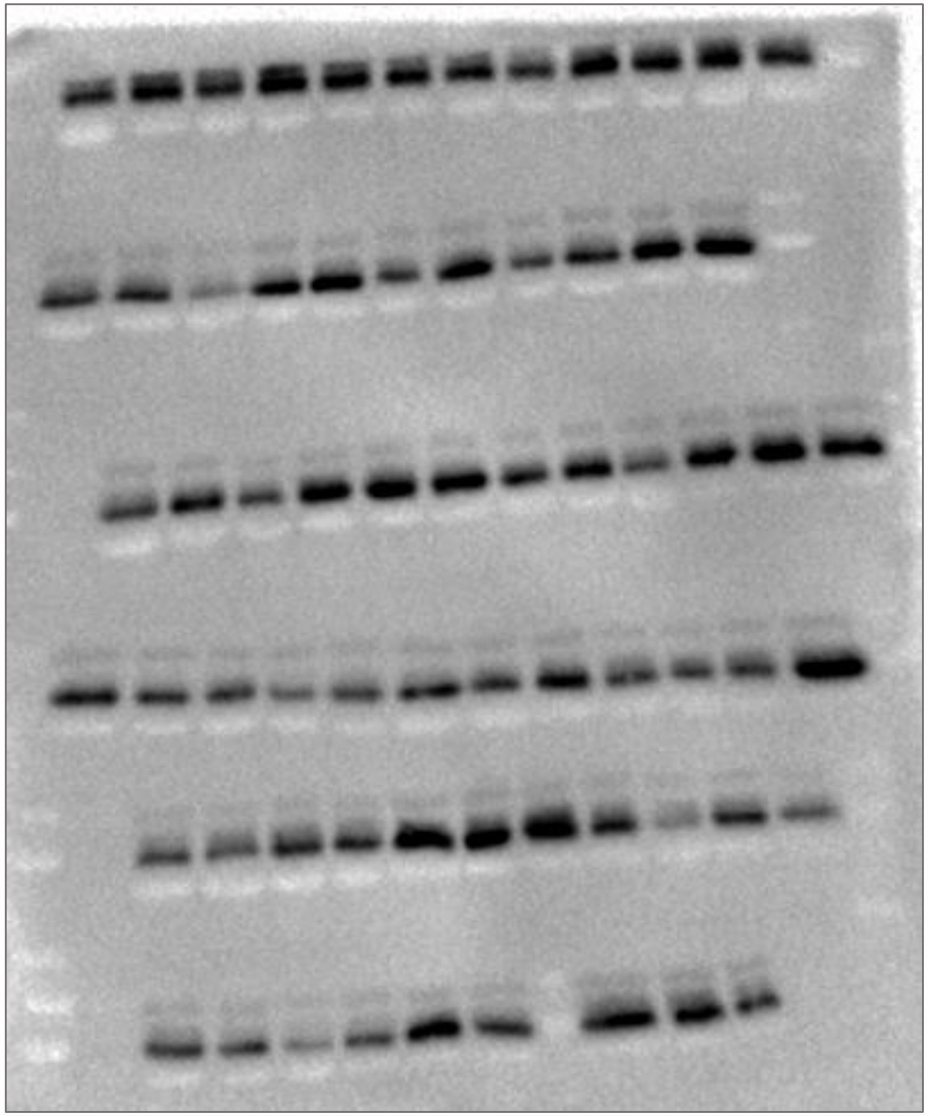

ATGL

Full unedited blots – overweight group

Subcutaneous adipose tissue (fig. 1)

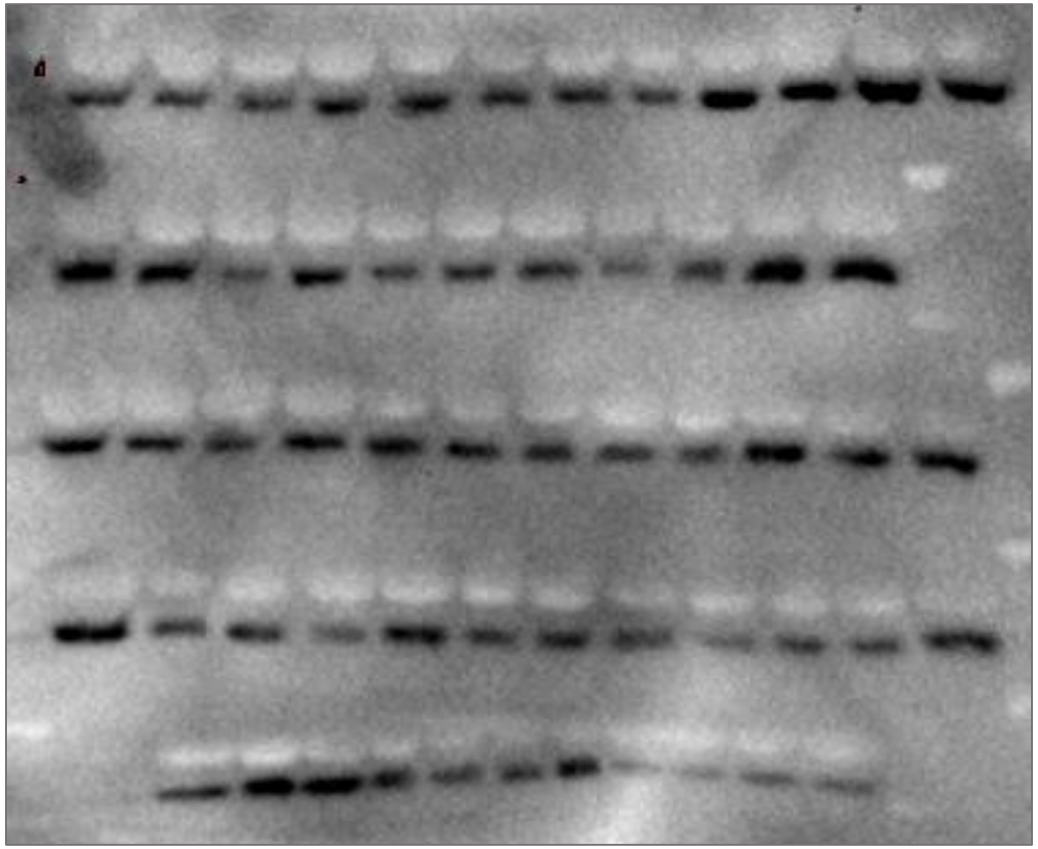

pATGL Ser406

Full unedited blots – overweight group

Subcutaneous adipose tissue (fig. 1)

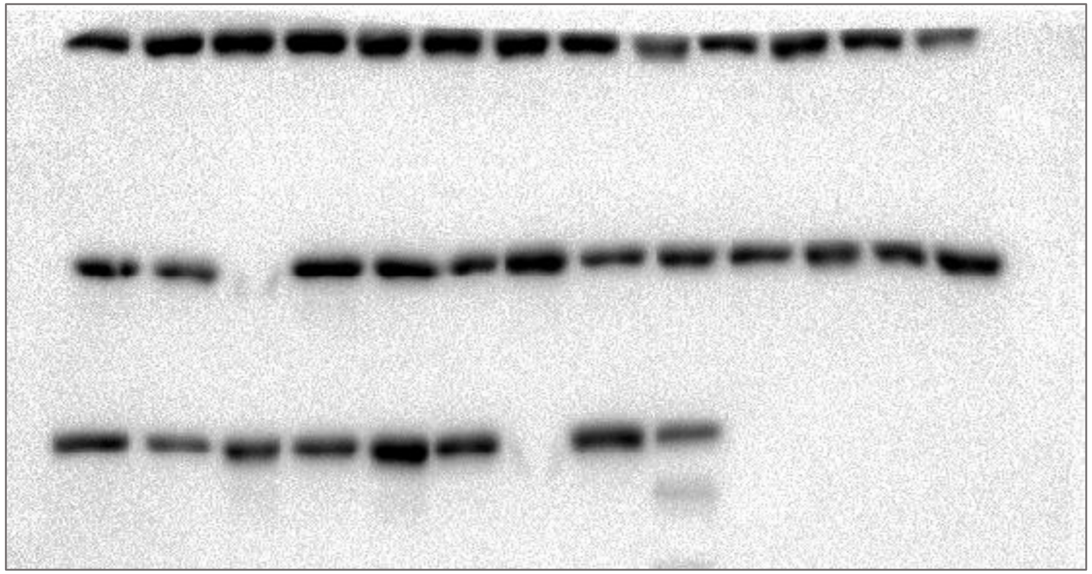

Full unedited blots – overweight group

HSL

Subcutaneous adipose tissue (fig. 1)

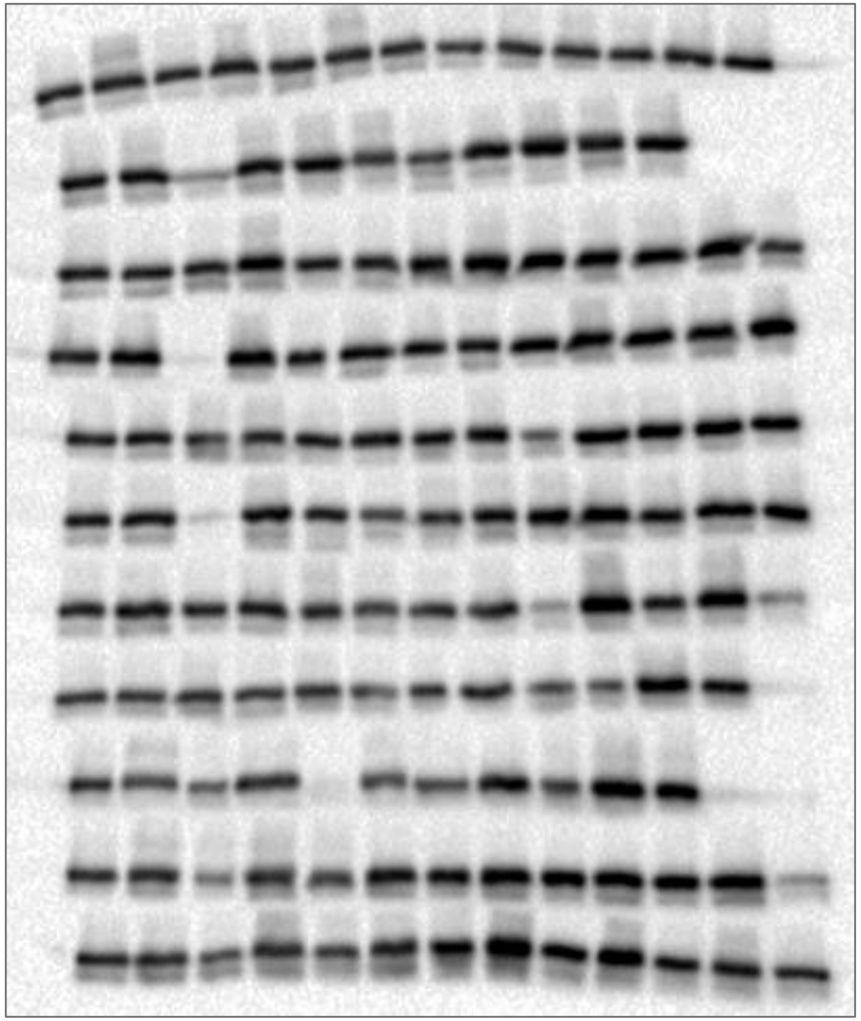

pHSL Ser660

Full unedited blots – overweight group

Subcutaneous adipose tissue (fig. 1)

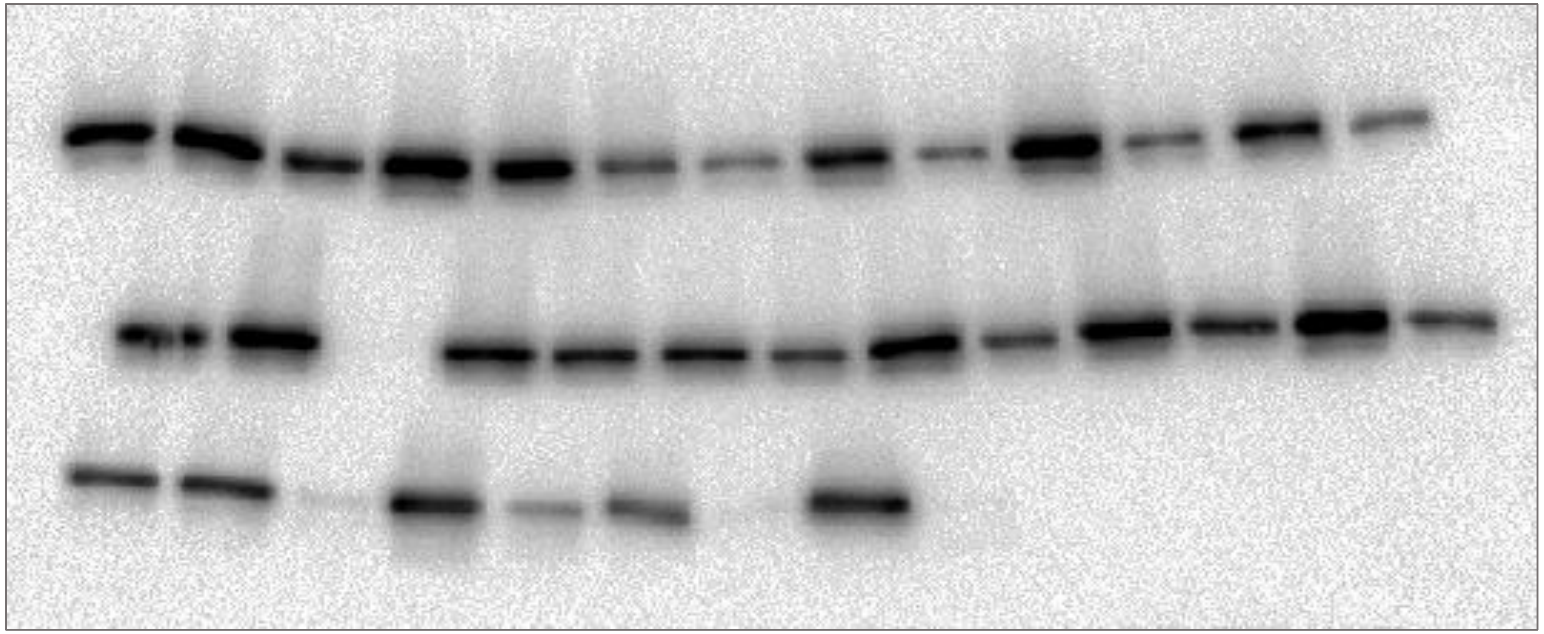

Full unedited blots – overweight group

Akt

Subcutaneous adipose tissue (fig. 1)

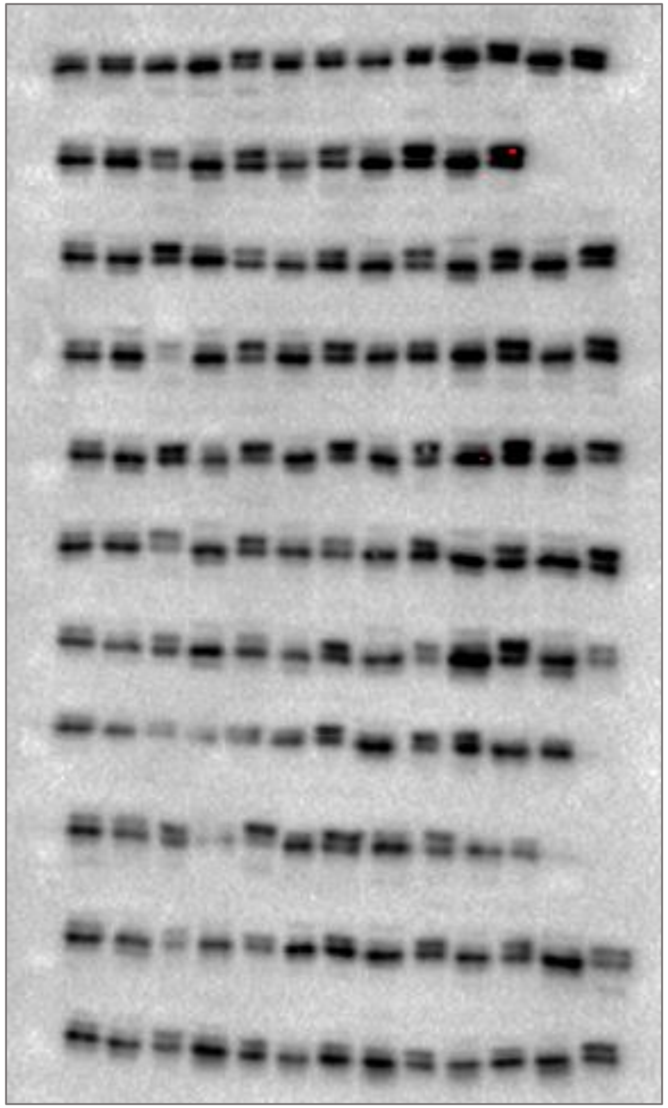

Full unedited blots – overweight group

pAkt Ser473

Subcutaneous adipose tissue (fig. 1)

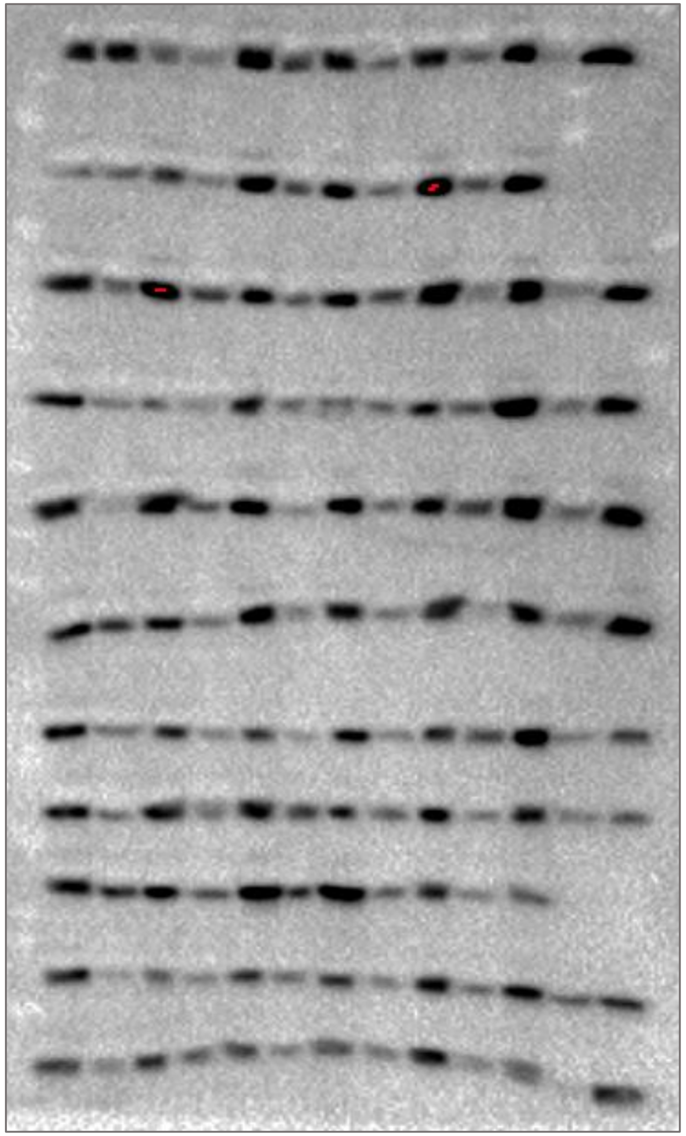

Full unedited blots – overweight group

IR

Skeletal muscle (fig. 1)

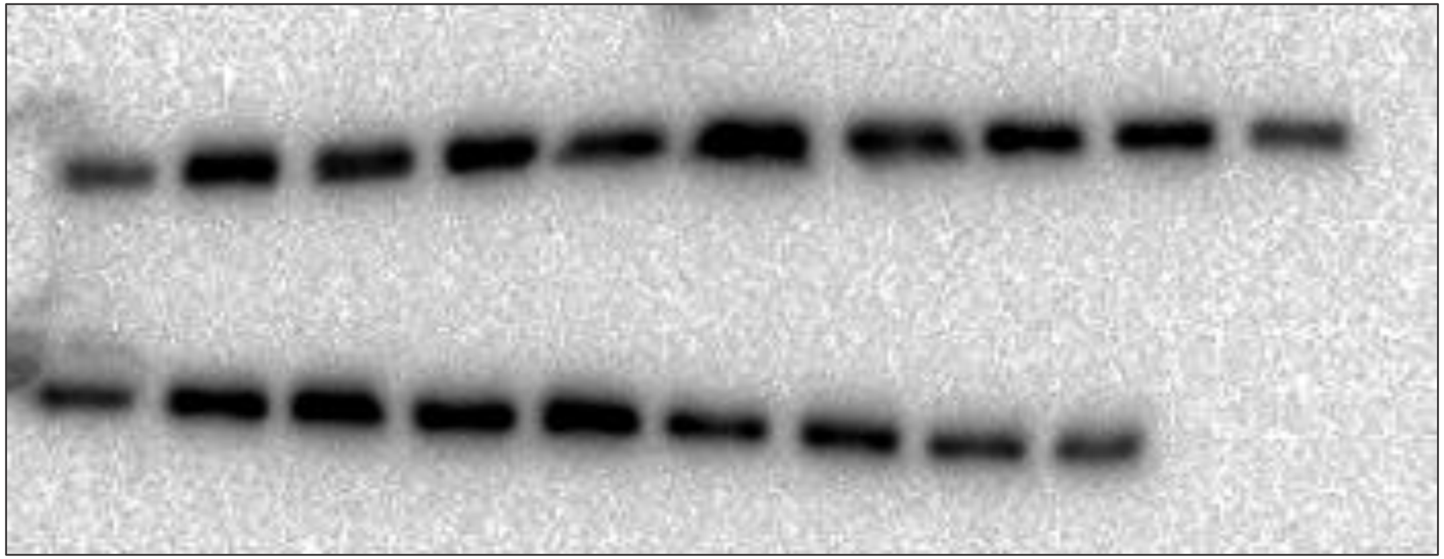

GLUT4

Full unedited blots – overweight group

Skeletal muscle (fig. 1)

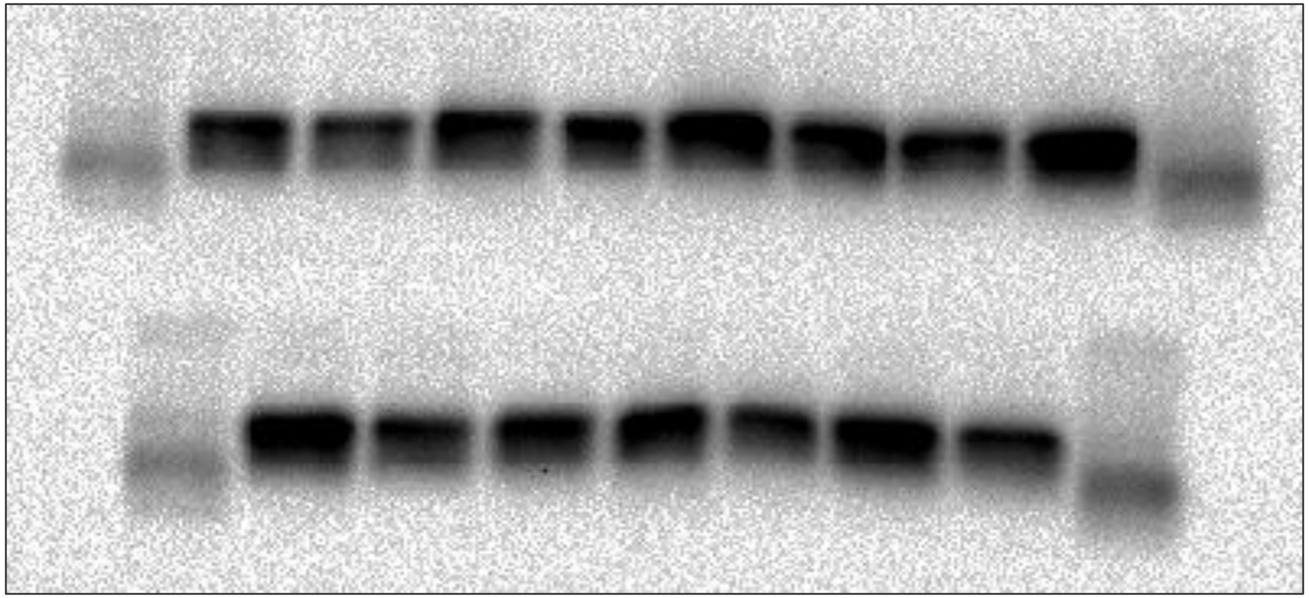

Full unedited blots – overweight group

HKII

Skeletal muscle (fig. 1)

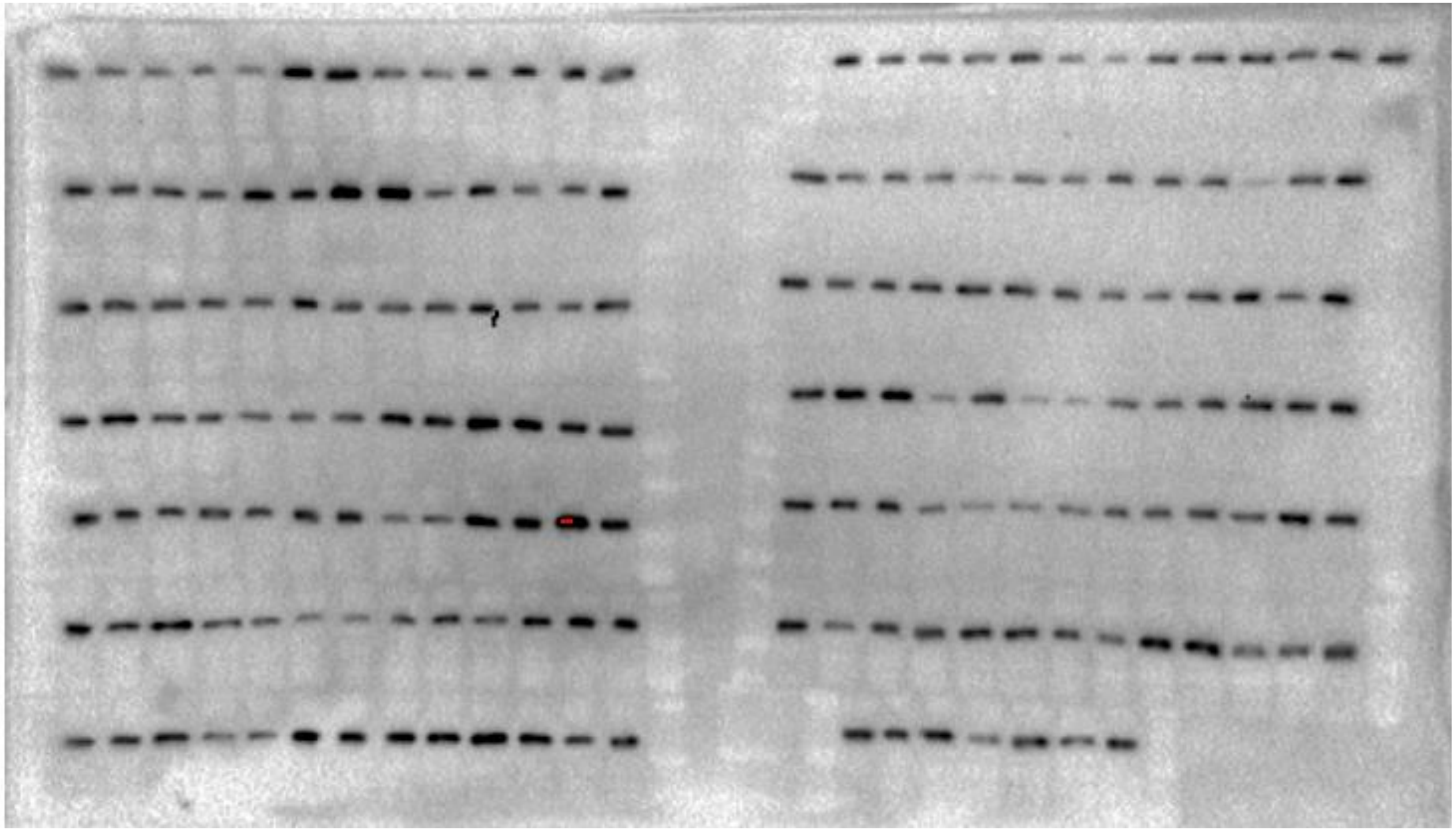

TBC1D4

Full unedited blots – overweight group

Skeletal muscle (fig. 1)

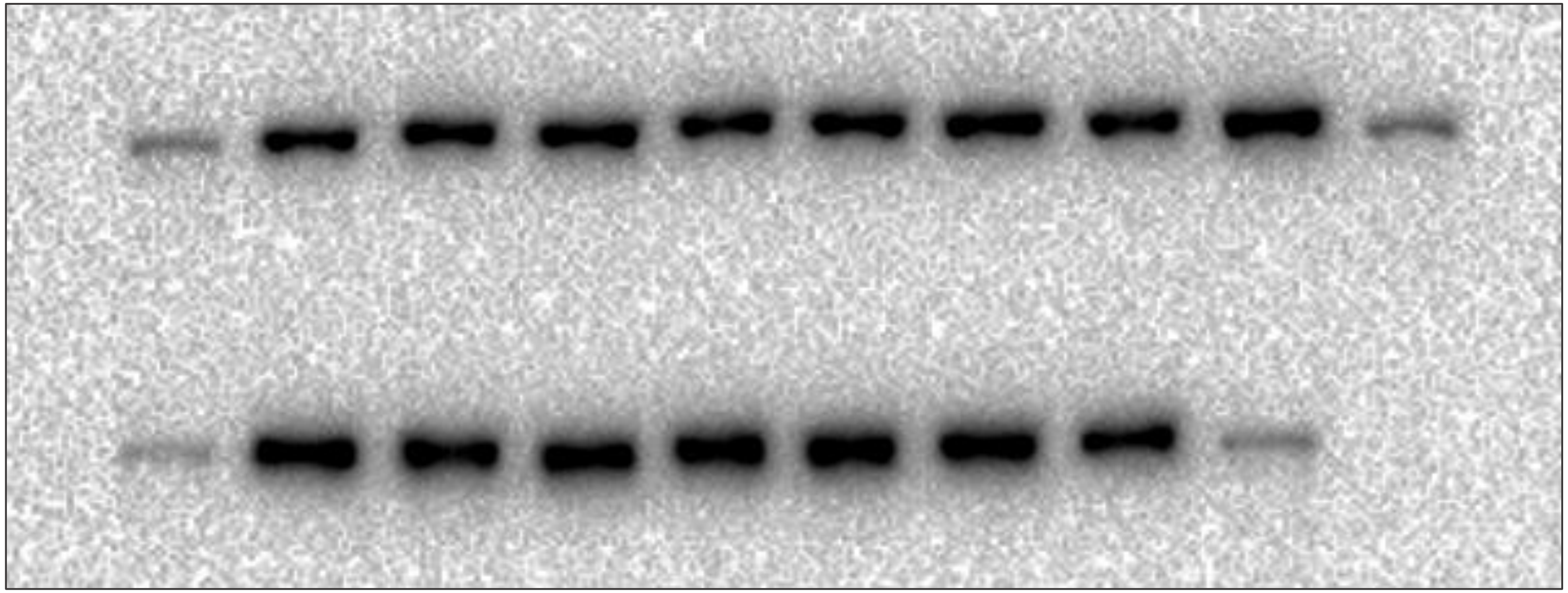

Full unedited blots – overweight group

CS

Skeletal muscle (fig. 1)

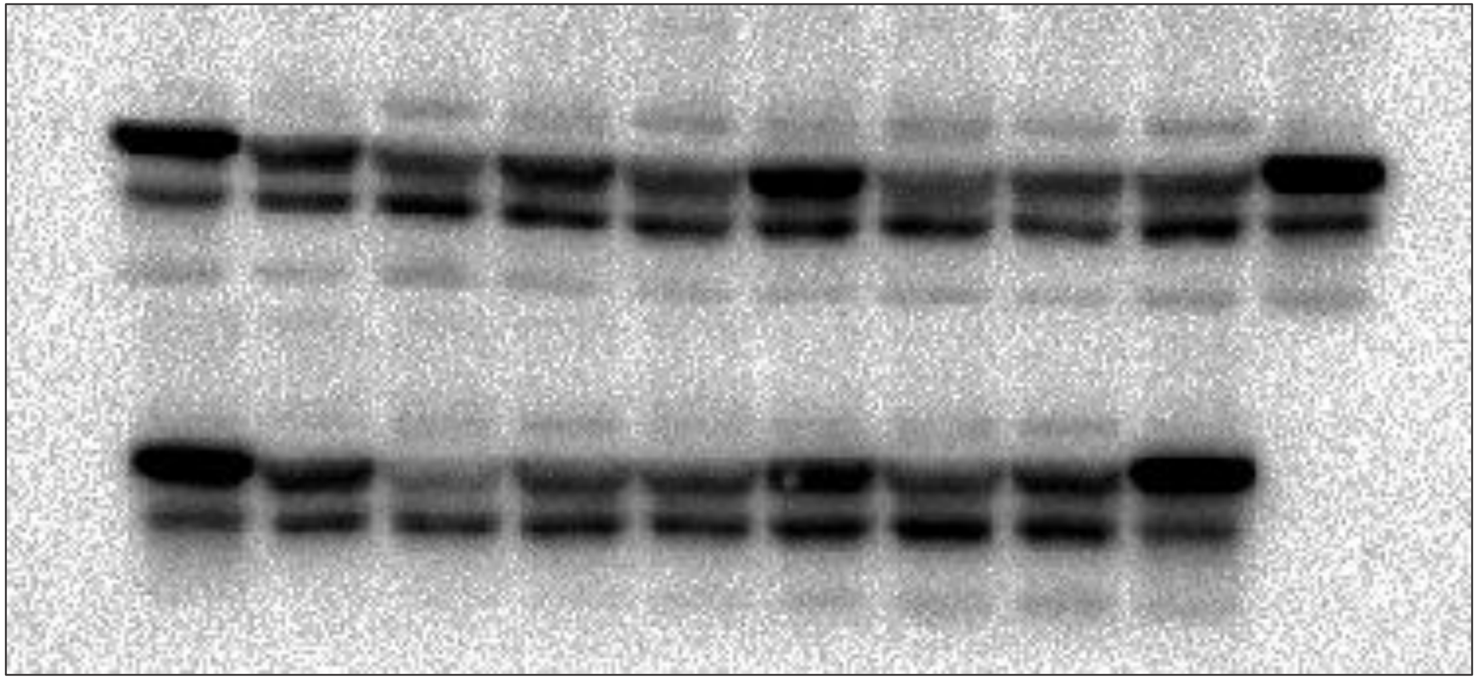

Full unedited blots – overweight group

PDH-E1α

Skeletal muscle (fig. 1)

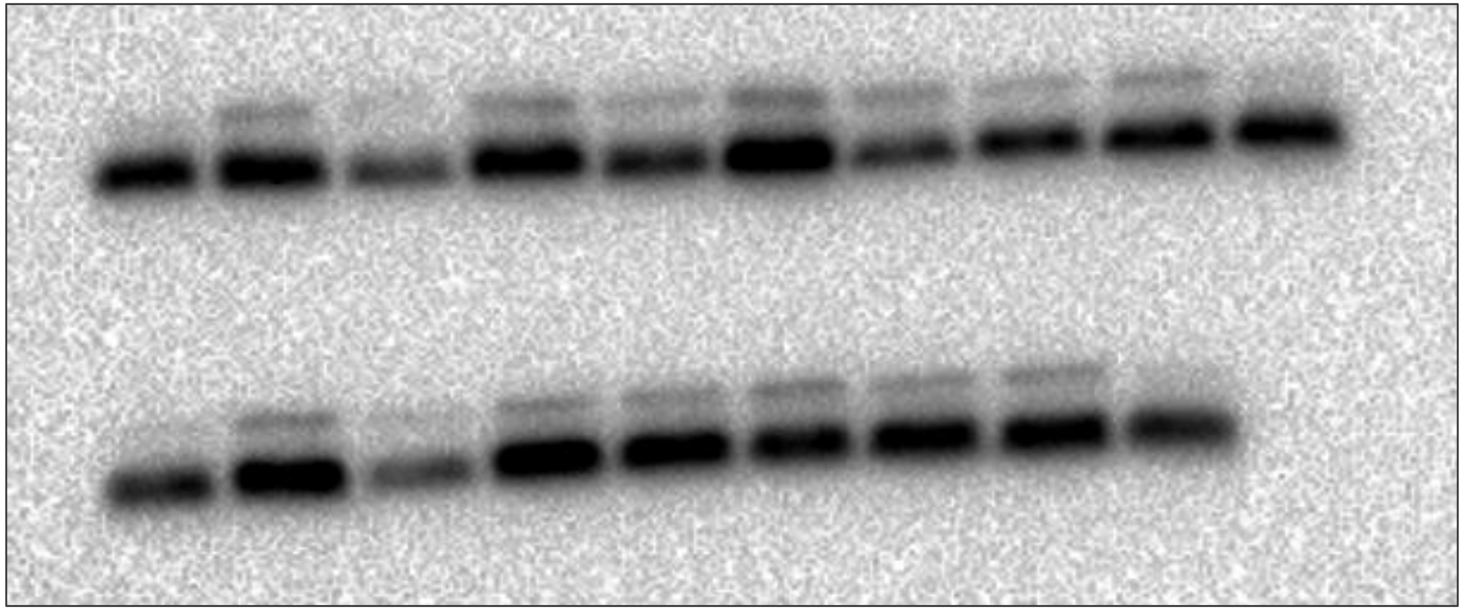

AMPKα2

Full unedited blots – overweight group

Skeletal muscle (fig. 1)

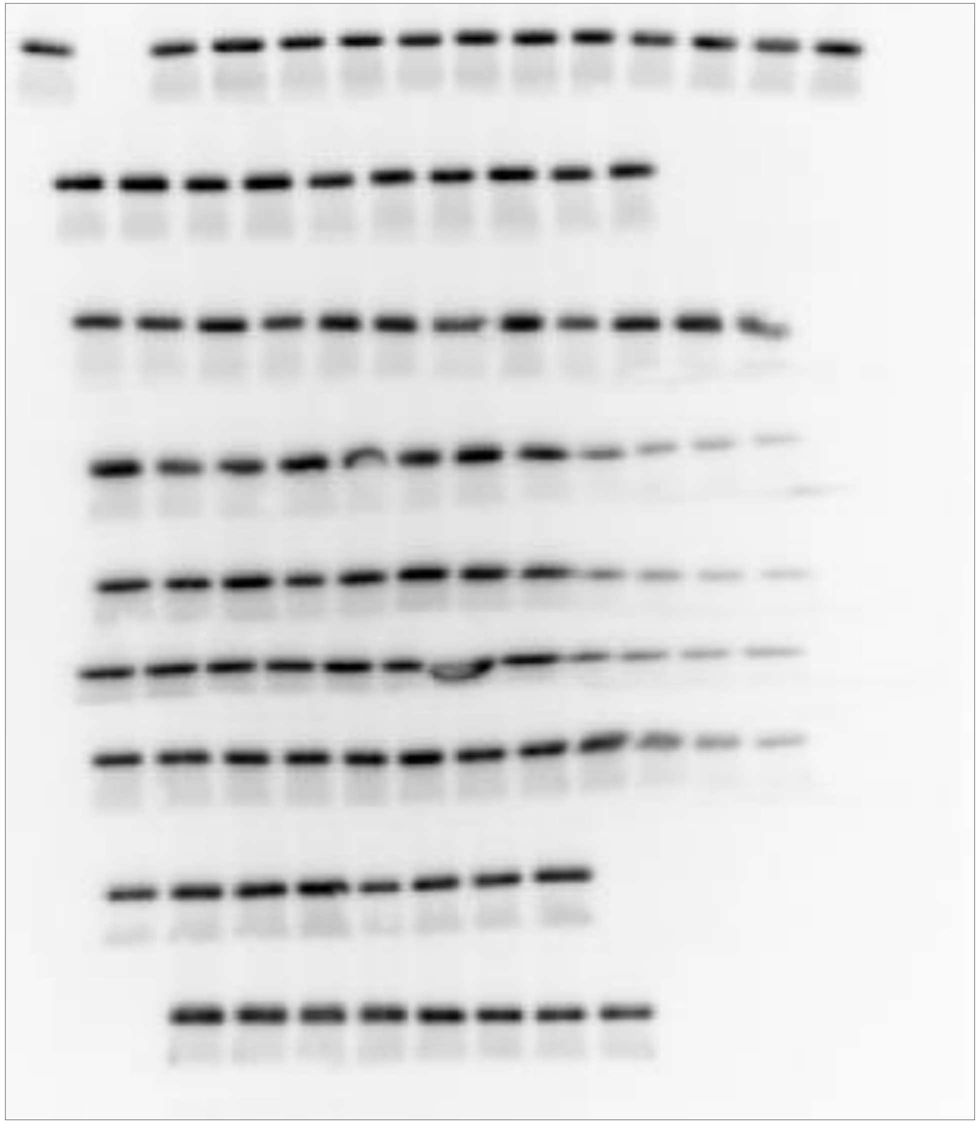

ACC

Full unedited blots – overweight group

Skeletal muscle (fig. 1)

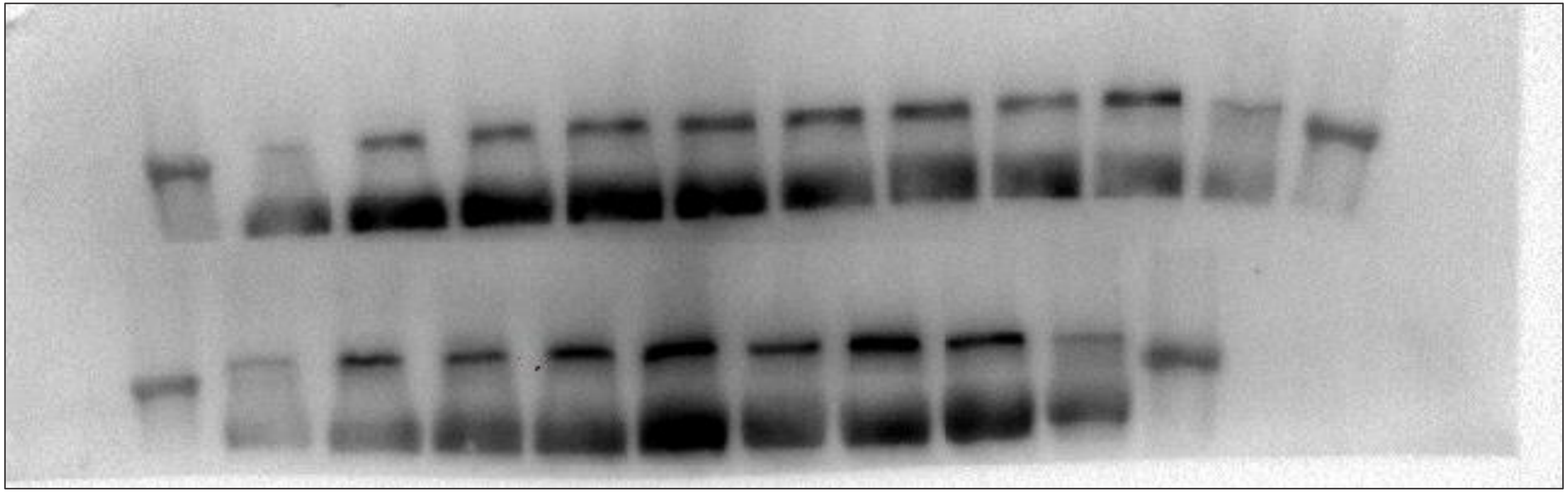

CD36

Full unedited blots – overweight group

Skeletal muscle (fig. 1)

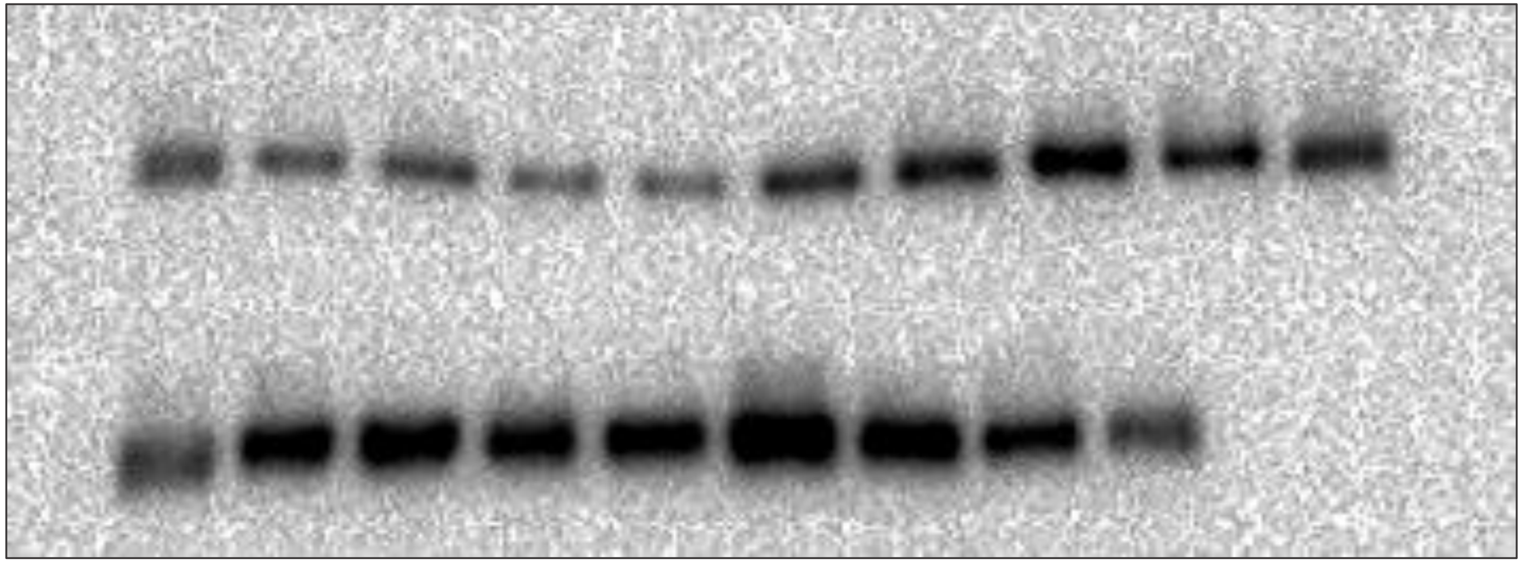

Full unedited blots – overweight group

ATGL

Skeletal muscle (fig. 1)

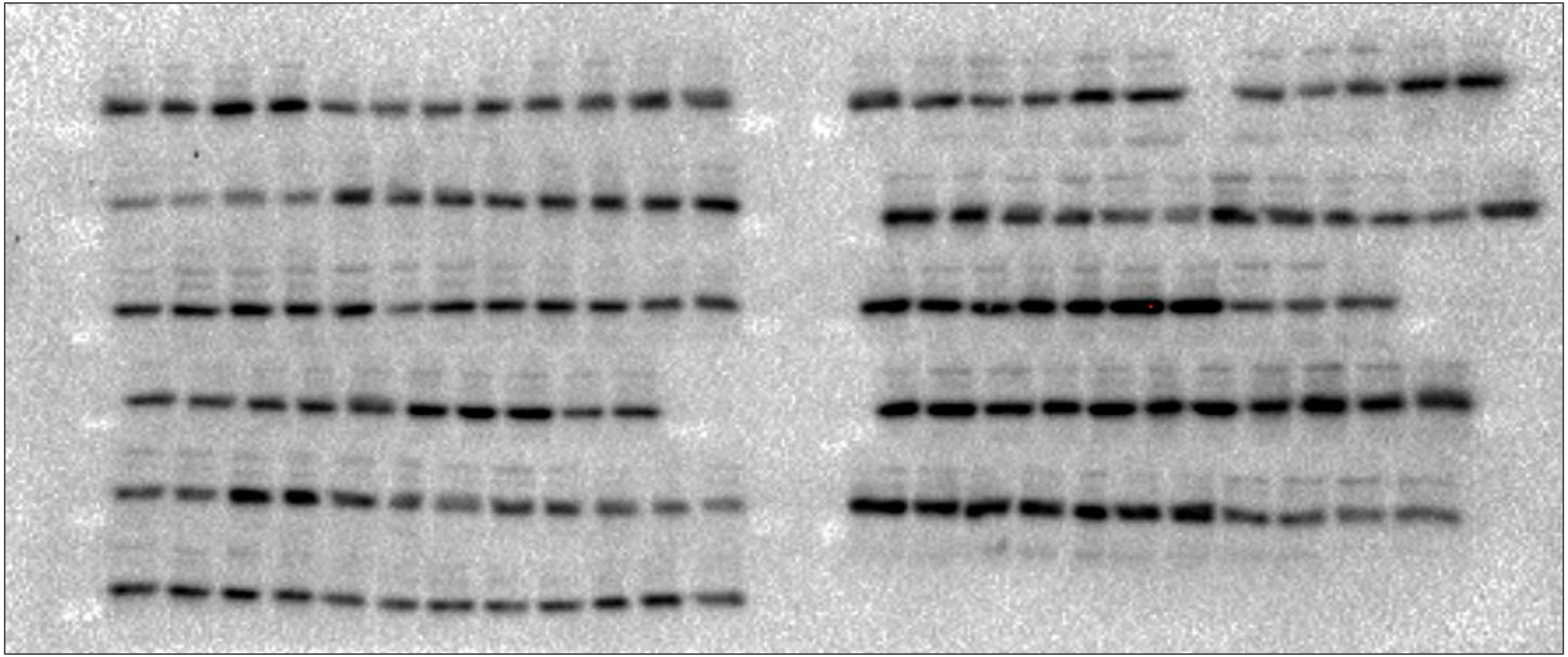

HSL

Full unedited blots – overweight group

Skeletal muscle (fig. 1)

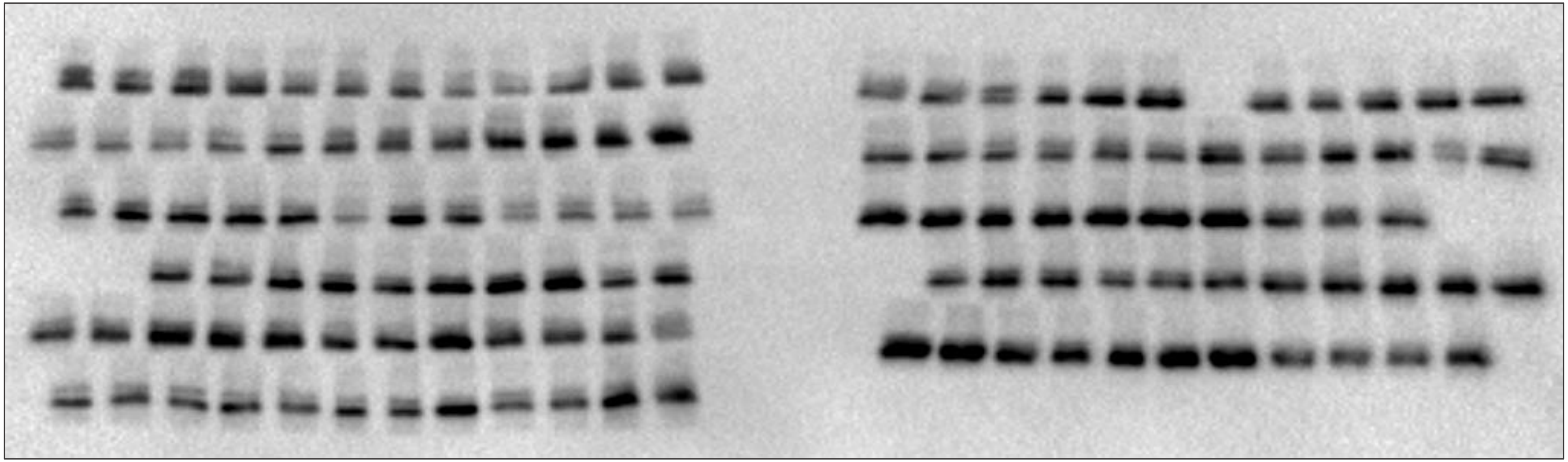

pHSL Ser660

Full unedited blots – overweight group

Skeletal muscle (fig. 1)

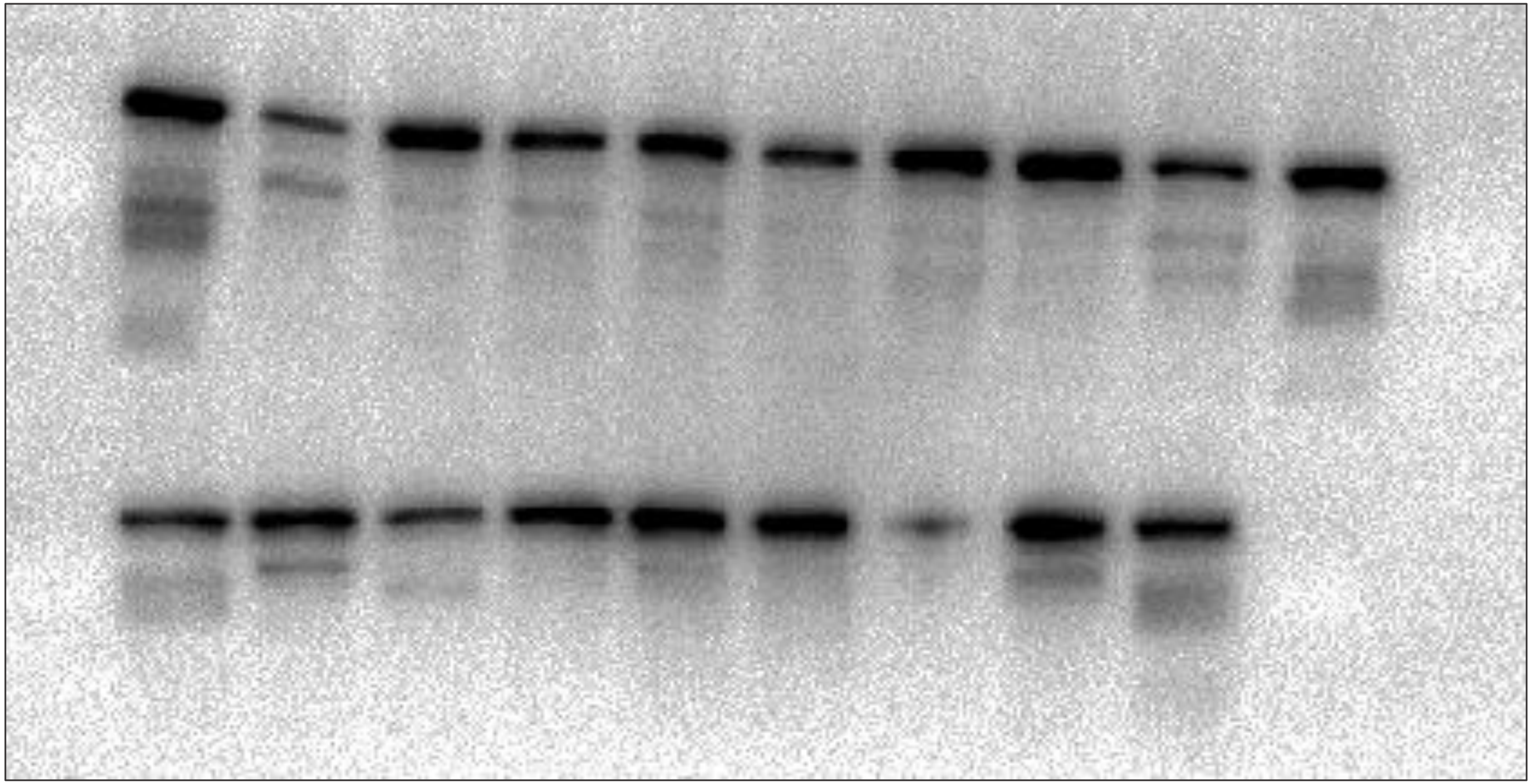

Full unedited blots – overweight group

Cav1

Skeletal muscle (fig. 1)

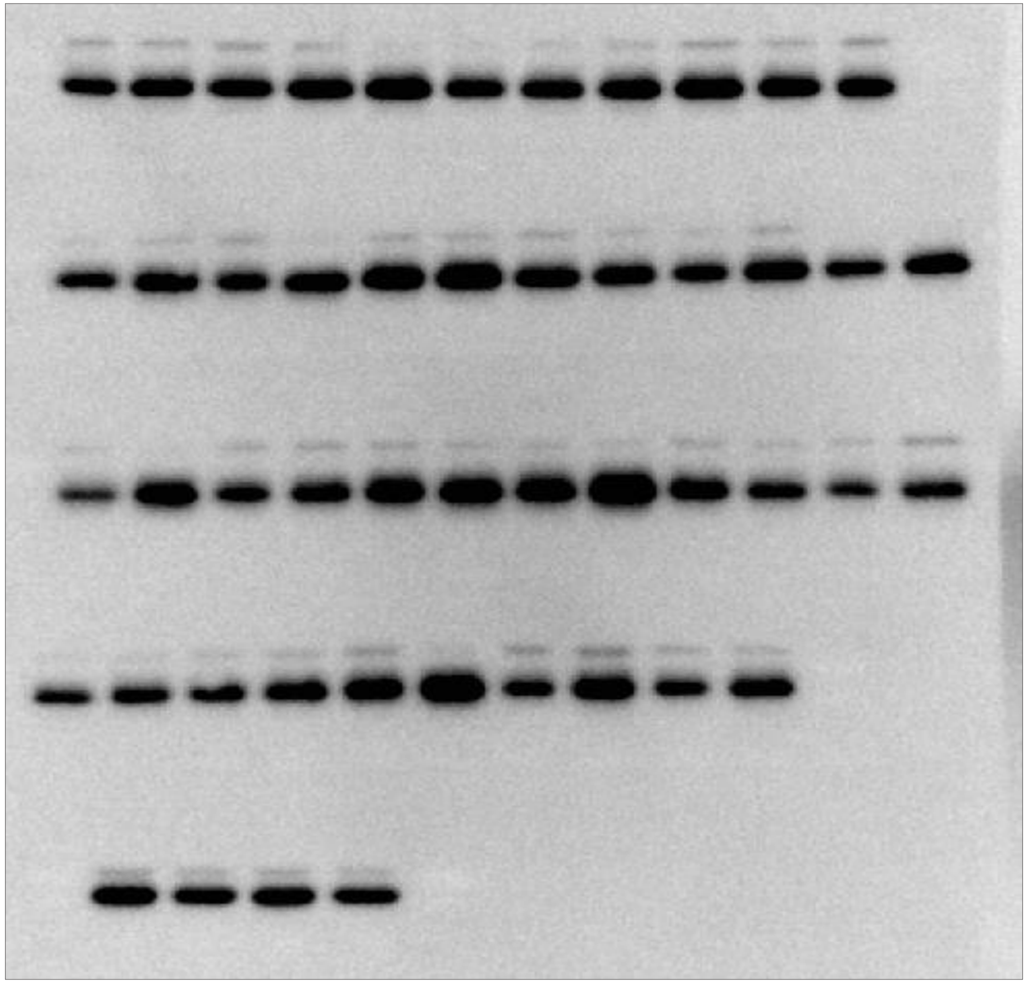

Full unedited blots – overweight group

Cav3

Skeletal muscle (fig. 1)

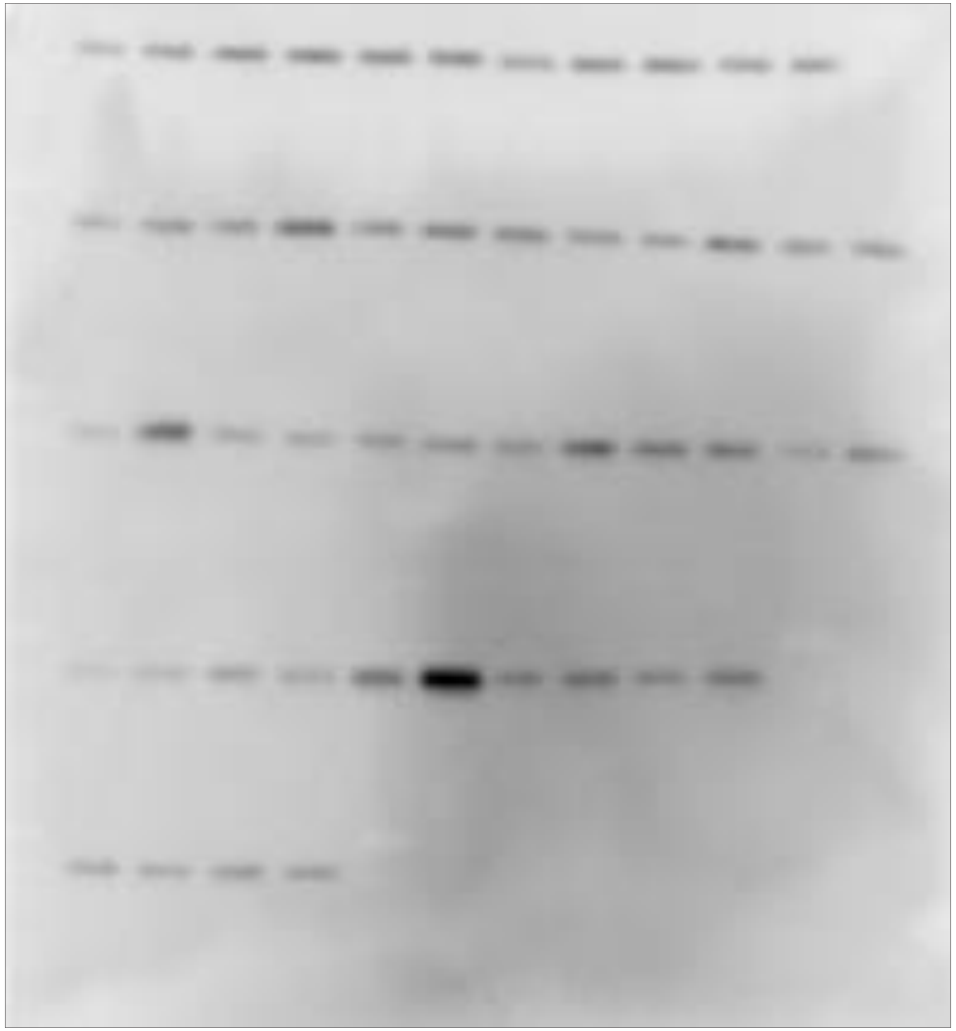

Akt

Full unedited blots – overweight group

Skeletal muscle (fig. 1)

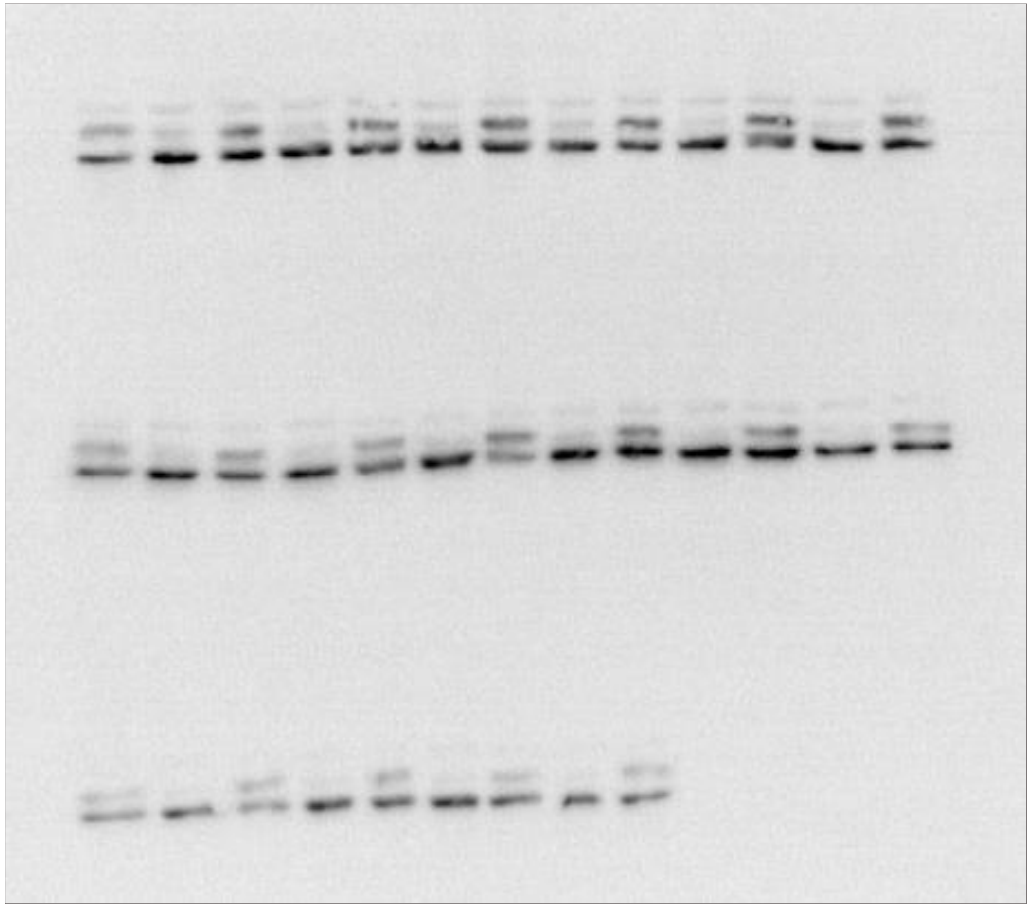

Full unedited blots – overweight group

pAkt Ser473

Skeletal muscle (fig. 1)

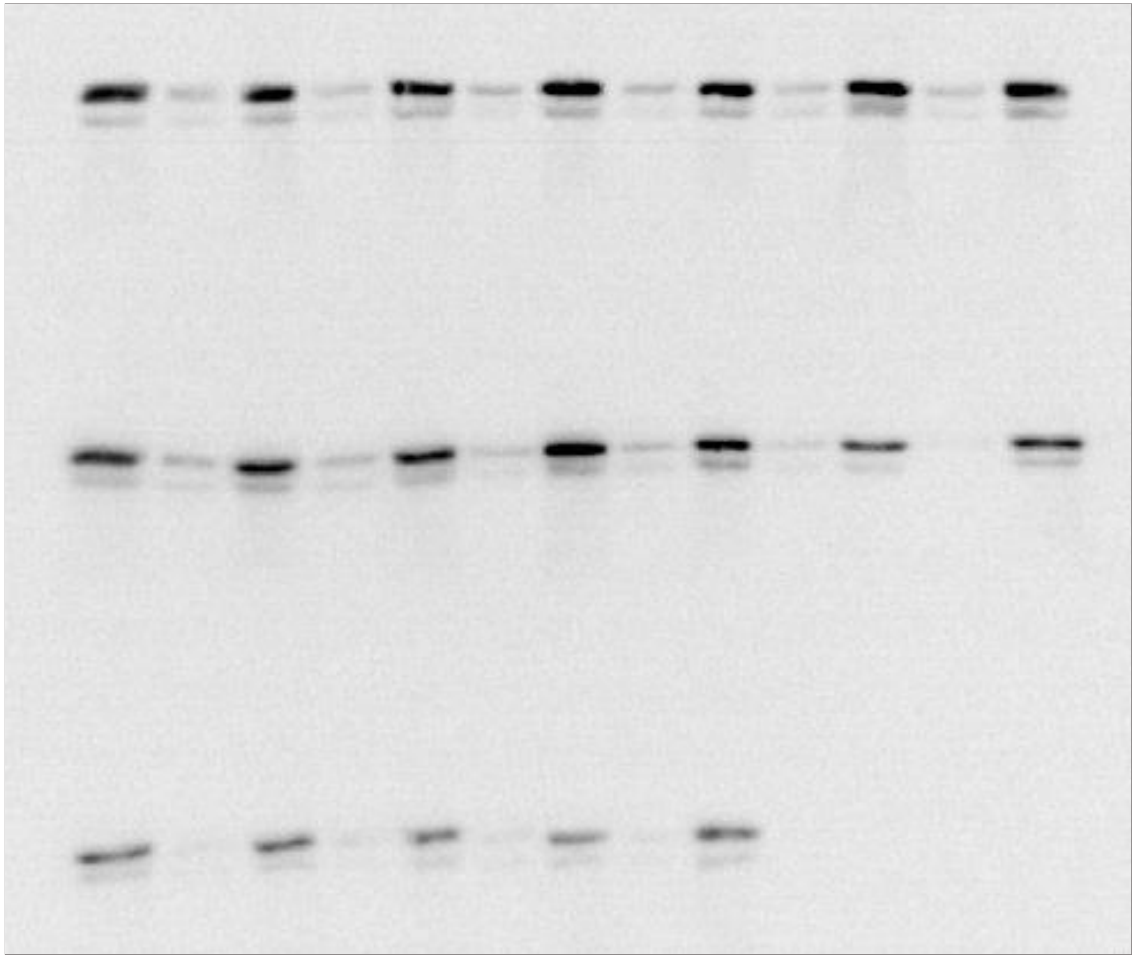

Supplement: Supplementary file 1 — Figure S1. [file FSB2-38-e23845-s004.pdf]
