# Supplementary material for: Greater molecular potential for glucose metabolism in adipose tissue and skeletal muscle of women compared with men
Source: FASEB J. 2024 Jul 31;38(15):e23845. doi: 10.1096/fj.202302377R (PMC11607633; doi:10.1096/fj.202302377R)

Beta-actin protein

Lean group.

Skeletal muscle housekeeping protein marker (fig. 1D)

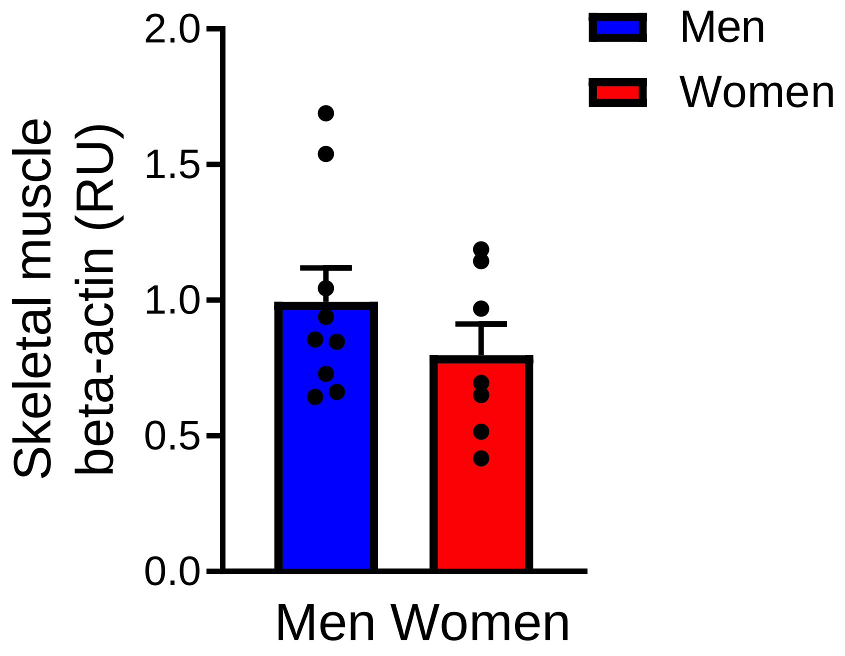

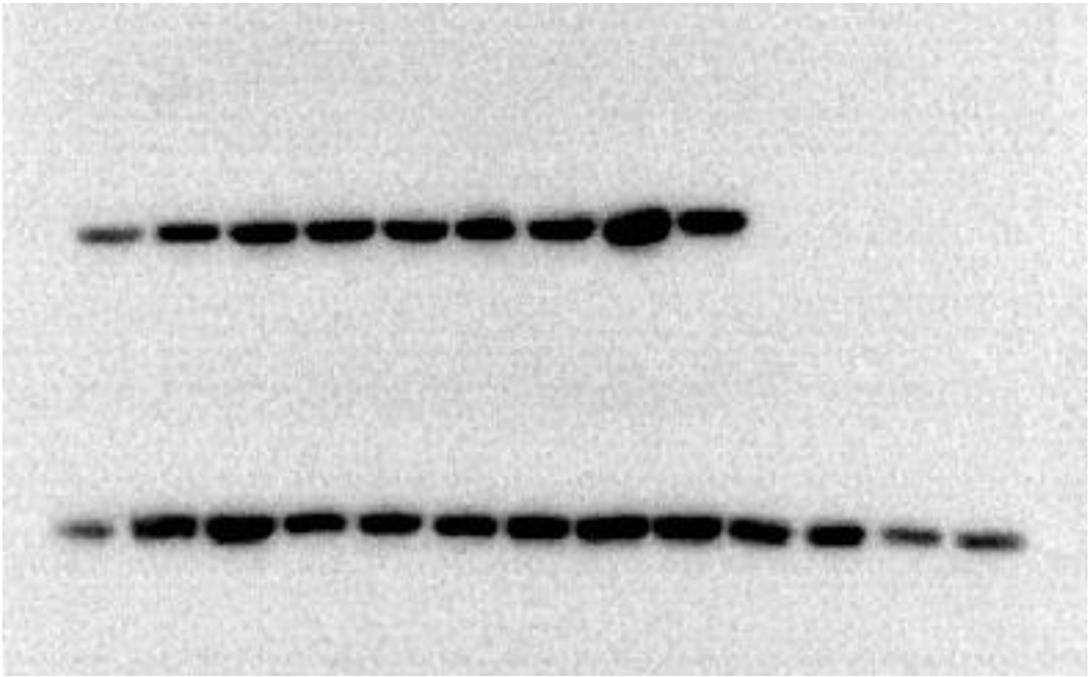

Beta-actin protein

obese group

Skeletal muscle housekeeping marker (fig. 3B)

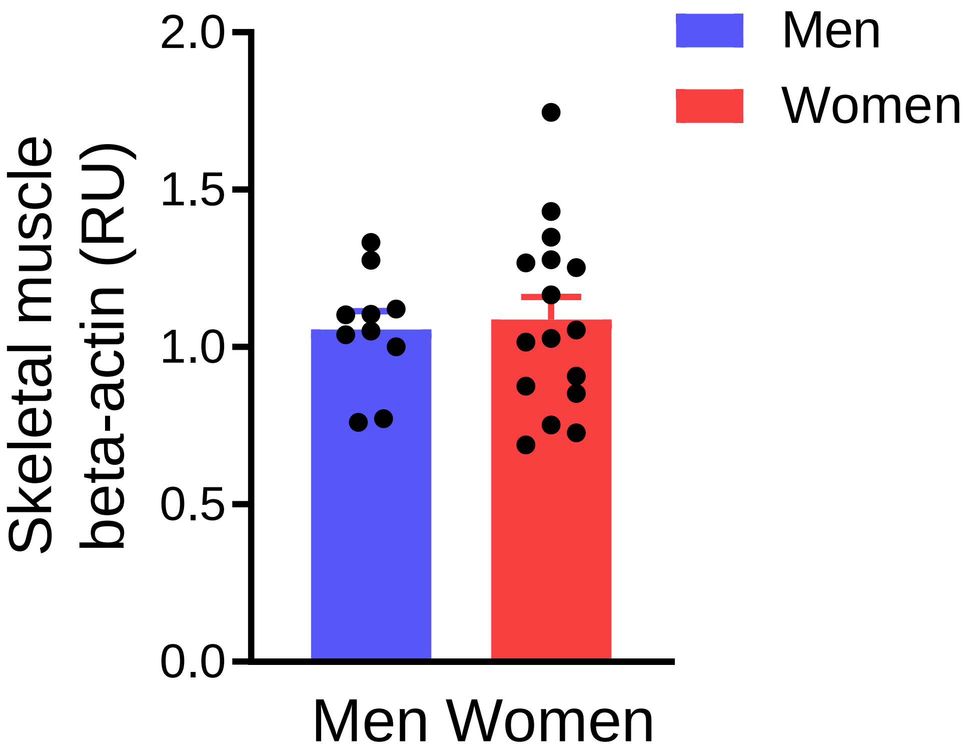

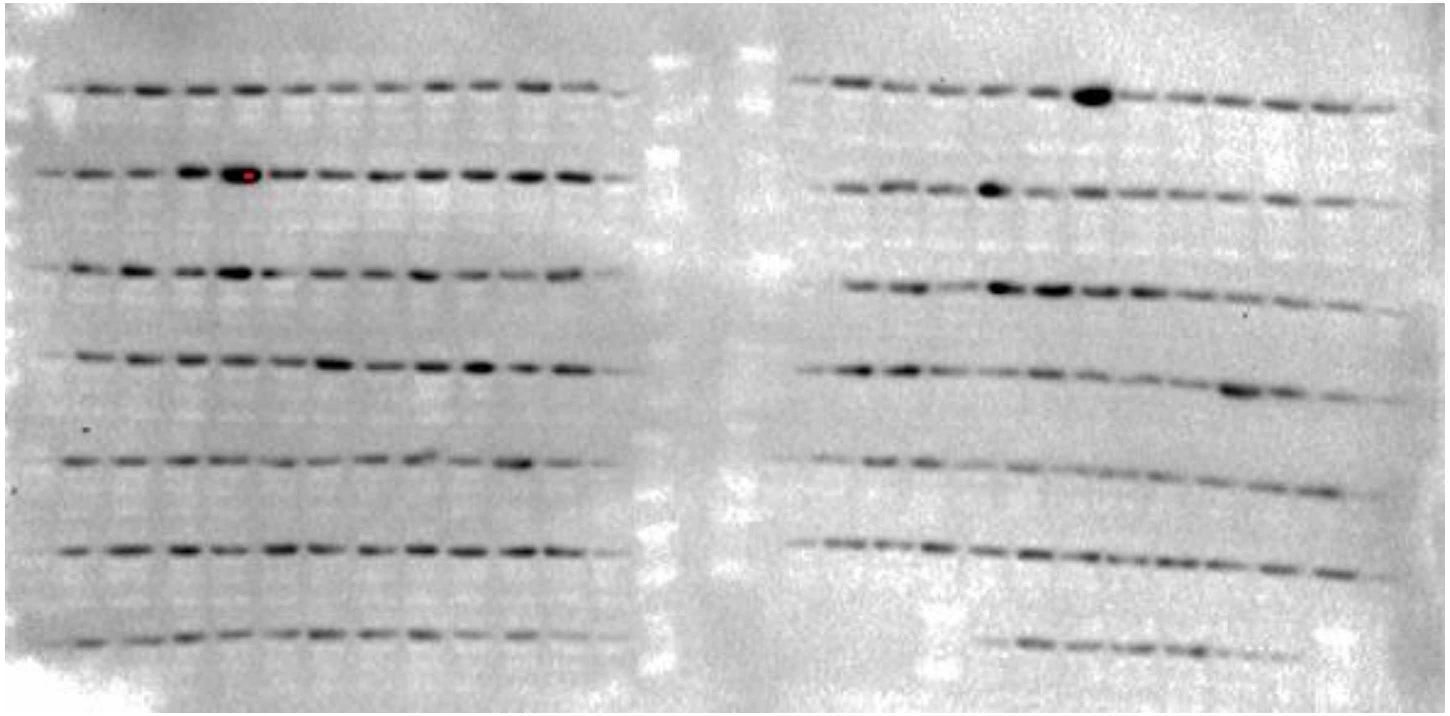

Total protein staining

Lean group,

Subcutaneous adipose tissue (fig. 1A)

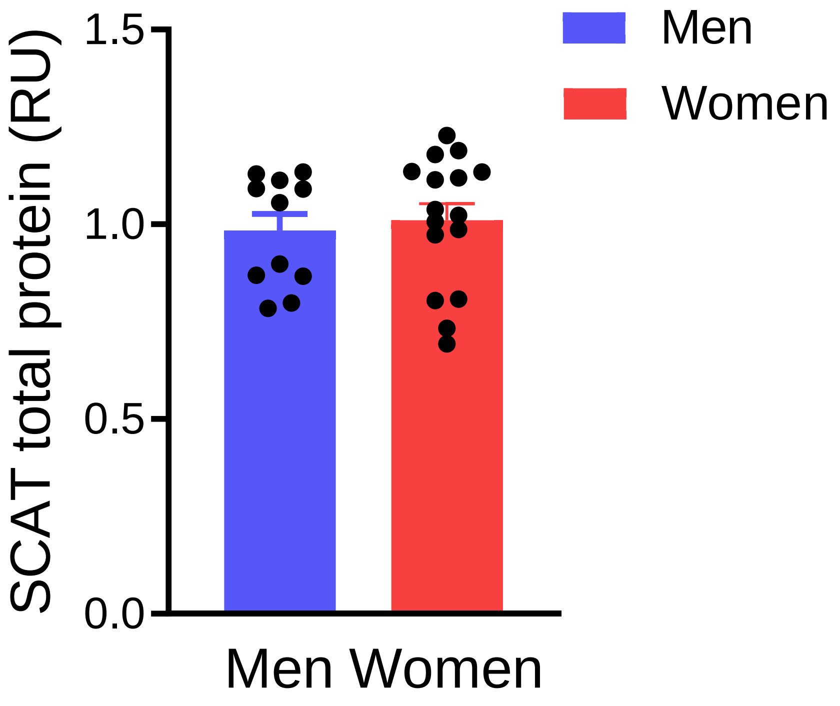

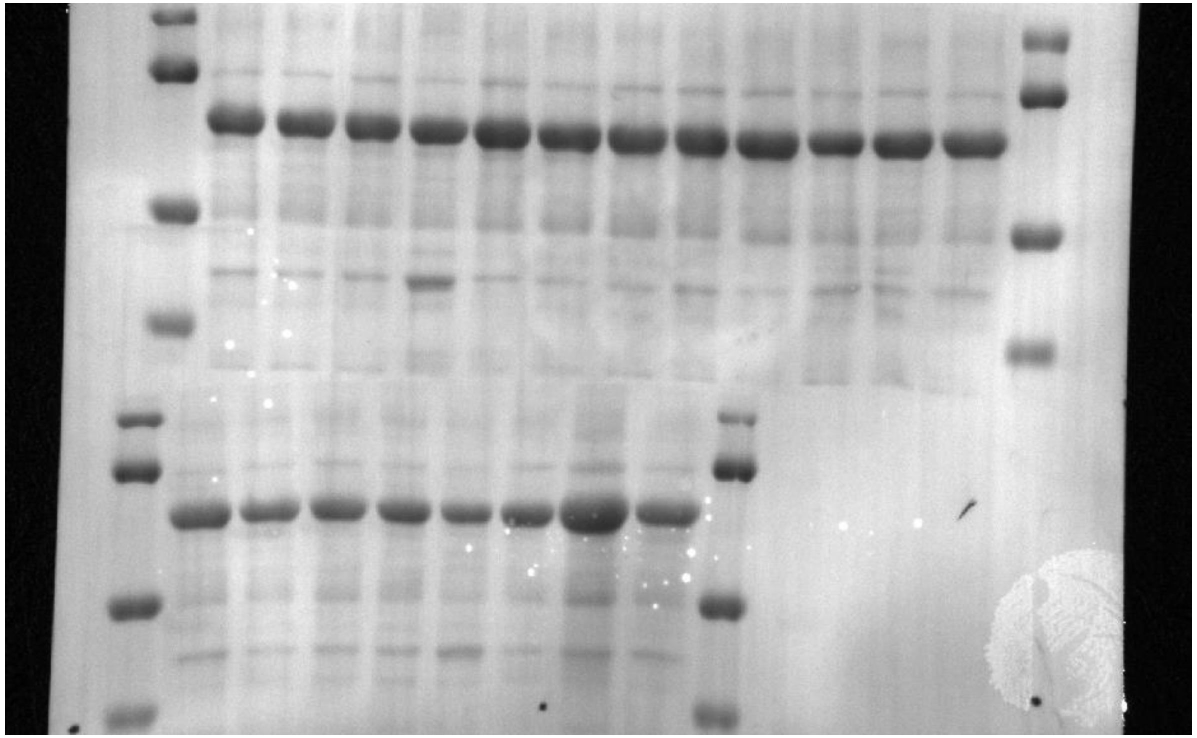

Total protein staining

obese group

Subcutaneous adipose tissue (fig. 3A)

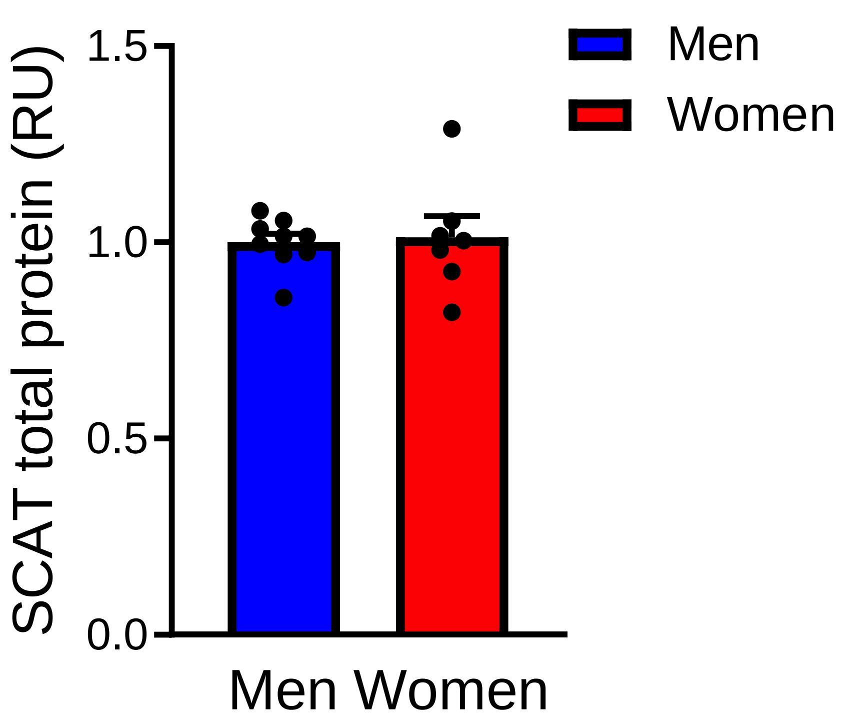

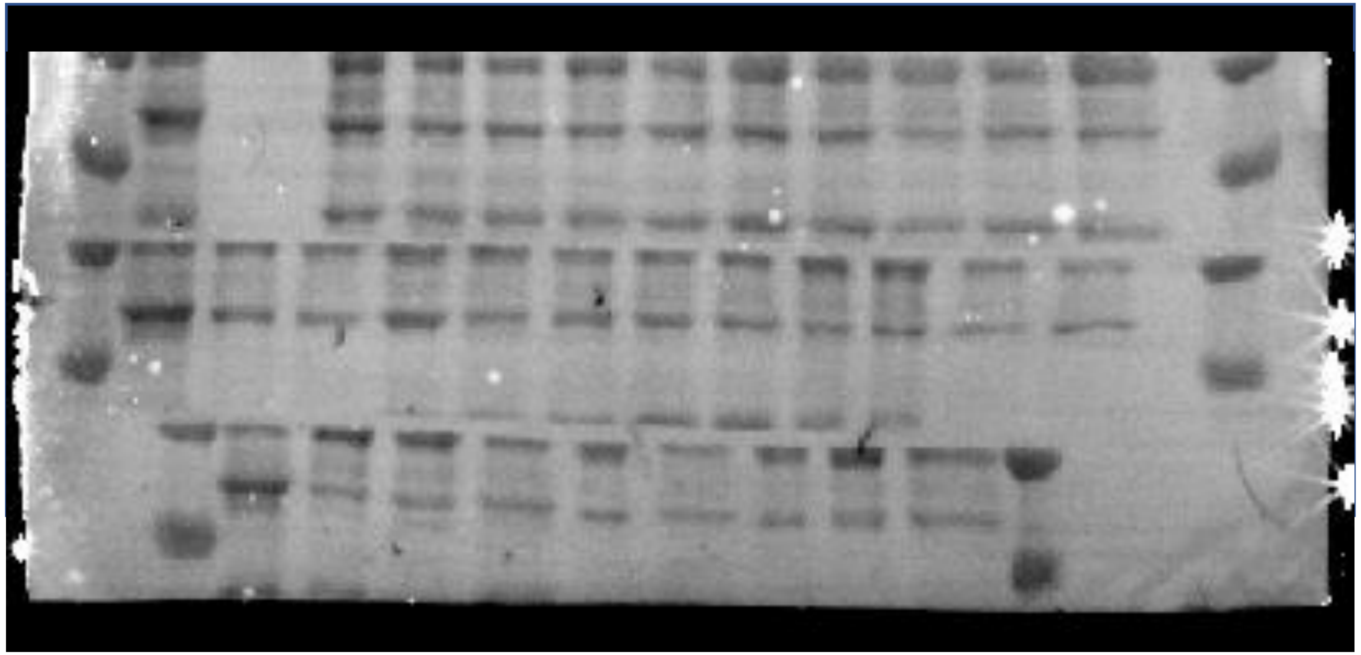

Supplement: Supplementary file 2 — Figure S2. [file FSB2-38-e23845-s001.pdf]
