# Supplementary material for: Greater molecular potential for glucose metabolism in adipose tissue and skeletal muscle of women compared with men
Source: FASEB J. 2024 Jul 31;38(15):e23845. doi: 10.1096/fj.202302377R (PMC11607633; doi:10.1096/fj.202302377R)
Supplement: Supplementary file 3 — Table S1. [file FSB2-38-e23845-s003.docx]

Table S1

|  |  |
| --- | --- |
| **Primary antibodies** | **Source** |
| anti-IR β subunit (CT3) | Donated by Dr. Ken Siddle, Cambridge University, UK |
| anti-GLUT4 | Thermo Scientific, US |
| anti-HKII | Alpha Diagnostics, US |
| anti-Akt2 | Cell Signaling Technology, US |
| anti-Akt Thr^473^ phosphorylation | Cell Signaling Technology, US |
| anti-TBC1D4 | Cell Signaling Technology, US |
| anti-CS | Abcam, UK |
| anti-HSL | Donated by Dr. C Holm, Lund University, SE |
| anti-HSL Ser^660^ phosphorylation | Cell Signaling technology, US |
| anti-CD36 | R&D Systems, UK |
| anti-PDH-E1𝛼 | Inhouse, Dr. H Pilegaard, Copenhagen University, DK |
| anti-AMPK𝛼2 | Donated by Dr. Hardie, Dundee University, US |
| anti-ACC | Streptavidin HRP antibody, Dako |
| anti-ATGL | Cell Signaling Technology, US |
| anti-ATGL Ser^406^ phosphorylation Abcam, UK | |
| anti-caveolin 1 | BD Transduction Laboratories, US |
| anti-caveolin 3 | BD Transduction Laboratories, US |
|  |  |

Table S1. List of the primary antibodies that were used for western blot analyses of human skeletal muscle and subcutaneous adipose tissue.
